# The cholesterol-binding protein NPC2 restrains recruitment of stromal macrophage-lineage cells to early-stage lung tumours

**DOI:** 10.15252/emmm.201404838

**Published:** 2015-07-16

**Authors:** Tamihiro Kamata, Hong Jin, Susan Giblett, Bipin Patel, Falguni Patel, Charles Foster, Catrin Pritchard

**Affiliations:** Department of Biochemistry, University of LeicesterLeicester, UK

**Keywords:** CCR1, lung adenoma, NPC2, tumour-associated macrophage-lineage cells, ^V600^^E^BRAF

## Abstract

The tumour microenvironment is known to play an integral role in facilitating cancer progression at advanced stages, but its function in some pre-cancerous lesions remains elusive. We have used the ^V600^^E^BRAF-driven mouse lung model that develop premalignant lesions to understand stroma–tumour interactions during pre-cancerous development. In this model, we have found that immature macrophage-lineage cells (IMCs) producing PDGFA, TGFβ and CC chemokines are recruited to the stroma of premalignant lung adenomas through CC chemokine receptor 1 (CCR1)-dependent mechanisms. Stromal IMCs promote proliferation and transcriptional alterations suggestive of epithelial–mesenchymal transition in isolated premalignant lung tumour cells *ex vivo*, and are required for the maintenance of early-stage lung tumours *in vivo*. Furthermore, we have found that IMC recruitment to the microenvironment is restrained by the cholesterol-binding protein, Niemann-Pick type C2 (NPC2). Studies on isolated cells *ex vivo* confirm that NPC2 is secreted from tumour cells and is taken up by IMCs wherein it suppresses secretion of the CCR1 ligand CC chemokine 6 (CCL6), at least in part by facilitating its lysosomal degradation. Together, these findings show that NPC2 secreted by premalignant lung tumours suppresses IMC recruitment to the microenvironment in a paracrine manner, thus identifying a novel target for the development of chemopreventive strategies in lung cancer.

## Introduction

Oncogenic mutations are prevalent in human cancers, and some are thought to represent early mutations that initiate and subsequently drive cancer development. *RAS* and *RAF* oncogenes are amongst the best-characterised driver oncogenes and are mutated in a significant proportion of human cancers, notably pancreatic (∼90%) and lung adenocarcinoma (∼30%) in the case of *KRAS* (Malumbres & Barbacid, 2003), and melanomas (∼50%) and thyroid cancers (∼30%) in the case of *BRAF* (Davies *et al*, 2002). In contrast to *KRAS*, the incidence of *BRAF* mutations in human lung adenocarcinoma is relatively low (Naoki *et al*, 2002), but nearly a half of *BRAF* mutations in this type of cancer are the most common ^*V600E*^*BRAF* mutation (COSMIC: http://cancer.sanger.ac.uk/cancergenome/projects/cosmic/), suggesting that ^V600E^BRAF contributes to lung carcinogenesis in some cases.

The mechanisms by which *KRAS* and *BRAF* oncogenes are involved in early-stage cancer development are beginning to be unravelled by analysis of genetically engineered mouse (GEM) models developing autochthonous tumours, especially for cancer types in which premalignant precursor lesions are difficult to access in humans. Lung adenocarcinoma is one such type of cancer in which atypical adenomatous hyperplasias (AAHs) are the purported precursor, but these early lesions are rarely diagnosed by non-invasive procedures (Gazdar & Brambilla, 2010). Instead, lung-specific expression of ^G12V^KRAS or ^V600E^BRAF in GEM models has provided evidence that activation of these oncogenes initially induces the formation of benign proliferative lesions after which the lesions enter a state of stable cell cycle arrest termed oncogene-induced senescence (OIS) (Collado *et al*, 2005; Dankort *et al*, 2007). While ^V600E^BRAF-driven early lung lesions with OIS rarely progress to adenocarcinoma (Dankort *et al*, 2007), ^G12V^KRAS-driven alveolar hyperplasias progress into malignant adenocarcinomas more frequently (Mainardi *et al*, 2014), which correlates well with the mutation spectrum of human lung adenocarcinomas. However, malignant progression of early lung lesions in the ^V600E^BRAF model can be facilitated by mutations in key genes including depletion of tumour suppressor *TRP53* (Dankort *et al*, 2007) or constitutive activation of *β-catenin* (Juan *et al*, 2014), indicating that these early lesions are indeed precursors for adenocarcinomas.

Although the difference between the ^G12V^KRAS and ^V600E^BRAF models with regard to malignant progression could be explained by the nature of intracellular signalling cascades activated by each oncoprotein (Trejo *et al*, 2012, 2013), cell-extrinsic inflammatory responses have also been shown to contribute to malignant progression of ^G12V^KRAS-driven early lesions, at least in a pancreatic cancer model (Guerra *et al*, 2011). In contrast, it still remains unclear how inflammatory or other environmental factors could influence the behaviour of ^V600E^BRAF-driven early-stage tumours with OIS. Whereas the microenvironment of advanced cancers, composed of a panoply of different cell types including hematopoietic (immune) cells, vascular components and activated fibroblasts, creates a pro-tumourigenic environment (Whiteside, 2008), there is growing evidence that immune cells in the microenvironment of pre-cancerous lesions could play a tumour-suppressive rather than tumour-promoting role. For example, CD4^+^ T-cell-mediated adaptive immunity in concert with monocytes/macrophages has been shown to execute the clearance of oncogenic NRAS-induced pre-cancerous lesions in the liver (Kang *et al*, 2011). In this model, early hepatic lesions exhibited biochemical characteristics of OIS including secretion of inflammatory chemo-cytokines (Kuilman & Peeper, 2009), which likely contributed to the recruitment of the immune cells to the microenvironment. Such a suppressive microenvironment against pre-cancerous OIS lesions could potentially contribute to the less frequent malignant progression of ^V600E^BRAF-driven senescent lung adenomas.

To investigate the role of the tumour microenvironment in ^V600E^BRAF-driven premalignant lesions, we have taken advantage of our ^V600E^BRAF-driven autochthonous GEM model in which premalignant papillary adenomas accumulate in the lung. We show here that immature macrophage-lineage cells (IMCs) are recruited to the stroma of senescent lung adenomas through CCR1-dependent mechanisms. Unexpectedly, stroma IMCs are found to play a pro-tumourigenic role *in vivo* since the suppression of IMC recruitment through CCR1 inhibition profoundly decreases tumour burden. Furthermore, in a screen for proteins secreted from ^V600E^BRAF-expressing premalignant tumour cells, we identified the cholesterol-binding protein Niemann-Pick type C2 (NPC2). Our studies with NPC2 show it is secreted at high levels even at the pre-senescent stage, and is incorporated by IMCs wherein it regulates intracellular cholesterol levels and inhibits secretion of the CCR1 ligand, CC chemokine 6 (CCL6). This results in the suppression of IMC accumulation at the pre-senescent stage. Overall, the data point to a novel role of NPC2 in regulation of the pro-tumourigenic microenvironment.

## Results

### ^V600E^BRAF induces the formation of senescent lung adenomas

**Figure 1:**
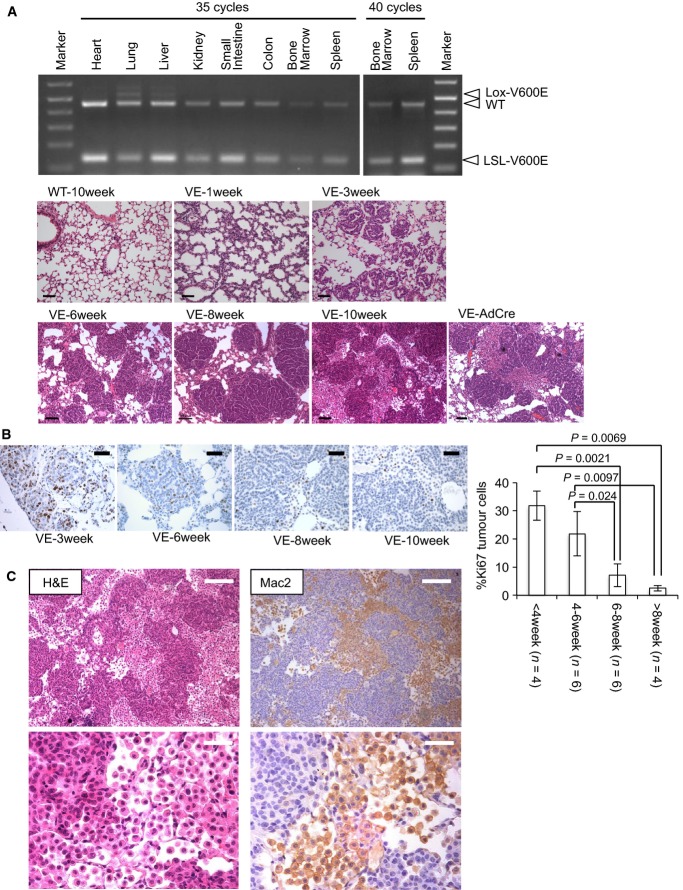
Characterisation of lung tissue expressing ^V600^^E^BRAF (Top) PCR detection of the recombined *Braf*^*Lox−V600E*^ allele (Lox-V600E) in BVE mouse tissues at 8 weeks of age without tamoxifen induction. Substantial recombination is observed in the lung, while weaker recombination is also detected in the liver. No recombination was detected in hematopoietic tissues (bone marrow and spleen) even after 40 cycles of amplification (right). (Bottom) H&E staining of lung sections from wild-type (WT, 10 weeks of age), BVE (1–10 weeks of age) and *Braf*^*+/*^^*LSL*^^*−V600E*^ mouse 8 weeks after nasal delivery of AdCre is indicated below. ^V600^^E^BRAF expression induced by two different methods causes similar pathology showing papillary adenomas accompanied by stroma development. Scale bars, 100 μm.

Conditional (Cre-loxP-regulated) knockin mice for oncogenic ^V600E^BRAF and ^G12V/G12D^KRAS have been previously generated by our group and others, and induction of oncogene expression in the lung in both models has been shown to induce premalignant lesions that up-regulate the expression of senescence markers (Collado *et al*, 2005; Dankort *et al*, 2007). To obtain an easily manipulated model for biochemical investigations, we utilised *Braf*^*+/LSL−V600E*^*;CreER*^*™*^ (BVE) mice since these developed large numbers of pulmonary papillary adenomas in 100% of mice by spontaneous recombination of the *Braf* allele in the lung without tamoxifen induction (Fig1A). Histologically, these tumours were identical to those induced by nasal administration of AdCre (Fig1A). As previously reported for AdCre-induced tumours (Dankort *et al*, 2007), pulmonary adenomas in BVE mice exhibited signs of OIS at later stages, including significant loss of Ki67 expression (Fig1B). These tumours did not stain for senescence-associated β-galactosidase or express p16^INK4a^ or p19^ARF^ at detectable levels but were positive for p21^CIP1^ and γH2AX (Supplementary Fig S1), suggesting that DNA-damage responses could be the major cause for the cell cycle arrest. Although most tumour cells at 10 weeks or later were negative for Ki67, a small population (2%) of Ki67^+^ tumour cells remained detectable at this later time point (Fig1B).

Ki67 immunostaining of lung sections from BVE mice at 3–10 weeks of age. Scale bars, 25 μm. The right bar graph summarises %Ki67^+^ cells in tumours at different ages post-partum. Four to six mice were analysed for each age group as indicated, and more than 3,500 tumour cells per mouse were evaluated. The data represent mean ± SD.

Representative H&E staining and Mac2 immunohistochemistry of serial lung sections from 10-week-old BVE mice at low (upper panels) and high (lower panels) magnifications. Scale bars, 100 μm (upper panels) or 25 μm (lower panels). Source data are available online for this figure.

For the most part, wherever possible, we utilised the CreER™ strain to induce ^V600E^BRAF expression rather than AdCre because of the known inflammatory phenotypes associated with AdCre delivery to the lung, even in wild-type mice (Mainardi *et al*, 2014). AdCre was only utilised in situations when the analysis required longer-term survival of mice (see below).

### Immature macrophage-lineage cells expressing ^WT^BRAF are present in the stroma

Interestingly, the development of adenomas in both the BVE and *Braf*^*+/LSL−V600E*^*/*AdCre models was accompanied by the recruitment of non-tumour cells to the stroma (Fig1A and C). The majority of these cells displayed an oval-shaped morphology with round nuclei and a relatively low nuclear/cytoplasm ratio reminiscent of myeloid-lineage hematopoietic cells (Fig1C). Stroma-specific staining with a myeloid marker Mac2 also supported their myeloid origin (Fig1C).

**Figure 2:**
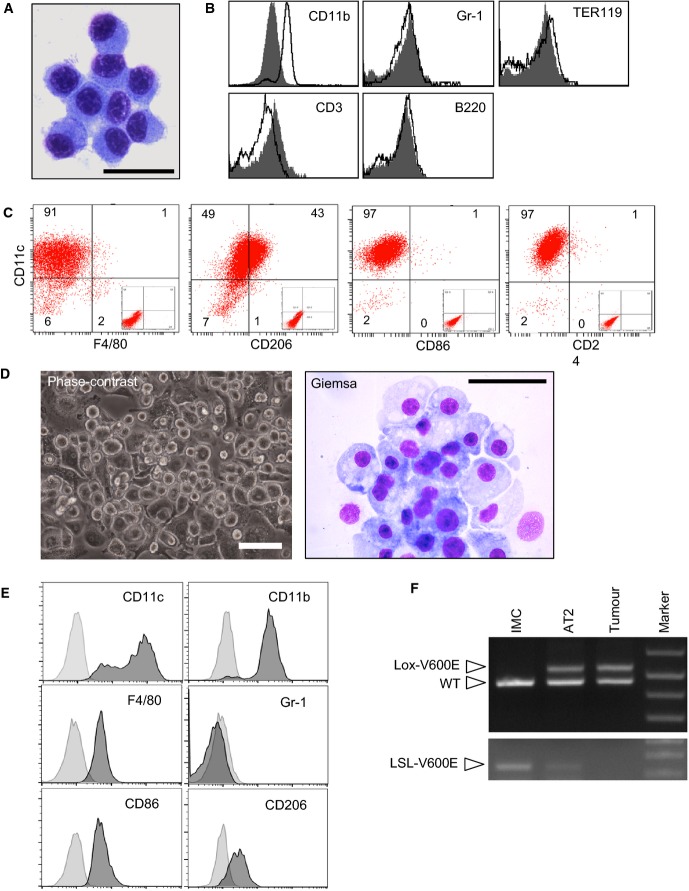
Characterisation of stroma IMCs Giemsa staining of isolated stroma IMCs. These cells show round or oval-shaped nuclei that lack indentations, and have an abundant basophilic cytoplasm without showing prolonged cytoplasmic processes, suggestive of immature myeloid-lineage cells that are morphologically distinct from monocytes/DCs. Scale bar, 25 μm.

We developed a method to separate the stroma cells from the tumour cells (Supplementary Fig S2) and found that the purified stroma cells were identical in morphology to those in histological sections, but rarely displayed indented nuclei or long cytoplasmic processes that are characteristic of monocytes or dendritic cells (DCs), respectively (Figs1C and 2A). Cell surface marker analysis confirmed the expression of a myeloid marker CD11b at low levels in these cells, but other hematopoietic lineage markers, including Gr1, were not detected (Fig2B). The lack of hematopoietic surface marker expression on the isolated stroma cells was not due to enzyme treatment during the isolation procedure since collagenase/DNase treatment of wild-type lung did not perturb the detection of CD11b^high^Gr1^high^ neutrophils, CD11b^+^Gr1^−/low^F4/80^+^ monocytes, CD3^+^ or B220^+^ lymphocytes or TER119^+^ red blood cells (Supplementary Fig S3). These findings exclude the possibility that the stroma cells could belong to myeloid cell types with the CD11b^+^Gr1^+^ phenotype, including neutrophils, inflammatory monocytes (Geissmann *et al*, 2003) and myeloid-derived suppressor cells (MDSCs) (Kusmartsev & Gabrilovich, 2006).

Cell surface marker analysis of IMCs showing lack of common hematopoietic marker expression except for CD11b, which is expressed at low levels (overlaid with isotype control histograms shown in grey).

Cell surface marker analysis (dot plots) of IMCs demonstrating CD11c^+^ IMCs do not express macrophage (F4/80), DC (CD24) or M1-polarisation (CD86) markers, but weakly express the M2 marker CD206. Insets show isotype control staining.

Morphological evaluation of IMCs cultured for 2 weeks without additional cytokines. The cells grown on culture plates were imaged by phase-contrast microscopy (left). Then, the cells were trypsinised and smeared on glass slides for Giemsa staining (right). These cells are intermediate to large sized, round or amoeboid cells with cytoplasmic vacuolation, consistent with a macrophage morphology. Scale bars, 50 μm.

Cell surface marker analysis of cultured IMCs showing differentiation into F4/80^+^ macrophages with CD11b^high^CD11c^+^CD86^+^CD206^low^ surface phenotype (overlaid with isotype control histograms shown in light grey).

PCR detection of *Braf* recombination in purified IMCs, AT2 cells and tumour cell aggregates. Source data are available online for this figure.

In contrast, the stroma cells strongly expressed CD11c, a common marker for lung macrophages and DCs in mice (Misharin *et al*, 2013), whereas the macrophage marker F4/80 was not expressed (Fig2C). Although the CD11b^low^CD11c^+^F4/80^−^ phenotype was somewhat consistent with CD11b^+^ DCs, we think this unlikely as CD24, a marker known to be expressed in circulating DC precursors (O’Keeffe *et al*, 2003) and lung DCs (Misharin *et al*, 2013) in mice, was also undetectable (Fig2C). Instead, these cells weakly expressed CD206, a marker for alternative (M2-type) macrophage activation (Gabrilovich *et al*, 2012), but not CD86, a marker for DC maturation and classical (M1-type) macrophage activation (Gabrilovich *et al*, 2012) (Fig2C). Collectively, the stroma cells did not show typical DC phenotypes but shared some characteristics with M2-polarised macrophages.

To further characterise the stroma cells, we cultured the isolated cells for 2 weeks without adding cytokines. The cells were efficiently maintained in these culture conditions, presumably because of support from autocrine chemo-cytokines and growth factors (see below), and developed larger-sized adherent cells (Fig2D). Morphologically, these cells did not show dendritic cytoplasmic processes, but displayed abundant cytoplasm with numerous vacuoles (Fig2D), consistent with a macrophage rather than DC morphology. Flow cytometry analysis of the cultured stroma cells demonstrated they maintained the CD11c^+^Gr1^−^ phenotype with up-regulation of CD11b and acquisition of F4/80 expression (Fig2E), suggesting macrophage differentiation. Although low-level expression of CD206 was maintained in culture, they also acquired CD86 expression (Fig2E), indicating that macrophage differentiation of the stroma cells in culture was not skewed towards M1 or M2 polarisation. Although we cannot formally exclude the possibility that the stroma myeloid cells are CD117^int^CD11b-CX_3_CR1^+^ macrophage/DC progenitors (MDPs) (Fogg *et al*, 2006), CD11b expression on the stroma myeloid cells (Fig2B) makes this unlikely.

In all, we ascribed these stroma cells the name immature macrophage-lineage cells (IMCs). PCR genotyping of the purified IMCs confirmed that they contained the unrecombined LSL-V600E allele but not the recombined Lox-V600E allele (Fig2F), indicating that they solely express ^WT^BRAF and not ^V600E^BRAF, and thus accumulate as a reactive response to the oncogene-expressing lung tumours.

### Stroma IMCs secrete growth and EMT-promoting factors

**Figure 3:**
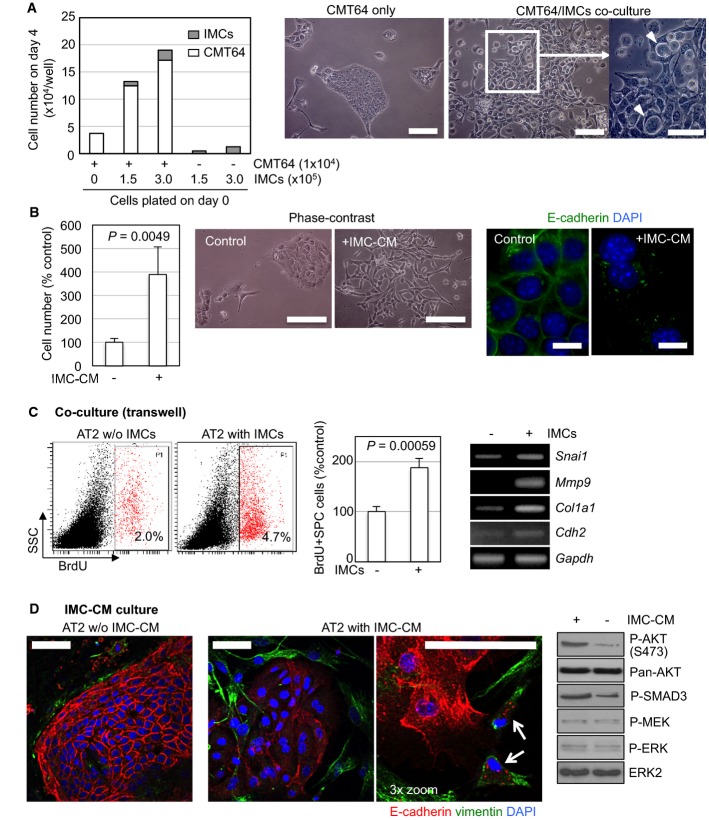
IMCs promote growth and EMT of lung tumour cells CMT64 cells (1 × 10^4^/well in 12-well plates) were co-cultured with 1.5–3 × 10^5^ IMCs for 4 days. Cells were collected by trypsinisation to exclude most of the IMCs since they are resistant to trypsin due to their strong adhesion to the culture plate. Harvested cells were subjected to counting (left). Any contaminating IMCs were separately counted according to their morphological distinction from CMT64 cells. The bar graph (left) shows CMT64 growth (cell counting) data pooled from two independent experiments. Phase-contrast images of CMT64 cells cultured without IMCs (CMT64 only) or co-cultured with IMCs (CMT64/IMC co-culture) are also indicated in the middle and right. A higher magnification image of co-cultured CMT64 cells (right) highlights IMCs migrating into CMT64 cells (arrow heads). Scale bars are 200 μm, except for the high magnification image (right) in which the scale bar is 100 μm.

To explore the biological functions of IMCs, we co-cultured CMT64 mouse lung adenocarcinoma cells (Franks *et al*, 1976) with purified IMCs. IMCs enhanced the growth of these cells in a dose-dependent manner, and promoted fibroblast-like morphological changes suggestive of epithelial–mesenchymal transition (EMT) (Fig3A). Some of the IMCs migrated into CMT64 cell sheets to establish cell-to-cell contacts (Fig3A, right), and therefore, we assessed whether direct cell contacts were required for the phenotype. CMT64 cells cultured with IMC-conditioned media (IMC-CM) recapitulated the phenotypes (Fig3B), indicating that secreted factors rather than cell-to-cell contacts are involved in this response. The IMC-CM also induced down-regulation of E-cadherin expression in the CMT64 cells as demonstrated by immunofluorescence (Fig3B), consistent with an EMT-like response.

(Left) Growth and EMT-like morphological changes of CMT64 cells cultured with 25% IMC-CM. CMT64 cells cultured for 4 days at low-density in DMEM/10%FCS without IMC-CM (-IMC-CM) or with 25% IMC-CM (+IMC-CM) were counted and normalised to the average of control as 100%. The data in the bar chart represent mean + SD of five cultures utilising IMC-CM obtained from three independent sources. (Middle) Phase-contrast images to indicate representative morphologies of CMT64 cells maintained without IMC-CM (control) or cultured with 25% IMC-CM (+IMC-CM) for 2 weeks. (Right) E-cadherin immunofluorescence of CMT64 cells cultured as above. Scale bars are 200 μm (phase contrast) and 10 μm (immunofluorescence).

Co-culture of AT2 cells and autologous IMCs from BVE mice (8- to 10-week-old) for 48 h using the Transwell® culture system. *In vitro* BrdU incorporation (flow cytometry, left and middle) and EMT marker expression (RT–PCR, right) in co-cultured AT2 cells are indicated. Representative flow cytometry plots are indicated on the left, and the bar chart in the middle indicates % BrdU^+^ AT2 cell numbers normalised to control cultures without IMCs (*n* = 4, mean + SD). *Gapdh* serves as a loading control for the RT–PCR on the right.

Morphological alterations and intracellular signalling in AT2 cells cultured with IMC-CM. On the left and middle, primary AT2 cells from 3-week-old BVE mice were cultured for 48 h and then incubated in serum-free IMC-CM (AT2 with IMC-CM) or DMEM (AT2 w/o IMC-CM, serving as a control) for another 48 h, followed by immunostaining for E-cadherin (red) and vimentin (green) and confocal laser scanning microscopy (CLSM) imaging. The 3× zoomed image in the middle right highlights dividing vimentin^+^ cells that show internalised E-cadherin (arrows). Scale bars, 50 μm. On the right, primary AT2 cells from 10-week-old BVE mice were cultured for 48 h, serum starved for 5 h and treated with or without serum-free IMC-CM for 30 min. Phosphorylation of AKT, SMAD3, MEK and ERK were analysed by immunoblotting. Source data are available online for this figure.

Alveolar type-2 (AT2) cells, the major epithelial cell type in ^V600E^BRAF-driven lung tumours (Dankort *et al*, 2007), were also freshly isolated from BVE mice at 10 weeks p.p. using the fractionation method (Supplementary Fig S2). Isolated AT2 cells were validated by the presence of lamellar bodies containing pulmonary surfactants visualised by Papanicolau staining and flow cytometry detection of surfactant protein C (SPC) (Supplementary Fig S2C and D). At this stage, AT2 cells were largely growth-arrested (Fig1B), but the small proliferating pool (∼2%) was detected by *in vitro* BrdU labelling (Fig3C). When these cells were co-cultured with autologous IMCs using a Transwell culture system, there was an approximate doubling of BrdU^+^ proliferating cells, and the expression of EMT markers was also up-regulated (Fig3C). BrdU incorporation into AT2 cells co-cultured with IMCs was higher than those co-cultured with lung fibroblasts (Supplementary Fig S4), indicating that the increased BrdU incorporation in AT2 cells co-cultured with IMCs is unikely to be due to fibroblast contamination.

AT2 cells cultured with IMC-CM displayed a more flattened morphology with down-regulation of membranous E-cadherin, accompanied by vimentin-positive fibroblastic cells surrounding the AT2 cell clusters (Fig3D, middle). Interestingly, mitotic cells expressing vimentin and internalised E-cadherin were also sometimes observed in the IMC-CM cultures (Fig3D, arrows in the middle right microphotograph), suggesting a potential relationship between EMT and the proliferation induced by the IMC-CM. Consistent with the growth/EMT-promoting effects, IMC-CM induced phosphorylation of AKT and SMAD3 in the primary AT2 cells *in vitro,* although no effect on the MEK-ERK pathway was detected (Fig3D).

In order to identify the likely secreted factors involved in this phenotype, we subjected IMC-CM to mass spectrometry analysis. This analysis identified more than 50 secreted proteins including growth factors known to promote cell proliferation and EMT (e.g. TGFβ1, PDGFA, CTGF) (Supplementary Table S1). Secretion of TGFβ and PDGFA was further validated by immunoblotting (Supplementary Fig S5A and B). Secreted proteins previously linked with M2 macrophages and MDSCs (chitinase-3-like 3, arginase-1, S100A9) (Gordon, 2003; Ostrand-Rosenberg & Sinha, 2009) were also identified (Supplementary Table S1). These findings consolidate the M2-polarised nature of the IMCs as well as their potential to promote growth/EMT through paracrine mechanisms.

### CCR1 signalling is required for IMC recruitment and premalignant tumour development

**Figure 4:**
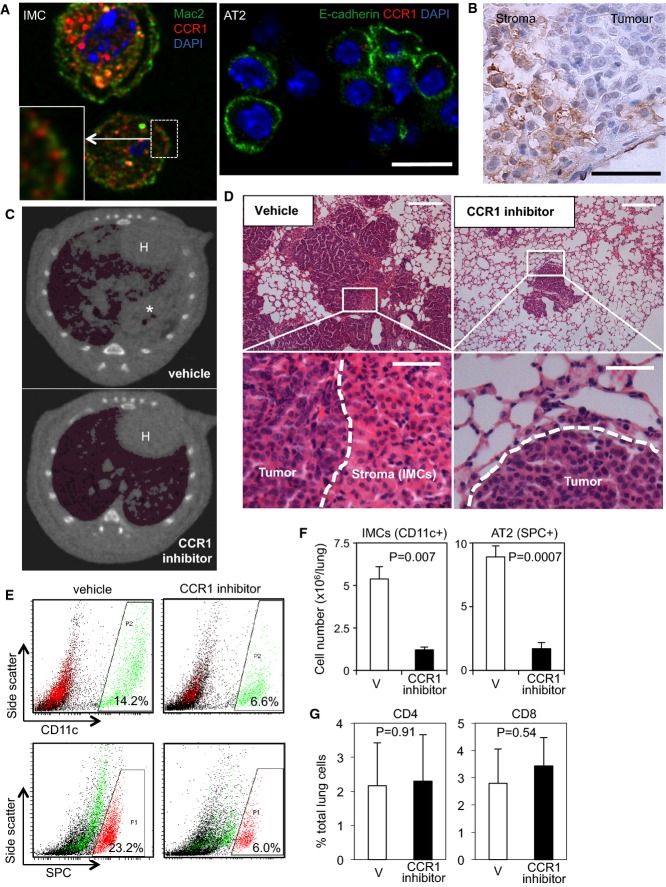
CCR1 inhibition suppresses IMC recruitment and tumour development *in vivo* CCR1 immunofluorescence of primary IMCs (left, co-stained with Mac2) and AT2 cells (right, co-stained with E-cadherin) detected by CLSM. Scale bars, 10 μm. CCR1 staining was mainly detected in cytoplasmic vesicles within IMCs, but some CCR1 fluorescence was also detected at the plasma membrane along with Mac2 (inset in the left microphotograph).

In addition to EMT/growth-promoting factors, CC chemokines CCL6, 7 and 9 were identified in the IMC-CM by mass spectrometry (Supplementary Table S1), and secretion of CCL6 was validated by immunoblotting (Supplementary Fig S5C). CCL6, 7 and 9 are known to be ligands for the chemokine receptor CCR1 (Berahovich *et al*, 2005). We therefore assessed CCR1 expression using immunofluorescence of separated IMCs/AT2 fractions and immunohistochemistry of BVE lung tissue (Fig4A and B). Together, these data confirm predominant expression of CCR1 in IMCs, some of which is localised at the plasma membrane along with Mac2, and very little, if any, expression in ^V600E^BRAF-expressing AT2 cells.

CCR1 immunohistochemistry of lung sections from BVE mice showing CCR1 expression in stroma IMCs. Scale bar, 50 μm.

CT imaging of lungs of AdCre*-*infected *Braf*^+/^^*LSL*^^*−V600E*^ mice treated with vehicle (top) or CCR1 inhibitor (bottom). H: heart; * indicates a tumour region accompanied by atelectasis.

H&E staining of lung sections from vehicle (left) or CCR1 inhibitor (right) treated AdCre*-*infected *Braf*^+/^^*LSL*^^*−V600E*^ mice. Scale bars, 250 μm (top) or 50 μm (bottom).

Flow cytometry analysis of CD11c^+^ (top, green) and SPC^+^ (bottom, red) cells in the lung of AdCre-infected *Braf*^+/^^*LSL*^^*−V600E*^ mice treated with vehicle (left) or CCR1 inhibitor (right).

Lung CD11c^+^ (left) and SPC^+^ (right) cell numbers (per left lobe) were quantitated in AdCre*-*infected *Braf*^+/^^*LSL*^^*−V600E*^ mice treated with CCR1 inhibitor or vehicle (V) (*n* = 3, mean + SD).

% Lung CD4^+^ (left) and CD8^+^ (right) T lymphocytes were quantitated in AdCre*-*infected *Braf*^+/^^*LSL*^^*−V600E*^ mice treated with CCR1 inhibitor or vehicle (V) (*n* = 3, mean + SD). Source data are available online for this figure.

To investigate the role of CCR1 signalling in IMC recruitment, we treated AdCre-infected *Braf*^*+/LSL−V600E*^ mice with the CCR1 inhibitor J-113863 (Amat *et al*, 2006), initiated at 5 weeks following AdCre induction before overt IMC accumulation appeared in the lung. AdCre delivery was used to induce ^V600E^BRAF expression in this particular experiment rather than intercrossing with the CreER™ strain since the compromised health conditions of the BVE mice, often as early as immediately after weaning, did not allow us to perform consecutive i.p. drug injections. CT imaging and histological analysis after 4 weeks of treatment showed not only the suppression of IMC recruitment but decreased tumour burden (Fig4C and D), which was further validated by flow cytometry quantification of CD11c^+^ IMC and SPC^+^ AT2 cells (Fig4E and F) and tumour area quantification of histological sections (Supplementary Fig S6). Of note, the majority of the remaining lung CD11c^+^ cells in inhibitor-treated mice displayed low side-scatter profiles (Fig4E right), suggesting that IMCs with increased intracellular granularity were mostly depleted from the lung by the inhibitor. These data demonstrate an essential role for CCR1 signalling in the recruitment of IMCs and support a role for the IMCs in the *in vivo* maintenance of premalignant lung adenomas in this system.

Single-dose AdCre administration into the mouse lung has been reported to cause inflammatory lymphocyte infiltrations that are persistent for at least 4 weeks in wild-type mice and for up to 24 weeks in KRAS^G12V^ mice (Mainardi *et al*, 2014). Consistent with this, we detected increased T lymphocytes in the lungs of wild-type and *Braf*^*+/LSL−V600E*^ mice at 9–13 weeks after single-dose AdCre administration (Supplementary Fig S7). Since CCR1 blockade is known to inhibit recruitment of not only macrophages but also CD4+ T-cells to nephritic kidneys in an autoimmune disease mouse model (Bignon *et al*, 2014), we were concerned that the phenotype observed in our experiments could be attributed to affects on the T-cell populations rather than IMCs. We therefore analysed the %CD4+ and %CD8a cells but found that these were not significantly affected by CCR1 inhibition in the lungs of *Braf*^*+/LSL−V600E*^*/*AdCre mice (Fig4G). These data suggest that it is the IMCs rather than T-cell populations that are the primary target of the CCR1 inhibitor in our experiments.

### ^V600E^BRAF-driven lung adenomas secrete the cholesterol-binding protein NPC2

**Figure 5:**
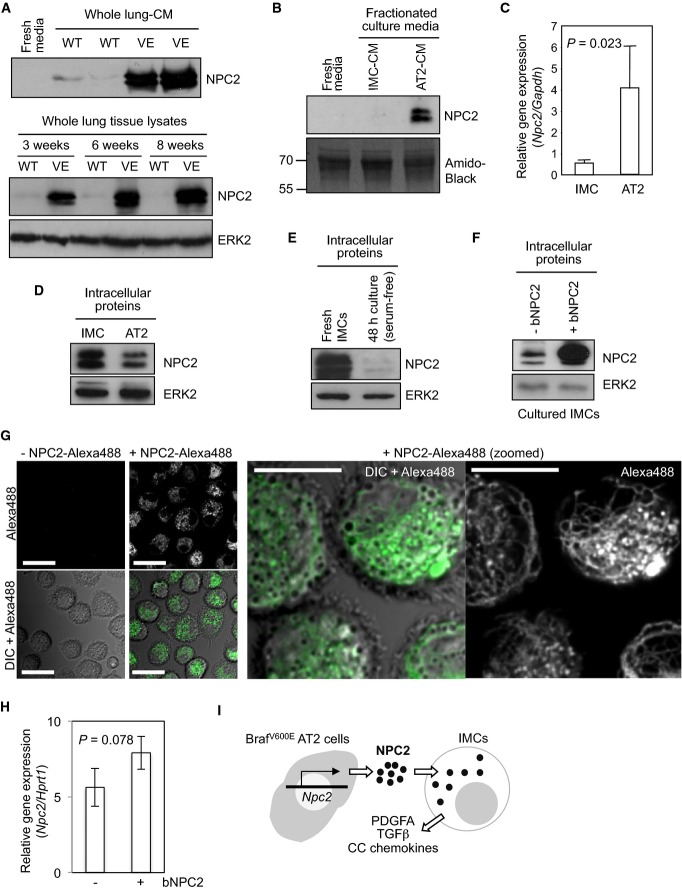
^V600^^E^BRAF-driven lung tumours secrete NPC2 NPC2 immunoblots of concentrated whole lung CM (top) and whole lung lysates (bottom) generated from *Braf*^+/+^*;CreER*^*™*^ (WT) or BVE (VE) mice. Whole lung lysates were obtained from 3-to 8-week-old mice.

We then sought to identify secreted factors from the tumours that may be involved in an interaction with the IMCs. To this end, we generated CM of whole lung tissues (WL-CM) obtained from BVE mice at 6 weeks p.p. (Supplementary Fig S8) when increased tumour burden with relatively modest IMC accumulation is observed (Fig1A). WL-CM was also generated from wild-type mice as a control. 13 proteins uniquely secreted into the BVE WL-CM (but not into the wild-type WL-CM or BVE IMC-CM) were successfully identified by mass spectrometry (Supplementary Table S2). We focused on the cholesterol-binding protein NPC2 since this protein was detected at high levels in the BVE WL-CM by immunoblotting (Fig5A top). Robust induction of NPC2 protein expression in total BVE lung tissue was readily detectable as early as 3 weeks p.p. (Fig5A) before clear OIS responses were observed in the tumours (Fig1B).

NPC2 immunoblots of un-concentrated CM (containing 10% FCS) from fractionated IMC and AT2 cultures. Total protein staining of the membrane by Amido black (bottom) serves as a loading control showing serum albumin at 70 kDa, derived from FCS in the media.

*Npc2* mRNA expression in the purified IMC and AT2 populations as determined by quantitative RT–PCR. Mean + SD (*n* = 3) of *Npc2* expression normalised to *Gapdh* is indicated.

NPC2 immunoblots of cell lysates from freshly isolated IMCs and AT2 cells.

NPC2 immunoblots of cell lysates from freshly isolated or cultured (48 h in serum-free DMEM) IMCs.

NPC2 immunoblots of cell lysates from primary IMCs cultured with or without 50 μg/ml bNPC2 for 48 h.

CLSM imaging of NPC2-Alexa488 taken up by IMCs. (Left) Freshly isolated IMCs were incubated for 2 h with (+NPC2-Alexa488) or without (-NPC2-Alexa488) 82.5 nM NPC2-Alexa488, chased in NPC2-free media for additional 2 h and live imaged by CLSM. Single-colour (top) and DIC-merged (bottom) fluorescence images are indicated. Scale bar, 20 μm. (Right) Zoomed images (DIC-merged and single-colour) from different fields showing vesicular and reticular distribution of incorporated NPC2-Alexa488. Scale bar, 10 μm.

*Npc2* mRNA expression in IMCs cultured for 48 h with or without 50 μg/ml bNPC2 as determined by qRT–PCR. Mean ± SD (*n* = 3) of *Npc2* expression normalised to *Hprt1* is indicated.

The diagram indicates our hypothetical model for the source of NPC2 in IMCs, based on our *ex vivo* observations. Source data are available online for this figure.

To confirm the cell population secreting NPC2, we generated CM from AT2-enriched culture (AT2-CM) (Supplementary Fig S2) and compared NPC2 protein levels in AT2-CM to that in IMC-CM. As shown in Fig5B, NPC2 protein was detected only in AT2-CM by immunoblotting. In line with this, *Npc2* mRNA was expressed at ∼7-fold higher levels in AT2 cells compared to IMCs as assessed using qRT–PCR (Fig5C). However, unexpectedly, intracellular NPC2 protein levels were higher in freshly isolated IMCs than AT2 cells (Fig5D). Interestingly, intracellular NPC2 protein levels in IMCs were dramatically decreased during 48 h of culture (Fig5E), indicating that the relatively low *Npc2* mRNA transcript levels in IMCs (Fig5C) were not sufficient for maintaining intracellular protein levels in freshly isolated IMCs. In contrast, when IMCs were cultured with purified bovine NPC2 (bNPC2), NPC2 protein accumulated in the cells (Fig5F), suggesting active uptake of extracellular NPC2 protein by IMCs. IMC uptake of extracellular NPC2 was further confirmed by culturing freshly isolated IMCs with fluorochrome-conjugated, recombinant NPC2 (Huang *et al*, 2014) (Fig5G), whereas endogenous *Npc2* transcription in IMCs was not significantly affected by bNPC2 treatment (Fig5H). These *ex vivo* findings suggest that NPC2 secreted by the AT2 cells can be incorporated by the IMCs, resulting in the increase of intracellular NPC2 protein in freshly isolated IMCs (hypothetical model is shown in Fig5I).

### NPC2 restrains IMC accumulation

**Figure 6:**
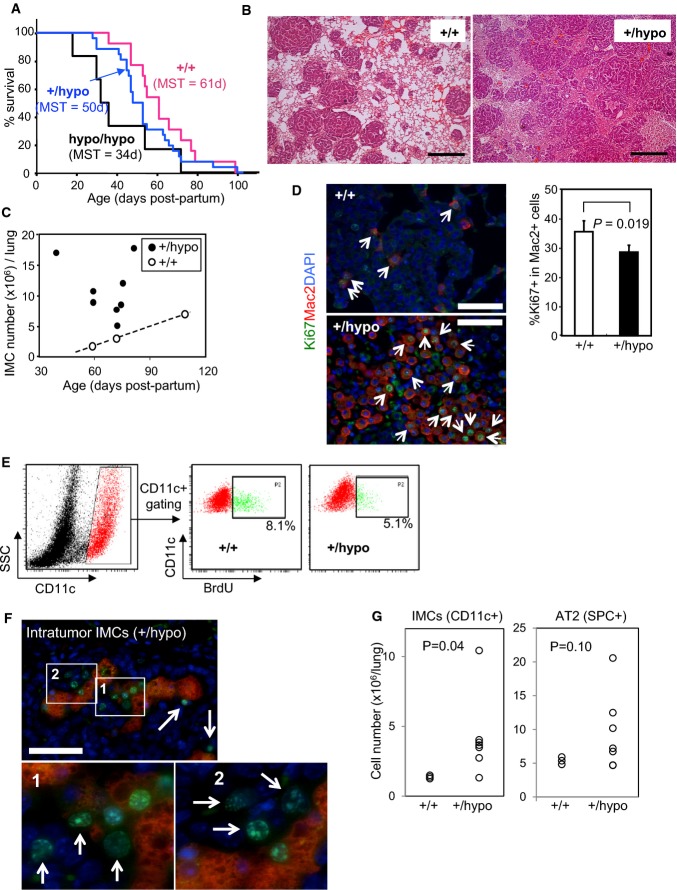
*Npc2* haploinsufficiency causes stroma IMC accumulation Shortened survival of BVE mice following reduction in *Npc2* gene dosage. Genotypes of mice are as follows: +/+ = *Braf*^+/^^*LSL*^^−*V600E*^;*CreER*^+/0^;*Npc2*^+/+^, +/hypo = *Braf*^+/^^*LSL*^^−*V600E*^;*CreER*^+/0^,*Npc2*^+/*hypo*^, hypo/hypo = *Braf*^+/^^*LSL*^^−*V600E*^;*CreER*^+/0^;*Npc2*^*hypo/hypo*^. Median survival time (MST) for each genotype is indicated.

To investigate the biological role of NPC2 in ^V600E^BRAF-driven lung tumourigenesis, we crossed BVE mice with *Npc2* hypomorphic mice (Sleat *et al*, 2004). BVE mice on the *Npc2*^*hypo/hypo*^ background showed a significantly shortened life span compared to *Npc2*^*+/+*^ BVE mice (log-rank *P* = 0.0387), while *Npc2*^*+/hypo*^ BVE mice tended to have shortened survival compared to the *Npc2*^*+/+*^ counterparts (Fig6A), though this was not at a statistically significant level (log-rank *P* = 0.143). We excluded the *Npc2*^*hypo/hypo*^ BVE cohort from further analysis because of the inability to obtain significant numbers of mice due to reduced survival, particularly at later time points when stromal IMC accumulation is evident. We were also concerned that the range of non-respiratory symptoms known to be evident in *Npc2*^*hypo/hypo*^ animals (Sleat *et al*, 2004; Griese *et al*, 2010) would complicate phenotype analysis. Our further studies therefore focused on the BVE *Npc2*^*+/hypo*^ cohort.

Representative H&E staining of lung sections from 5-week-old BVE mice on the *Npc2*^+/+^ (+/+) or *Npc2*^+/*hypo*^ (+/hypo) background is shown. Scale bars, 500 μm.

Stroma IMC quantification per lung in *Npc2*^+/+^ (+/+) or *Npc2*^+/*hypo*^ (+/hypo) BVE mice at different ages (39–109 days p.p.).

Ki67/Mac2 immunofluorescence analysis of *Npc2*^+/+^ (+/+) and *Npc2*^+/*hypo*^ (+/hypo) BVE lung sections at 5 weeks of age. Merged images for Ki67 (green), Mac2 (red) and DAPI (blue) are indicated. Arrows indicate dual positive cells for nuclear Ki67 and Mac2. Scale bars, 50 μm. The bar chart indicates % Ki67 in Mac2^+^ lung stroma cells (mean + SD), obtained from 4 independent *Npc2*^+/+^ and *Npc2*^+/*hypo*^ BVE pairs.

*In vivo* BrdU incorporation of CD11c^+^ IMCs from *Npc2*^+/+^ (+/+) and *Npc2*^+/*hypo*^ (+/hypo) BVE mice are shown. Representative flow cytometry plots of three independent experiments are indicated.

Ki67 (green)/Mac2 (red) dual immunofluorescence staining of lung sections from 5-week-old *Npc2*^+/*hypo*^ BVE mice with nuclear DAPI (blue) staining, focusing on intratumour Mac2^+^ cell clusters. Scale bar, 50 μm. Enlarged images of the boxed areas 1 and 2 highlight Ki67^+^Mac2^−^ cells associated with Mac2^+^ cells (arrows).

Lung SPC^+^/CD11c^+^ cell numbers of *Braf*^+/^^*LSL*^^*−V600E*^*;Npc2*^+/+^ (+/+, *n* = 3) and *Braf*^+/^^*LSL*^^*−V600E*^*;Npc2*^+/*hypo*^ (+/hypo, *n* = 7) mice at 10 weeks post-AdCre infection. Source data are available online for this figure.

Histological analysis at 5 weeks p.p. revealed accelerated accumulation of stroma IMCs in the lung of mice on the *Npc2*^*+/hypo*^ background (Fig6B). Flow cytometry analysis of CD11c+ cells confirmed there was a significant increase in the number of stroma IMCs in the lungs of *Npc2*^*+/hypo*^ BVE mice compared to *Npc2*^*+/+*^ mice throughout their life span (Fig6C). Quantitation of the immunofluorescence co-staining of Mac2 with nuclear Ki67 demonstrated a modest but statistically significant decrease of %Ki67+ in Mac2^+^ cells (Fig6D), suggesting that enhanced recruitment, rather than local proliferation, is the basis for the stroma phenotype. Consistently, the CD11c^+^ population in the *Npc2*^*+/hypo*^ BVE lung also had reduced BrdU incorporation *in vivo* (Fig6E).

Neither AT2 cell number nor their ability to incorporate BrdU was altered in *Npc2*^*+/hypo*^ BVE mice at the pre-senescent stage of tumour development (5 weeks p.p.; Supplementary Fig S9), demonstrating that NPC2 does not regulate the intrinsic growth of the pre-malignant tumour cells, at least at the pre-senescent stage. Furthermore, the data show that pre-senescent AT2 cells are largely insensitive to the growth-promoting effects of IMCs *in vivo*. At this pre-senescent stage of 5 weeks p.p., most tumours are still actively proliferating (Fig1B), but a few of them show the earliest signs of OIS, represented by a loss of nuclear Ki67 staining. Interestingly, at this time point, Mac2^+^ IMCs on the *Npc2*^*+/hypo*^ background were often found co-located with a limited number of Ki67^+^ tumour cells within tumour interstices that had largely lost Ki67 positivity (Fig6F). This possibly indicates that the IMCs have a direct effect on AT2 cells pre-disposed to proliferation at the senescent stage.

Less than 20% of BVE mice on the *Npc2*^*+/hypo*^ background survived until the senescent stage (9–10 weeks p.p.) (Fig6A). Therefore, to accurately evaluate the effect of reduced NPC2 expression at later stages when senescent levels are higher, we utilised *Braf*^*+/LSL−V600E*^*/*AdCre mice on the *Npc2*^*+/hypo*^ background, most of which survived for more than 10 weeks after AdCre delivery. In this setting, decreased NPC2 expression significantly enhanced IMC accumulation and also gave rise to increased AT2 cell number in five out of seven mice compared to the average of *Npc2*^*+/+*^ counterparts, though this difference was not statistically significant due to the variability of the tumour burden of *Npc2*^*+/hypo*^ animals (Fig6G). We also measured tumour burden of *Npc2*^*+/+*^ and *Npc2*^*+/hypo*^ BVE lung by tumour area quantification using histological sections, leading to the same conclusion (Supplementary Fig S6C).

### Exogenous NPC2 decreases cholesterol levels in IMCs

**Figure 7:**
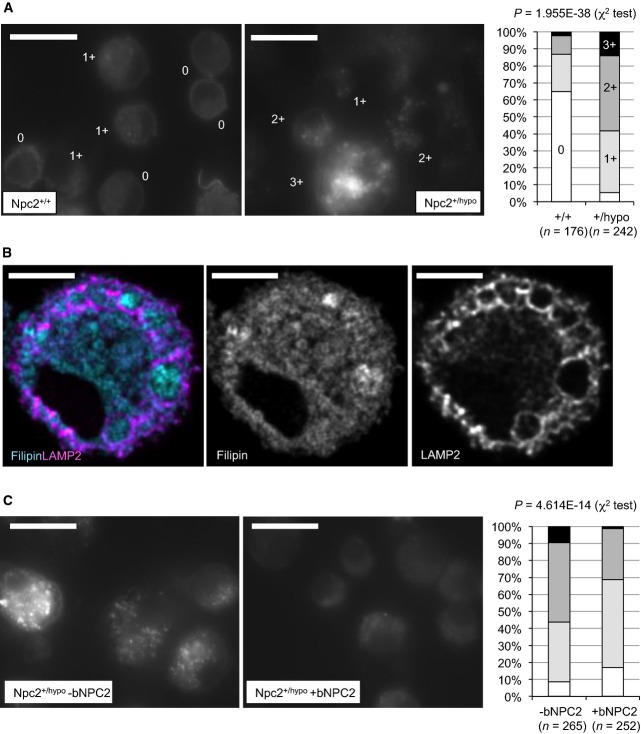
NPC2 reduces intracellular cholesterol accumulation in IMCs Intracellular un-esterified cholesterol distribution as detected by filipin staining in freshly isolated *Npc2*^+/+^ and *Npc2*^+/*hypo*^ IMCs. Scale bars, 25 μm. Filipin-stained cells were graded from 0 to 3+ as indicated in the microphotographs (see Materials and Methods for criteria), and % of each grade in IMCs from *Npc2*^+/+^ (+/+) and *Npc2*^+/*hypo*^ (+/hypo) BVE mice is shown in the bar chart. The data were obtained by analysing 176 *Npc2*^+/+^ and 242 *Npc2*^+/*hypo*^ IMCs from three independent *Npc2*^+/+^ and *Npc2*^+/*hypo*^ pairs.

NPC2 is known to regulate cholesterol trafficking from the late endosomes/lysosomes (LE/Ly) to the cytosol (Subramanian & Balch, 2008), and a homozygous null mutation in *NPC2* causes cholesterol accumulation in LE/Ly in fibroblasts (Naureckiene *et al*, 2000). Therefore, we assessed whether uptake of exogenous NPC2 by IMCs alters un-esterified cholesterol levels. To this end, we utilised *Npc2*^*+/hypo*^ IMCs since accumulation of un-esterified cholesterol is readily detectable by vesicular filipin staining of variable size (Fig7A). In untreated *Npc2*^*+/hypo*^ IMCs, coarse filipin staining was surrounded by LAMP2^+^ reticular structures (Fig7B), but was not associated with LAMP1 or other organelle markers (Supplementary Fig S10), indicating that un-esterified cholesterol accumulation is localised at LAMP2^+^LAMP1^−^ structures that are distinct from LAMP1^+^ lysosomes. Interestingly, the filipin-stained structures partially associated with vertical F-actin-based columns (Supplementary Fig S11) suggestive of podosomes that form constitutively in M2 macrophages (Linder & Wiesner, 2015) and have been functionally linked with lysosomes (Cougoule *et al*, 2005; Tu *et al*, 2008). Following treatment with purified bNPC2, filipin staining in cytoplasmic vesicles was significantly reduced (Fig7C), demonstrating that exogenous NPC2 can decrease free cholesterol accumulated even at non-lysosomal structures in IMCs.

CLSM imaging of fresh *Npc2*^+/*hypo*^ IMCs stained with filipin (cyan) and LAMP2 immunofluorescence (purple). Scale bars, 5 μm. Coarse structures strongly stained with filipin are surrounded by cytoplasmic membranous staining of LAMP2. Single-colour greyscale images are also demonstrated in the middle (filipin) and right (LAMP2).

Intracellular un-esterified cholesterol distribution as detected by filipin staining in *Npc2*^+/*hypo*^ IMCs cultured with or without 50 μg/ml bNPC2 for 48 h. Scale bars, 25 μm. Quantitative data in the bar graph were obtained by analysing 265 bNPC2-untreated (-bNPC2) and 252 bNPC2-treated (+bNPC2) cells from three independent cultures using the method described in (A). Source data are available online for this figure.

### Exogenous NPC2 inhibits CCL6 secretion in IMCs

**Figure 8:**
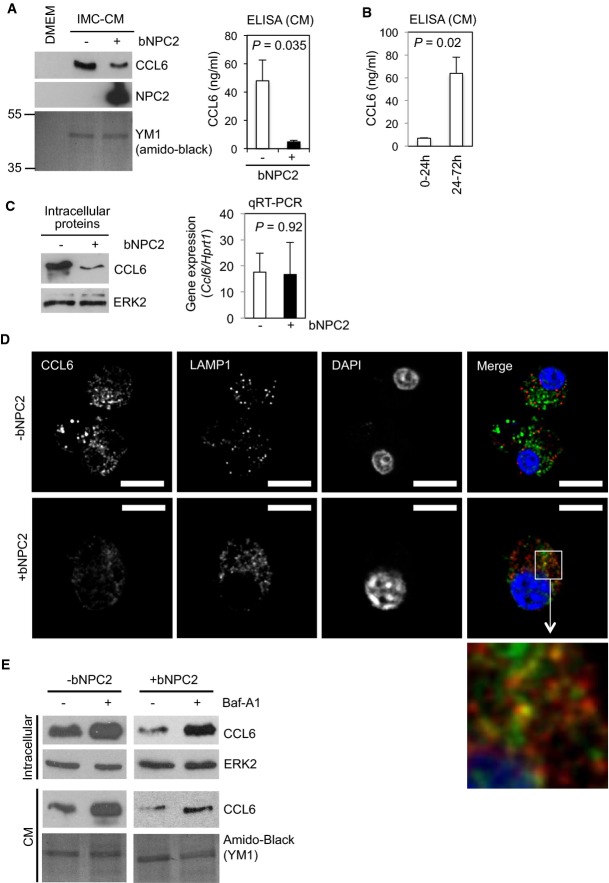
NPC2 inhibits IMC secretion of CCL6 Immunoblot (left) and ELISA (right, *n* = 3) analyses of CCL6 in IMC-CM from primary *Npc2*^+/+^ IMCs cultured with or without 50 μg/ml bNPC2 for 48 h (immunoblots) or 72 h (ELISA). The data in the bar graph (ELISA) represent mean + SD.

Given the previously established function of NPC2 in intracellular cholesterol trafficking, a role in regulation of IMC recruitment is somewhat unusual. However, a link between cholesterol and chemokine secretion in macrophages has been previously documented in atherosclerotic lesions (Moore & Tabas, 2011). As IMC accumulation is dependent on CCR1 signalling (Fig4), we investigated the effect of NPC2 on the secretion of CCR1 ligand CCL6. CCL6 secretion was significantly attenuated when exogenous bNPC2 was added to IMC cultures (Fig8A). Consistent with this, CCL6 secretion levels increased significantly during 24–72 h of IMC culture without exogenous NPC2 (Fig8B) when intracellular NPC2 levels are known to be robustly decreased (Fig5E). Thus, CCL6 secretion inversely correlates with intracellular NPC2 protein levels. *Ccl6* mRNA levels were not affected by NPC2 treatment (Fig8C), suggesting intracellular CCL6 processing at the post-translational level is involved.

Quantification of secreted CCL6 into IMC-CM during the initial 24 h (0–24 h) and subsequent 48 h (24–72 h) of IMC culture by ELISA (*n* = 3, mean + SD).

Immunoblot and qRT–PCR analyses of CCL6 expression in *Npc2*^+/+^ IMCs cultured with or without 50 μg/ml bNPC2 for 48 h. The data in the bar graph (qRT–PCR) represent mean + SD; *n* = 3.

CLSM imaging of CCL6 (green)/LAMP1 (red) immunofluorescence of *Npc2*^+/+^ IMCs cultured with or without 50 μg/ml bNPC2 for 48 h. Scale bars, 10 μm. The right bottom image highlights the boxed area of the merged image of bNPC2-treated IMCs showing partial co-localisation of LAMP1 and CCL6.

Immunoblot analysis of intracellular and secreted (CM) CCL6 in bafilomycin-treated *Npc2*^+/+^ IMC culture. IMCs without (left) or with (right) 50 μg/ml bNPC2 pre-loading for 30 min were chased in serum-free DMEM for 3 h and then treated with 200 nM bafilomycin A1 for 24 h. Source data are available online for this figure.

To examine the mechanistic basis for this phenotype, we investigated CCL6 subcellular localisation in cultured IMCs. When IMCs were cultured without bNPC2, CCL6 showed vesicular distribution, mostly devoid of LAMP1 staining. Following bNPC2 treatment, CCL6/LAMP1 co-localisation was detected at perinuclear regions and cytoplasmic vesicles (Fig8D). The perinuclear CCL6 staining was confirmed to be not only within Golgi-related structures stained with a Golgi-cisternae marker giantin or a trans-Golgi network marker TGN46, but also at non-Golgi (giantin/TGN46^−^) vesicular structures (Supplementary Fig S12) that may include perinuclear lysosomes. These findings suggest that CCL6 could be targeted to the lysosomes by exogenous NPC2. To validate the functional contribution of the lysosome to decreased CCL6 protein levels, we treated IMCs with bafilomycin A1 (Baf-A1) to interfere with lysosomal functions by inhibiting vesicular acidification. As shown in Fig8E, Baf-A1 treatment rescued intracellular CCL6 protein levels in IMCs treated with exogenous NPC2, indicating that the NPC2-induced reduction in intracellular CCL6 protein is attributable, at least in part, to lysosomal degradation. CCL6/LAMP1 dual staining of bNPC2-loaded, Baf-A1-treated IMCs also showed co-localisation of CCL6 and LAMP1 more clearly (Supplementary Fig S13) than those without Baf-A1 treatment (Fig8D), further supporting the role of lysosomal degradation in CCL6 reduction. Interestingly, vesicular CCL6 staining in the periphery of the cytoplasm did not co-localise with LAMP1 in bNPC2-loaded, Baf-A1-treated IMCs (Supplementary Fig S13), implying that vesicular acidification may be required for CCL6 to be fully transferred to the lysosomes. In contrast to the intracellular protein levels, CCL6 secretion into the CM was not rescued by Baf-A1 (Fig8E), suggesting that CCL6 accumulating in the lysosomes and the periphery of the cytoplasm by this treatment is not effectively exocytosed.

## Discussion

NPC2 is a small glycoprotein that was initially identified as a secretory protein in epididymal fluid and later characterised as the product of the second defective gene in Niemann-Pick type C (NPC) disease. Because of the early death of NPC patients suffering from severe neurodegeneration (Vanier, 2010), predisposition to cancer has been difficult to assess although several sporadic cases have been reported to develop malignancies (Birch *et al*, 2003). Intracellular NPC2 protein is known to regulate cholesterol transport from the LE/Ly to the cytosol, and the incorporated extracellular NPC2 protein is known to impart functional properties to NPC2-deficient fibroblasts in the same manner as the endogenously expressed protein (Naureckiene *et al*, 2000). Although such *in vitro* studies suggest that secreted NPC2 exerts its biological functions through autocrine/paracrine mechanisms, *in vivo* evidence for this is lacking. In this study, we have found that ^V600E^BRAF-driven early-stage lung tumours abundantly secrete NPC2 protein and that the secreted NPC2 protein is incorporated by stromal IMCs, suppressing CCL6 secretion by these cells. This has important consequences since accumulation of IMCs relies on signals through the CCL6 receptor, CCR1 (Fig4). Thus, NPC2 secreted by pre-malignant lung tumours interferes with the tumour microenvironment through paracrine mechanisms.

Tumour-associated macrophages (TAMs) are the major myeloid cell type in the tumour microenvironment (Grivennikov *et al*, 2010), and most TAMs are considered to be M2-like macrophages (Biswas & Mantovani, 2010). The IMCs described here are morphologically distinct from TAMs, but their CD11b^low^CD11c^+^ surface phenotype is similar to TAMs in a mammary tumour model recently reported (Franklin *et al*, 2014). IMCs in our model also secrete M2 marker proteins including YM1, arginase-1, MRC1 (soluble CD206) and legumain (Luo, 2006; Biswas & Mantovani, 2010; Murray & Wynn, 2011) (Supplementary Table S1) and promote tumour cell growth *in vitro* and *ex vivo* (Fig3). Furthermore, the *in vivo* targeting of IMCs by the CCR1 inhibitor effectively suppressed tumour progression *in vivo* (Fig4) in a similar way to the targeting of TAMs in the oncogenic KRAS-induced lung adenocarcinoma model (Cortez-Retamozo *et al*, 2012). This demonstrates for the first time that lung cancer progression relies on tumour-associated macrophage-lineage cells not only at advanced stages but also at pre-malignant stages. Interestingly, TAMs in the KRAS model were derived from the spleen through CCR2 signalling (Cortez-Retamozo *et al*, 2012), whereas the recruitment of IMCs in our model is dependent on CCR1 signalling (Fig4). Since CCR1 is essential for myeloid progenitor mobilisation (Gao *et al*, 1997), it is plausible that myeloid progenitors are redistributed to the lung by CCR1 signalling and therein serve as precursors for IMCs.

Using *ex vivo* studies, we have shown that IMCs have pro-tumourigenic properties by promoting the growth and EMT of AT2 cells in culture (Fig3) and abrogation of IMCs by the CCR1 inhibitor provides clear evidence for the pro-tumourigenic/tumour-supportive functions of IMCs (Fig4). However, the *Npc2*^*+/hypo*^ BVE mice demonstrate IMC amplification by ∼2- to 6-fold without significant enhancement of tumour progression or AT2 growth (Fig6). We believe this latter observation does not challenge the pro-tumourigenic/tumour-supportive role of IMCs but, rather, suggests this level of amplification of IMCs is not sufficient to give a dramatic *in vivo* response and/or the tumour response and IMC quantity do not show a linear correlation *in vivo*. Altogether, our data show a clear role for the IMCs in tumour maintenance, but a function of these cells in further tumour progression and escape from OIS is currently not clear from our analysis.

The tumour cells were identified to secrete a number of factors, although senescence-associated secretory proteins (SASPs) previously identified (Kuilman & Peeper, 2009) were not detected within our data set (Supplementary Table S2). The most likely explanation for this is that we analysed lung tissue containing tumours that were not fully senesced at 6 weeks of age. We focused on NPC2 since this cholesterol-binding protein has not previously been characterised in the context of lung tumourigenesis. Although NPC2 is secreted at high levels by ^V600E^BRAF-expressing AT2 cells, IMC accumulation is still evident, which would suggest that the protein is not the only chemo-attractant factor regulating IMC recruitment. We speculate that IMC recruitment could be initiated by factors derived from non-IMC cells before being robustly facilitated by autocrine CCL6. Secretion of such factors may be regulated independently of NPC2.

NPC2 has been previously reported to be abundantly secreted by human and mouse lung adenocarcinoma cells (Taguchi *et al*, 2011). Although this previous study detected NPC2 secretion from human lung adenocarcinoma cell lines (Taguchi *et al*, 2011), it remains unclear if NPC2 secretion levels could be altered by the oncogenic mutation status and/or disease progression. Since our data suggest that NPC2 could potentially be a tumour suppressor that functions at pre-malignant stages, quantitative evaluation of NPC2 secretion by lung tumours at different developmental stages will be important. To this end, oncogenic KRAS-driven lung tumour models will be useful since the progression of early precursor lesions into malignant adenocarcinoma can be tracked in these models (Jackson *et al*, 2001; Mainardi *et al*, 2014). A comparison between KRAS and BRAF models may also provide insight into whether different driver oncogenes alter NPC2 expression/secretion and potentially affect the microenvironment and malignant progression of early lung lesions. These experiments are currently underway in our laboratory.

Based on our present study, we hypothesise that human lung AAH lesions, the precursors for adenocarcinomas, may respond to chemopreventive interventions targeting the microenvironment with CCR1 inhibitors, NPC2 protein itself or chemicals that up-regulate NPC2 expression. CCR1 inhibitors have already entered clinical trials for the treatment of autoimmune diseases such as rheumatoid arthritis (Tak *et al*, 2013) and so have the potential to be tested in a lung cancer setting. A number of new therapeutic strategies against NPC disease are being experimentally investigated, including histone deacetylase inhibitors that increase NPC protein expression (Pipalia *et al*, 2011). Such chemical compounds may also effectively target tumour-associated macrophage-lineage cells in early-stage precursors for lung adenocarcinomas, through increasing NPC protein expression. Further investigations and translational studies will be needed to explore the clinical relevance of our animal study and the chemopreventive potentials of chemical compounds targeting CCR1 and the NPC pathway.

## Materials and Methods

### Animals

All animal experiments were performed under UK Home Office License authority. *Braf*^*+/LSL−V600E*^ (Mercer *et al*, 2005), *CCAGCreER*^*™*^ (Hayashi & McMahon, 2002) and *Npc2* hypomorph (Sleat *et al*, 2004) mice were genotyped as reported. Male and female mice were randomly selected for each experiment. Unless otherwise stated, all experiments on BVE mice utilised animals of 9–10 weeks of age. Nasal delivery of AdCre was performed as described (Dankort *et al*, 2007) on mice at 8–10 weeks of age. 8 mg/kg of CCR1 inhibitor J-113863 (Tocris Bioscience) dissolved in phosphate-buffered saline (PBS) with 5% DMSO (Sigma) and 5% Cremophor™EL (Sigma) was injected intraperitoneally 5 days/week for 4 weeks starting at 5 weeks after AdCre administration. Lung tissues were processed for H&E staining and immunohistochemistry (IHC) as described (Mercer *et al*, 2005), except for CCR1 IHC for which the Novolink™ Polymer Detection System (Leica Biosystems) was used according to manufacturer’s instructions. Antibodies used for IHC are described in Supplementary Methods. μCT imaging of mice was performed using a Quantum FX μCT system (PerkinElmer).

### Cell culture

All cultures were performed in DMEM with 10% FCS, unless otherwise described. CMT64 cells were obtained from Cell Service at Cancer Research UK. Bovine NPC2 (bNPC2) was added to cultures at 50 μg/ml. Co-cultures of CMT64/IMCs were performed in 12-well plates with 1 × 10^4^ CMT64 cells and 1.5–3 × 10^5^ IMCs. For co-culture of primary AT2 cells with IMC/lung fibroblasts using 6-well Transwell® plates (0.4 μm pore, Corning), 2 × 10^6^ IMCs or 6 × 10^5^ lung fibroblasts were plated into bottom wells, and 3 × 10^6^ AT2 cells were added in transwell inserts. For NPC2 uptake experiments, NPC2-Alexa488 was added to IMCs at 82.5 nM. For bafilomycin A1 (Baf-A1, Sigma), IMCs pre-loaded with bNPC2 (50 μg/ml) for 30 min were chased in serum-free DMEM for 3 h and then incubated with 200 nM Baf-A1 for 24 h.

### Flow cytometry

Cell surface markers were analysed by flow cytometry as described (Kamata *et al*, 2010). Antibodies used for flow cytometry are described in Supplementary Methods. For *in vitro* BrdU incorporation, cells labelled with 10 μM BrdU for 24 h were analysed using an APC BrdU Flow Kit (BD Biosciences). *In vivo* BrdU incorporation was performed as described (Kamata *et al*, 2010). For intracellular SPC staining, primary lung cells were fixed/permeabilised using a BD Cytofix/Cytoperm kit (BD Biosciences), and stained for SPC for 1 h at 37°C, followed by incubation with AlexaFluor® 488-conjugated anti-rabbit IgG Fab (Invitrogen, 1:2,000) for 20 min at room temperature.

### Immunofluorescence and filipin staining

Cells cultured on coverslips were fixed in 4% (w/v) paraformaldehyde/PBS for 10 min, permeabilised in 0.4% (v/v) Triton-X/PBS for 10 min and incubated in blocking buffer (PBS containing 5% (w/v) bovine serum albumin) for 30 min. Samples for biotinylated primary antibody staining were further blocked for endogenous biotin using streptavidin solution (Vector Laboratories) for 15 min followed by incubation with 0.01% (w/v) biotin (Sigma)/PBS. Lung tissues were processed as described (Mercer *et al*, 2005). Primary antibody staining was performed in blocking buffer for 30 min at room temperature or for 16 h at 4°C, followed by secondary staining in blocking buffer containing AlexaFluor® (488 or 568)-conjugated anti-rabbit and/or anti-mouse IgG Fab, and/or AlexaFluor® 488-conjugated streptavidin (all Invitrogen, 1:2,000). Primary antibodies used for immunofluorescence are described in Supplementary Methods. For intracellular un-esterified cholesterol staining, cells were stained with filipin as described (Kruth *et al*, 1986). Filipin-stained cells were graded as follows: (0) no clear vesicular filipin staining; (1+) less than 10 small vesicles stained at higher intensities than cytosol; (2+) more than 10 small filipin^+^ vesicles and/or one or more large filipin^+^ vesicle(s); and (3+) diffuse distribution of filipin^+^ vesicles throughout cytoplasm. Widefield fluorescence microscopy images were obtained using a Nikon TE300 inverted microscope equipped with Hamamatsu ORCA-R^2^ digital camera and an X-cite120 fluorescence illumination system, and processed using Volocity (Improvision) and ImageJ (NIH) software. Confocal laser scanning microscopy (CLSM) images were obtained using an Olympus FV1000 confocal laser scanning system on an inverted IX81 motorised microscope equipped with UPlanSApo 60x/1.35NA objective (Olympus), and deconvoluted using Huygens Essential software (Scientific Volume Imaging).

### Protein analysis

Protein lysates were prepared as previously described (Kamata *et al*, 2010). For quantitative comparison of secreted proteins in CM, 2 × 10^6^ cells were cultured in a 6-cm dish in 2 ml DMEM for 72 h. CM was mixed with 4× SDS sample buffer (250 mM Tris–HCl pH 6.8, 8% (w/v) SDS, 40% (v/v) glycerol, 20% (v/v) β-mercaptoethanol) and boiled for 5 min. Western blots were performed by loading 20–40 μg cellular lysate or 18–25 μl CM sample per lane, as described (Kamata *et al*, 2010). Equal loading/transfer of CM samples was confirmed by Amido black staining of the membranes showing proteins around 70 kDa (containing serum albumin) for serum-containing samples, or around 45 kDa and 12 kDa (containing M2-marker YM1 and macrophage-lineage marker lysozyme C-2, respectively) for serum-free IMC-CM. Primary antibodies used for immunoblotting are described in Supplementary Methods. CM diluted at 1:100 was also subjected to CCL6 quantitation using a RayBio® Mouse CCL6 ELISA kit (RayBiotech) according to the manufacturer’s instructions.

### RT–PCR

RNA was prepared as described (Noble *et al*, 2008). 0.5 μg RNA was reverse transcribed using Superscript III (Invitrogen) according to the manufacturer’s instructions, and 1 μl of the product was subjected to PCR using ReddyMix™ (Thermo Scientific). Quantitative RT–PCR was performed as described (Noble *et al*, 2008). Primers used are described in Supplementary Methods.

### Statistics

Comparison between any two groups was performed by unpaired Welch’s *t*-test with assuming normal distribution of biological data. Bonferroni correction was applied for data analysis that needed multiple comparisons. Log-rank tests were used to evaluate differences in survival between two groups. Chi-square tests were used to evaluate differences in the frequency of the cells accumulated with un-esterified cholesterol vesicles.

## Supporting Information

**Source Data for Supplementary Figure S9**

**Review Process File**
