# Supplementary material for: The cholesterol-binding protein NPC2 restrains recruitment of stromal macrophage-lineage cells to early-stage lung tumours
Source: EMBO Mol Med. 2015 Jul 16;7(9):1119–37. doi: 10.15252/emmm.201404838 (PMC4568947; doi:10.15252/emmm.201404838)
Supplement: Supplementary file 1 [file emmm0007-1119-sd1.pdf]

SUPPLEMENTARY INFORMATION

CONTENTS

1. SUPPLEMENTARY METHODS
2. SUPPLEMENTARY TABLES
3. SUPPLEMENTARY FIGURE LEGENDS
4. SUPPLEMENTARY FIGURES

SUPPLEMENTARY METHODS

Senescence-associated β-galactosidase staining

Harvested BVE lung tissues were embedded into OCT compound, frozen in a hexane/dry ice bath, and sectioned at 6-8μm thickness. The sections were immediately fixed in 2%PFA/PBS for 10min, and stained using Senescence β-Galactosidase Staining Kit (Cell Signaling) according to the manufacturer's instructions, followed by counterstaining with Hematoxylin.

Antibodies

Antibodies used for immunohistochemistry were: Ki67 (Thermo Scientific #MA1-90584, 1:200), MAC2 (Cedarlane #CL8942AP, 1:500), $p21^{CIP1}$ (Santa Cruz #sc-397, 1:1000), CCR1 (Abcam #ab1681, 1:1500). Antibodies used for flow cytometry were: CD11b (clone M1/70), Gr-1 (clone RB6-8C5), CD3 (clone 17A2), CD8a (clone 53-6.7), B220 (clone RA3-6B2), TER119, CD24 (clone M1/69) purchased from BD Biosciences, mouse CD11c (clone N418), F4/80 (clone BM8), CD206 (clone C068C2), CD86 (clone GL-1), CD4 (clone GK1.5) purchased from BioLegend, and anti-SPC antibody (SantaCruz #sc-13979). Primary antibodies used for immunofluorescence were: E-cadherin (BD Biosciences #610182, 1:2000), CCR1 (Abcam #ab1681, 1:200), MAC2 (Cedarlane #CL8942AP, 1:500), Ki67 (Thermo Scientific #MA1-90584, 1:200), LAMP1 (Abcam #ab13523, 1:200), LAMP2 (Abcam #ab37024, 1:200), EEA1 (Cell signaling #2411, 1:100), Giantin (Abcam #ab24586, 1:200), TGN46 (Abcam #ab16059, 1:200), VAMP3 (Synaptic Systems #104 103, 1:200) and CCL6 (Abcam #ab83134, 1:200). Primary antibodies used for immunoblotting were: $p21^{CIP1}$ (Santa Cruz #sc-397), $p19^{ARF}$ (Abcam #ab80), $p16^{INK4}$ (Santa Cruz #sc-1207), γH2AX (Cell Signaling #9718), phospho-Akt (Ser473) (Cell Signaling #4060), pan-Akt (Cell Signaling #4691), phospho-SMAD3 (Ser423/425) (Abcam #ab52903), phospho-MEK (Ser217/221) (Cell Signaling #9154), Thr202/Tyr204 phospho-p44/42 ERK1/2 (Cell Signaling #9101), ERK2 (Santa Cruz #sc-1647), TGF-β (Cell Signaling #3709), PDGFA (Millipore #07-1436), NPC2 (a gift from Peter Lobel), and CCL6 (Abcam #ab83134).

Protein lysate preparation for immunoblot analysis of senescence markers

Whole lung lysates from BVE lung tissues and whole cell lysates from freshly purified IMCs and AT2 cells were prepared by directly solving samples in SDS sample buffer (2%SDS, 62.5mM Tris-HCl pH6.8, 10% glycerol, and 5%β-mercaptoethnol).

Isolation of lung tumour and stroma cells

Harvested lungs were minced and incubated in Buffer-A [RPMI1640 (Invitrogen) with 5% FCS (Sera Laboratories International), 1 mg/ml collagenase (Sigma), and 20 mg/ml DNase (Sigma)] for 30 min at 37°C. After gentle pipetting, digested tissues were filtered through a 100 mm nylon mesh, overlaid onto 50% Histopaque-1119 (Sigma) and centrifuged at 600 x g for 20 min. The resultant pellet was treated with red blood cell lysis buffer, re-suspended in DMEM (Invitrogen) with 10% FCS, and incubated for 30 min to obtain adherent stroma cells enriched for $CD11c^{+}CD11b^{low}$ IMCs. For lung epithelial cell isolation, undigested tissues after 30 min incubation in Buffer-A above were washed 3 times in PBS and incubated in Buffer-B [RPMI1640 with 1 mg/ml elastase (Worthington Biochemical) and 20 mg/ml DNase] for 90 min at 37°C. After vigorous pipetting, digested tissues were filtered through a 100 mm nylon mesh, mixed with FCS to inactivate elastase, and incubated for 30min on culture plates. Non-adherent cells after 30 min incubation were overlaid onto 40% Histopaque-1119 and centrifuged at 600 x g for 20 min. The resultant pellet was treated with red blood cell lysis buffer, re-suspended in DMEM with 10% FCS, and incubated again for 1 hr on culture plates. Non-adherent cells at this stage were enriched for AT2 cells. To quantify IMCs and AT2 cells in the lungs, whole lung tissues were completely digested in Buffer-A for 3 hrs, treated with red blood cell lysis buffer, and counted using a hemocytometer. Total lung cell number was multiplied by %CD11c/SpC+ cells to calculate IMC and AT2 cell numbers.

Modified Papanicolau staining

Freshly isolated AT2 cells were smeared on glass slides, air-dried, and stained as previously described (Dobbs, 1990).

Isolation and characterisation of lung fibroblasts from BVE mice

Total lung cells obtained from BVE lung by 3hr collagenase/DNase treatment were re-suspended at $5x10^{6}$/ml in DMEM/10%FCS, plated onto 12-well plates at 1ml/well and incubated for 5-7 days until the culture became confluent. The culture was passaged onto 6-well plates by trypsinization. When the culture became confluent again at 3-5 days after re-

plating, the cells were counted using a hemocytometer, and subjected to serial passage culture according to the 3T3 protocol. Since IMCs strongly adhere to the culture plate and are not detached by trypsinization, and AT2 cells do not survive after trypsinization, only fibroblasts grew under this culture condition, which allowed us to obtain a highly enriched fibroblast population until passage 5. For transwell co-culture, lung fibroblasts were plated into the bottom wells at 6×10^5/well before inserts containing isolated AT2 cells were placed on top. Under this condition, lung fibroblasts in the bottom became confluent after 48h of co-culture without detectable contamination of other cell types, as confirmed by phase-contrast microscopic observation after removal of the inserts. For immunoblot analysis of proteins secreted by lung fibroblasts, confluent lung fibroblasts in 10cm plates were cultured for 72h in 5ml serum-free DMEM to collect CM. The CM was concentrated using Amicon® Ultra-4 Centrifugal Filter Units (Millipore), and final volumes of the concentrated CM was adjusted by protein quantification of cell lysates obtained from the cells used to collect the CM. 20-30μl concentrated CM was analysed by immunoblotting along with IMC-CM adjusted in an identical manner.

Tumour area quantification

Sequential images covering the whole lung (right lobe) of histological sections stained with H&E were obtained using a Nikon Eclipse Ti microscope, equipped with Nikon CFI Plan Fluor Ph1 10x/0.3NA objective and Andor iXon^{EM}+ EMCCD (DU-885) camera, with automatic focus correction (Perfect Focus) system. The images acquired through RGB filter sets were merged and processed using Nikon NIS-Elements software to create whole lung images. Total lung area and tumour area including stroma components were quantified using Image J, and %tumour area (tumour area/total lung area x 100) was calculated.

Preparation of conditioned media

To collect whole lung conditioned media (WL-CM), chopped lung tissue was passed through a 70mm nylon mesh, and cultured in serum-free DMEM for 24 hrs. To collect conditioned media from primary culture of IMCs (IMC-CM), purified IMCs were plated at up to 2×10^6/ml in DMEM and maintained for 72 hrs. Following removal of debris, culture supernatants were concentrated using Amicon® Ultra-4 Centrifugal Filter Units (Millipore). The volume of the concentrated WL-CM was adjusted in proportion to the weight of the initial lung tissue. Secreted proteins within the WL-CM and IMC-CM were identified by mass spectrometry as described below.

Preparation of NPC2

Bovine NPC2 (bNPC2) was purified from the whey fraction of whole cow milk by the two-step ion exchange chromatography previously described (Larsen *et al*, 1997) with the following modifications: (1) the pH of CH_3COONH_4 solution was adjusted to 7.0 for the first DEAE-sepharose chromatography; (2) flow-through from the first chromatography was collected, adjusted to pH 5.0, and applied to the second CM-sepharose chromatography; (3) proteins bound to the CM-sepharose were eluted with a two-step gradient (10-50mM for an initial 1 column volume and 50-200mM for subsequent 25 column volumes) of CH_3COONH_4 (pH 5.0). bNPC2 was eluted as a first peak in the second gradient. Fractions containing bNPC2 confirmed by immunoblotting were pooled, dialysed against PBS, and concentrated using Amicon® Ultra-4 Centrifugal Filter Units (Millipore). The purity of bNPC2 was confirmed by Coomassie blue-staining on SDS-PAGE gels and mass spectrometry. The concentration of bNPC2 in the final solution was quantified by UV absorbance at 280nm. Alexa488-conjugated, recombinant human NPC2 protein (NPC2-Alexa488) (ref) was kindly provided by Peter Lobel,

Mass spectrometry

Concentrated CM samples were resolved on SDS-PAGE gels and stained with Coomassie Blue. Bands of interest were excised and destained in 200 mM ammonium bicarbonate in 20% acetonitrile, followed by reduction in 10 mM dithiothreitol, alkylation in 100 mM iodoacetamide and trypsin digestion using an automated robot (Multiprobe II Plus EX, Perkin Elmer). In-gel digested peptides were analyzed by liquid chromatography coupled with tandem mass spectrometry (LC-MS/MS) using an RSLCnano HPLC system (Dionex) and an LTQ-Orbitrap-Velos mass spectrometer (Thermo Scientific). The raw data file obtained from each LC-MS/MS acquisition was searched using Mascot (version 2.2.04, Matrix Science Ltd.) against the UniProtKB/Swissprot4 database, and further processed using Scaffold5 (version 3.00.08, Proteome Software). The threshold for protein identification probability was set as 75%, according to the criteria based on the manufacturer's definitions.

RT-PCR primers

Primers used for RT-PCR were: 5'-CACACGCTGCCTTGTGTCT-3' and 5'-GGTCAGCAAAAGCACGGTT-3' for *Snai1*; 5'-TGTGCGACCACATCGAACTT-3' and 5'-GGCACGCTGGAATGATCTAA-3' for *Mmp9*; 5'-CATGTTCAGCTTTGTGGACCT-3' and 5'-

GCAGCTGACTTCAGGGATGT-3' for *Col1a1*; 5'- TGCCACACAGATCCCATGTA-3' and 5'-GGTTCCCCTCCTGCTGATAA-3' for *Ccl6*; 5'-GTGGCAGCTGGTCTGGATCGAGAGAAAG-3' and 5'-TCAGCATGGTACCTGCGTGGAGGCCTTC-3' for *Cdh2*; 5'-CCTGAATAAGCTTCCGGTGA-3' and 5'-AGTTTCCATTCCACCACCAG-3' for *Npc2*; and 5'-AGGTCGGTGTGAACGGATTTG-3' and 5'-TGTAGACCATGTAGTTGAGGTCA-3' for *Gapdh*.

Sample size determination

In most experiments, we first performed small-size experiments (n=3-5) which are sufficient for detecting statistically significant differences with large effect sizes by t-tests. When the first experiments showed no significant differences but suggested the possibility to show significant differences with relatively small effect sizes, we performed additional experiments to increasing the samples sizes for statistical analyses.

Reference

Dobbs LG (1990) Isolation and culture of alveolar type II cells. *Am J Physiol* 258: L134-147

Larsen LB, Ravn P, Boisen A, Berglund L, Petersen TE (1997) Primary structure of EPV20, a secretory glycoprotein containing a previously uncharacterized type of domain. *Eur J Biochem* 243: 437–441

SUPPLEMENTARY TABLES

Table S1

Secreted proteins identified in conditioned media generated from the culture of purified IMCs

**Protein name**	**Symbol**	**NCBI Accession no.**
**Proteases** (18 proteins)		
Aminopeptidase N (CD13)	AMPN	NP_032512
Cathepsin B	CTSB	NP_031824
Cathepsin D	CTSD	NP_034113
Cathepsin H	CTSH	NP_031827
Cathepsin K	CTSK	NP_031828
Cathepsin L1	CTSL1	NP_034114
Cathepsin S	CTSS	NP_001254624
Cathepsin Z	CTSZ	NP_071720
Dipeptidyl peptidase-1	CTSC	NP_034112
Dipeptidyl peptidase-2	DPP7	NP_114031
Dipeptidyl peptidase-4	DPP4	NP_001153015
Endoplasmic reticulum aminopeptidase 1	ERAP1	NP_109636
Legumain	LGMN	NP_035305
Macrophage metalloelastase	MMP12	NP_032631
Matrix metalloproteinase-19	MMP19	NP_067387
Plasminogen	PLG	NP_032903
Tripeptidyl-peptidase 1	TPP1	NP_034036
Urokinase-type plasminogen activator	UPA	NP_032899
**Lysosomal enzymes** (15 proteins)		
Acid ceramidase	ASAH1	NP_062708
Alpha-N-acetylgalactosaminidase	NAGA	NP_032695
Beta-galactosidase	GLB1	NP_033882
Beta-glucuronidase	GUSB	NP_034498

Beta-hexosaminidase subunit alpha	HEXA	NP_034551
Beta-hexosaminidase subunit beta	HEXB	NP_034552
Deoxyribonuclease-2-alpha	DNASE2	NP_034192
Lysosomal alpha glucosidase	GAA	NP_032090
Lysosomal alpha mannosidase	MAN2B1	NP_034894
N(4)-(beta-N-acetylglucosaminyl)-L-asparaginase	AGA	NP_001005847
N-acetylgalactosamine-6-sulfatase	GALNS	NP_057931
N-acetylglucosamine-6-sulatase	GNS	NP_083640
N-acylethanolamine-hydrolyzing acid amidase	NAAA	NP_080248
Palmitoyl-protein thioesterase 1	PPT1	NP_032943
Tartrate-resistant acid phosphatase type 5	ACP5	NP_001095874
**Chemokines/Growth factors** (11 proteins)		
C-C motif chemokine 6	CCL6	NP_033165
C-C motif chemokine 7	CCL7/MCP3	NP_038682
C-C motif chemokine 9	CCL9	NP_035468
Connective tissue growth factor	CTGF	NP_034347
Granulins	GRN	NP_032201
Inhibin beta A chain	INHBA	NP_032406
Macrophage migration inhibitory factor	MIF	NP_034928
Mesencephalic astrocyte-derived neurotrophic factor	ARMET	NP_083379
Platelet-derived growth factor subunit A	PDGFA	NP_032834
Transforming growth factor beta-1	TGFB1	NP_035707
UPF0556 protein C19orf10 homolog	SF20	NP_543027
**Macrophage/M2 polarization markers** (6 proteins)		
Arginase-1	ARG1	NP_031508
Chitinase 3-like 3	CHI3L3	NP_034022
Galectin-3 (Mac-2)	GAL3	NP_034835
Lysozyme C-2	LYZ2	NP_059068

Macrophage mannose receptor 1	MRC1/CD206	NP_032651
Protein S100-A9	S100A9	NP_033140
**ECM proteins** (6 proteins)		
Collagen alpha-1(I) chain	COL1A1	NP_031768
Fibronectin	FN	NP_001263337
Laminin subunit alpha-5	LAMA5	NP_001074640
Laminin subunit beta-1	LAMB1	NP_032508
Laminin subunit beta-2	LAMB2	NP_032509
Laminin subunit gamma-5	LAMC1	NP_034813
**Protease inhibitors** (4 proteins)		
Alpha-2-macroglobulin-P	A2M	NP_783327
Antileukoproteinase	SLPI	NP_035544
Antithrombin-III	ATIII	NP_543120
Cystatin-C	CST3	NP_034106

Table S2

Proteins with signal sequence identified in <40KDa fractions from conditioned media generated from whole lung of BVE mice at 6 weeks p.p. (excluding proteins identified in IMC conditioned media or conditioned media from whole lung of $Braf^{WT}$ mice at 6 weeks p.p.)

Protein name	Symbol	NCBI Accession no.	Mass (KDa)	Known function(s)
Abhydrolase 11	ABHD11	NP_660250	34	Member of α/β hydrolase fold domain-containing family
Chitinase 3-like 1	CHI3L1	NP_031721	43	Member of chitinase family of digestive enzyme
Cut divalent cation tolerance homologue	CUTA	NP_080583	19	Copper tolerance in *E.coli*
Cytochrome b5 reductase 3	B5R<br>DIA1	NP_084063	34	Electron donor for cytochrome b5
Hyaluronan synthase 1	HAS1	NP_032241	65	Membrane-bound enzyme; produces hyaluronan at cell surface
Leucine rich repeat and fibronectin type III domain containing 4	LRFN4	NP_700437	67	Integral membrane protein member of Lrfn family
N-acetylgalactosaminyltransferase 12	GLT12	NP_766281	66	Catalyzes the initial reaction in O-linked oligosaccharide biosynthesis
Niemann Pick's Type C2	NPC2	NP_075898	16	Cholesterol binding protein
Protein disulphide isomerase I	PDIA1	NP_035162	57	ER resident protein catalysing the formation and breakage of disulphide bonds
Protein disulphide isomerase 6	PDIA6	NP_082235	48	ER resident protein catalysing the formation and breakage of disulphide bonds

Ribonuclease, RNase A family, 4	RNASE4	NP_957691	17	Pancreatic ribonuclease family member
SEC22 vesicle trafficking protein homolog B	SEC22B	NP_035472	25	Member of Sec22 family of vesicle trafficking proteins
Stanniocalcin 1	STC1	NP_033311	28	Calcium & phosphate metabolism/oxidative stress

SUPPLEMENTARY FIGURE LEGENDS

Figure S1. Senescence marker expression in BVE lung

A. Senescence-associated β-galactosidase (SA-βgal) staining of BVE lung sections at the senescent stage. SA-βgal staining was detected in the stroma, but not in tumours. Scale bars, 100μm.

B. Immunoblot analysis of whole lung lysates from BVE mice at the pre-senescent (4wk of age) and senescent (10wk of age) stages for CDK inhibitor ($p16^{INK4a}$/$p21^{CIP1}$) and γH2AX detection. $p21^{CIP1}$ and γH2AX were up-regulated at the senescent stage, but $p16^{INK4a}$ was expressed at very low levels at both stages. ERK2 blot and total protein staining by Amido Black (right) served as loading controls.

C. Immunoblot analysis of whole cell lysates of IMCs and AT2 cells purified from BVE lung at the senescent stage (10wk of age). γH2AX expression was much higher in AT2 cells than IMCs, whereas $p21^{CIP1}$ was detected in both IMCs and AT2 cells. $p16^{INK4a}$ was detected only in IMCs, indicating that $p16^{INK4a}$ expression in senescent AT2 cells is low, if any at all. The ERK2 blot served as a loading control.

D. $p21^{CIP1}$ Immunohistochemistry of lung sections from BVE mice at the senescent stage (10wk of age). Negative control staining (left) was performed by omitting the primary antibody incubation. A section from mouse skin papilloma (right) serves as a control showing strong $p21^{CIP1}$ staining in the epithelium as well as low staining in non-epithelial tissues. Asterisks in the middle photograph show intravascular red blood cells not stained for $p21^{CIP1}$, indicating the specificity of this staining condition. Scale bars, 100μm.

E. Immunoblot analysis of whole lung lysates from BVE mice at 4 and 10wk of ages as well as whole cell lysates of IMCs/AT2 cells purified from the BVE lung at the senescent stage (10wk of age) for $p19^{ARF}$ detection. $p19^{ARF}$ was not detected in any samples under these conditions whereas there is a clear positive signal for $p19^{ARF}$ in the lysate from immortalized MEFs with acquired p53 mutation (+control). Asterisks indicate non-specific staining observed at 25 and 35kDa.

Figure S2. Cell fractionation from BVE lung

A. A schematic diagram of the cell fractionation method used to establish purified stroma IMCs and AT2 cells as well as tumour cell aggregates for *ex vivo* characterization is shown. We utilised the differences in their sensitivities to proteolytic enzymes, cell densities, and adhesiveness to separate these three populations. Of note, stroma IMCs are highly adhesive to the culture plate but AT2 cells form clusters or large aggregates that start adhering to culture plates after 2 days.

B. Phase-contrast images of freshly isolated IMCs (left) and cultured AT2 cells (middle, on day 3 of culture) from a BVE mouse at 10wks of age, and a phase-contrast image of tumour cell aggregates from a BVE mouse at 3wks of age, grown for 10 days in culture (right). Of note, lung tissue from BVE mice at the senescent stage are needed to efficiently purify stroma IMCs, whereas those from animals at the pre-senescent stages are preferable for culture of tumour aggregates. All images were taken at 20x objective. Scale bar, 100 μm.

C. A photograph of freshly isolated AT2 cells stained by the modified Papanicolau method (100x objective, scale bar, 25 μm). The cytoplasmic granules stained in dark blue are lamellar bodies containing pulmonary surfactants characteristic of AT2 cells. An inset shows negative Papanicolau staining of IMCs.

D. Flow cytometry analysis of intracellular Surfactant C (SPC) expression in freshly isolated AT2 cells. SPC is a marker specific for AT2 cells. To determine the background staining level, we performed control staining by omitting primary anti-SPC antibody staining (left, No 1° Ab).

Figure S3. Cell surface marker analysis of wild-type lung and spleen cells dissociated by collagenase/DNase treatment

A. Cells isolated from the wild-type lung by 3hr collagenase/DNase treatment followed by red blood cell lysis were analysed for cell surface CD11b, Gr1, F4/80, CD3, B220 and CD24 expression. $CD11b^{high}Gr1^{high}$ granulocytes, $CD11b^{high}F4/80^{+}$ cells, $CD3^{+}$ T-cells and $B220^{+}CD24^{+}$ B-cells were readily detected, demonstrating that the enzyme treatment does not affect cell surface expression of the marker proteins we examined.

B. Cells were isolated as in (A) except for omission of red blood cell lysis and were analyzed for cell surface TER119 expression. Low FSC cells enriched for red blood cells were gated for this analysis. TER119 expression was detectable on red blood cells treated with the enzymes.

C. Cells isolated from wild-type spleen by 30 min collagenase/DNase treatment followed by red blood cell lysis were analysed. CD86 expression in a small population of $CD11b^{low}$ cells was detectable in the enzyme-treated cells without inducing activation.

Figure S4. Lung fibroblast isolation from BVE lung and co-culture with AT2 cells

A. Morphological characteristics of lung fibroblasts developed from BVE lung in culture. Phase-contrast microphotographs taken with 10x (left) or 40x (middle) objective show enrichment of lung fibroblasts in total lung culture at passage 5. The right microphotograph shows α-smooth muscle actin (αSMA) immunofluorescence obtained by confocal imaging and merged with a DIC image. Lung fibroblasts isolated by this method express αSMA fibres, an activated myofibroblast marker characteristic of tumour-associated fibroblasts. Scale bars, 100μm (left) or 25μm (middle and right).

B. Morphology and cell densities of IMCs and lung fibroblasts after 48h of co-culture with AT2 cells using a transwell culture plate. The fibroblasts developed more confluent cultures during the co-culture even though they were plated at a lower density ($6x10^5$/well) than IMCs ($2x10^6$/well). Phase-contrast microphotographs were taken after removal of insert wells containing AT2 cells at 48h of co-culture. Scale bars, 50μm. Of note, fibroblast contamination was rarely observed in the IMC culture.

C. Flow cytometry analysis of BrdU incorporation in AT2 cells co-cultured with IMCs or lung fibroblasts as in B. Co-culture with lung fibroblasts modestly increased BrdU incorporation in AT2 cells (right) compared to the transwell culture without IMCs/fibroblasts (left), but this effect was much weaker than co-culture with IMCs (middle).

Figure S5. Immunoblot analysis of proteins secreted by IMCs

A. To validate the mass spectrometry identification of PDGFA as a protein secreted by IMCs, immunoblot analysis of IMC-CM obtained after 72h culture in DMEM/10%FCS, was performed. Cell-free media (DMEM/10%FCS) was loaded to confirm the PDGFA protein levels in 10% FCS. Total protein staining by Amido Black served as a loading control. The bands corresponding to YM1 and lysozyme C2 were confirmed by mass spectrometry. Of note, cell-free media shows some proteins derived from FCS.

B. To compare TGFβ and CCL6 secretion levels between IMCs and lung fibroblasts, the cells were cultured in serum-free DMEM for 72hrs. IMC-CM and lung fibroblast conditioned media (LF-CM) were collected and concentrated using Amicon® Ultra-4 Centrifugal Filter Units. The final volume of each concentrated CM was adjusted by intracellular protein abundance of the cells in each culture. TGFβ was detected in both CM, but more TGFβ was secreted by IMCs (left). In contrast, CCL6 was detected only in IMC-CM (right), indicating that IMCs are the major cell type secreting this chemokine. Total protein staining by Amido Black served as a loading control, showing distinct secretory protein profiles of the IMCs and lung fibroblasts.

Figure S6. Tumour burden evaluation by flow cytometry and tumour area quantification

A. Correlations between lung weights and CD11c+/SPC+ cell numbers in wild-type and AdCre-induced tumour-bearing lungs. The data for left lobe weights and CD11c+/SPC+ cell numbers per left lobe were collected from 4 wild-type mice and the 15 mice used in Fig 4F and 6G. Correlations of left lobe weights to SPC+ cell numbers (left), CD11c+ cell numbers (middle) and the sum of SPC+ cells and CD11c+ cells (right) are shown. Pearson's correlation coefficient (R) is indicated for each analysis.

B. Tumour burden quantification of CCR1 inhibitor-treated mice by %tumour area calculation (corresponding to Fig 4F). %tumour area was calculated using whole lung (right lobe) images as described in Expanded View Methods. Representative whole lung images for vehicle and CCR1 inhibitor (CCR1i)-treated mice are indicated on the right.

C. Tumour burden quantification of AdCre-induced $Npc2^{+/hypo}Braf^{+/LSL\text{-}V600E}$ mice by %tumour area calculation (corresponding to Fig 6G). %tumour area was calculated as in B. Representative whole lung images for $Npc2^{+/+}$ (+/+) and $Npc2^{+/hypo}$ (+/hypo) mice are indicated on the right.

D. Correlation between the sum of CD11c+/SPC+ cell numbers and %tumour area in AdCre-induced tumour-bearing lungs analyzed in B and C. Pearson's correlation coefficient (R) is indicated on the graph.

Figure S7. Increased T-cells in the lungs of wild-type mice administrated with AdCre

A. Total lung cells harvested from wild-type mice (n=6) at 9-13 weeks after nasal inhalation of AdCre ($5x10^7$ pfu) were analysed by flow cytometry for CD4, CD8a and B220 surface expression, and compared with wild-type mice without AdCre administration (n=4). A statistically significant increase of T-cells (CD4+CD8) was observed in AdCre-treated mice.

B. Total lung cells harvested from $BRAF^{LSL-V600E}$ mice (n=5) at 9-10 weeks after nasal inhalation of AdCre ($5x10^7$ pfu) were analysed by flow cytometry for CD4 and CD8a surface expression, and compared with BVE ($CreER^T$) mice without AdCre administration (7-10 wk p.p., n=3). A statistically significant increase of T-cells (CD4+CD8) was observed in AdCre-treated mice.

Figure S8. Identification of secreted proteins in whole lung conditioned media

A. A schematic diagram of concentrated whole lung-conditioned media (WL-CM) preparation. Lung tissues from *Braf*$^{+/+}$*;CreER*^{TM} (WT) and BVE (VE) mice at 6 weeks p.p. were passed through a 70 μm cell strainer to obtain a cell suspension, and cultured in serum free media for 24h. Collected media was concentrated using Amicon® Ultra-4 Centrifugal Filter Units to obtain concentrated WL-CM.

B. WL-CM from WT and VE mice resolved by SDS-PAGE. Four protein fractions (boxed) were increased in VE samples and were analyzed by mass spectrometry. A full list of 13 identified secreted proteins is provided in Table E2.

Figure S9. Analysis of *Npc2*$^{+/hypo}$ BVE lung at the pre-senescent stage

A. Quantification of SPC+ AT2 cells per lung in *Npc2*$^{+/+}$ (+/+) and *Npc2*$^{+/hypo}$ (+/hypo) BVE mice at 5-6 weeks p.p. (n=6).

B. *In vivo* BrdU incorporation of SPC+ AT2 cells in *Npc2*$^{+/+}$ (+/+) and *Npc2*$^{+/hypo}$ (+/hypo) BVE mice at 5-6 weeks p.p. (left, n=3). 2mg/mouse BrdU was i.p. injected and lung tissues were harvested 5h after injection. Representative flow cytometry plot/histograms are indicated (right).

Figure S10. Filipin and organelle marker staining of freshly isolated *Npc2*$^{+/hypo}$ IMCs

A. Freshly isolated *Npc2*$^{+/hypo}$ IMCs were stained with filipin followed by LAMP1/EEA1 immunostaining with Alexa568-conjugated secondary antibodies, and imaging by confocal laser scanning microscopy (CLSM). Scale bars, 10μm. Free cholesterol-rich structures (strongly stained with filipin) were not co-stained with the lyso-endosome markers. Of note, strong filipin staining was mainly observed at the ventral side of the cells (see Fig S11), where EEA1 signal was relatively weak.

B. Filipin-stained *Npc2*$^{+/hypo}$ IMCs were immunostained for Giantin (Golgi cisternae marker) as in A, and imaged by CLSM. Scale bars, 10μm. Giantin staining was not co-localized at free cholesterol-rich coarse structures (strongly stained with filipin) at the ventral side of the cells, though some co-localization at the plasma membrane was observed (top panels). Strong Giantin staining was observed at perinuclear regions (bottom panels), likely representing Giantin localization at the Golgi.

C. CLSM imaging of *Npc2*$^{+/hypo}$ IMCs stained with filipin followed by TGN46 (trans-Golgi network marker) immunostaining as in A. Scale bars, 10μm. As with Giantin, TGN46 co-localizes with filipin staining at the plasma membrane but not at coarse structures strongly stained with filipin.

Figure S11. Coarse structures strongly stained with filipin localise at the ventral side of *Npc2*$^{+/hypo}$ IMCs

A. CLSM imaging of freshly isolated *Npc2*$^{+/hypo}$ IMCs stained for free cholesterol (filipin) and LAMP2 as in Fig S10. Orthogonal (X-Z and Y-Z) images at the position indicated by dashed lines on the X-Y plane were generated by Z-stack reconstruction using Image J. Scale bar, 5μm. A free cholesterol-rich coarse structure (strongly stained with filipin) localizes near the ventral surface of the cell (arrows), surrounded by LAMP2-positive membranous structures.

B. Freshly isolated *Npc2*$^{+/hypo}$ IMCs were stained with filipin and Alexa647-phalloidin (for F-actin), and imaged by CLSM. Single-color images (top panels for filipin and F-actin in grey scale) and merged images with orthogonal views generated as in A (bottom panels) are indicated. Scale bars, 10μm. Relatively fine structures stained with filipin were partially associated with vertical F-actin-based columns (arrow heads) suggestive of podosomes, whereas coarse structures strongly stained with filipin (arrows) were not.

Figure S12. CCL6 and Golgi marker staining of *Npc2*$^{+/+}$ IMCs cultured with exogenous NPC2

A. *Npc2*$^{+/+}$ IMCs cultured with 50μg/ml bNPC2 for 48hrs were immunostained for TGN46 and CCL6 for CLSM imaging. Scale bars, 10μm. (Top panels) Vesicular CCL6 staining at perinuclear regions (arrow heads) and in the cytoplasm does not co-localize with TGN46 in bNPC2-treated IMCs that show peripheral, rather than perinuclear, TGN46 staining. (Bottom panels) In some bNPC2-treated IMCs, TGN46 staining was detected at perinuclear regions where CCL6 partially co-localizes with TGN46.

B. *Npc2*$^{+/+}$ IMCs were cultured as in A, and immunostained for Giantin and CCL6 for CLSM imaging. Scale bars, 10μm. Perinuclear structures in bNPC2-treated IMCs showing relatively stronger CCL6 signals were co-stained for Giantin (CCL6+Giantin+), whereas perinuclear vesicles weakly stained for CCL6 were negative for Giantin (CCL6+Giantin-), indicating that CCL6 distributes into two distinct compartments; the Golgi apparatus (Giantin+) and non-Golgi vesicles (Giantin-), at the perinuclear regions in bNPC2-treated IMCs.

Figure S13. CCL6/LAMP1 co-localisation in IMCs treated with exogenous NPC2 and Bafilomycin-A1

Fresh IMCs were treated with (middle panels) or without (top panels) 50μg/ml bNPC2 for 30min, chased in serum-free DMEM for 3hrs, and then treated with 200nM Bafilomycin-A1 (Baf-A1) for 24hrs. The treated cells were immunostained for CCL6 and LAMP1, and imaged by CLSM. Scale bars, 5μm. Bottom panels indicate enlarged images of the boxed area in the middle right image, highlighting LAMP1/CCL6 co-localization in bNPC2/Baf-A1 treated IMCs.

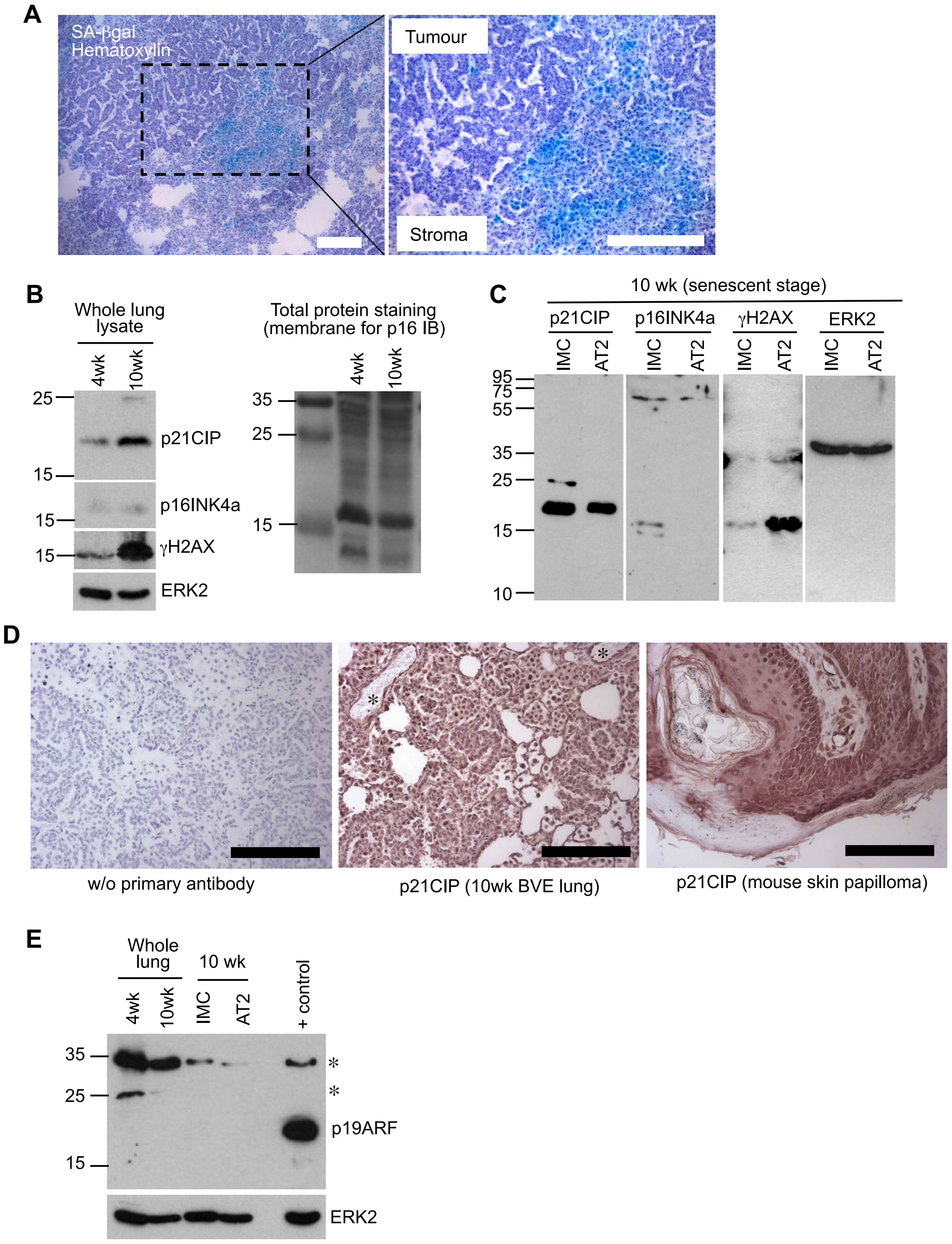
A
SA-βgal
Hematoxylin
Tumour
Stroma
B
Whole lung lysate
4wk
10wk
25
15
15
15
p21CIP
p16INK4a
γH2AX
ERK2
Total protein staining (membrane for p16 IB)
4wk
10wk
35
25
15
C
10 wk (senescent stage)
p21CIP
p16INK4a
γH2AX
ERK2
IMC
AT2
95
75
55
35
25
15
10
D
w/o primary antibody
p21CIP (10wk BVE lung)
p21CIP (mouse skin papilloma)
E
Whole lung
10 wk
4wk
10wk
IMC
AT2
+ control
35
25
15
p19ARF
ERK2

Figure S1

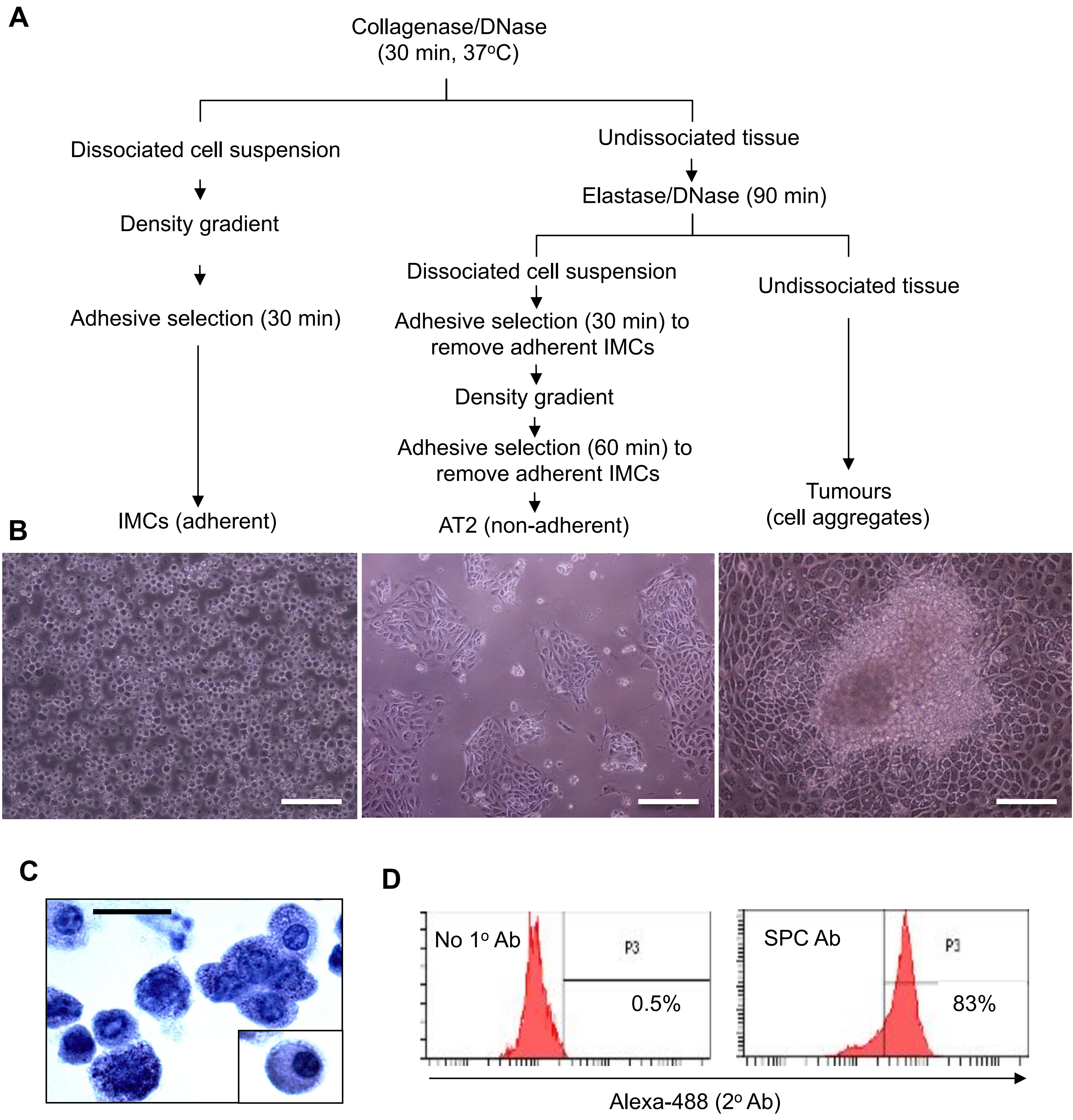
A
Collagenase/DNase
(30 min, 37°C)
Dissociated cell suspension
Density gradient
Adhesive selection (30 min)
IMCs (adherent)
Undissociated tissue
Elastase/DNase (90 min)
Dissociated cell suspension
Adhesive selection (30 min) to remove adherent IMCs
Density gradient
Adhesive selection (60 min) to remove adherent IMCs
AT2 (non-adherent)
Undissociated tissue
Tumours
(cell aggregates)
B
C
D
No 1° Ab
P3
0.5%
SPC Ab
P3
83%
Alexa-488 (2° Ab)

Figure S2

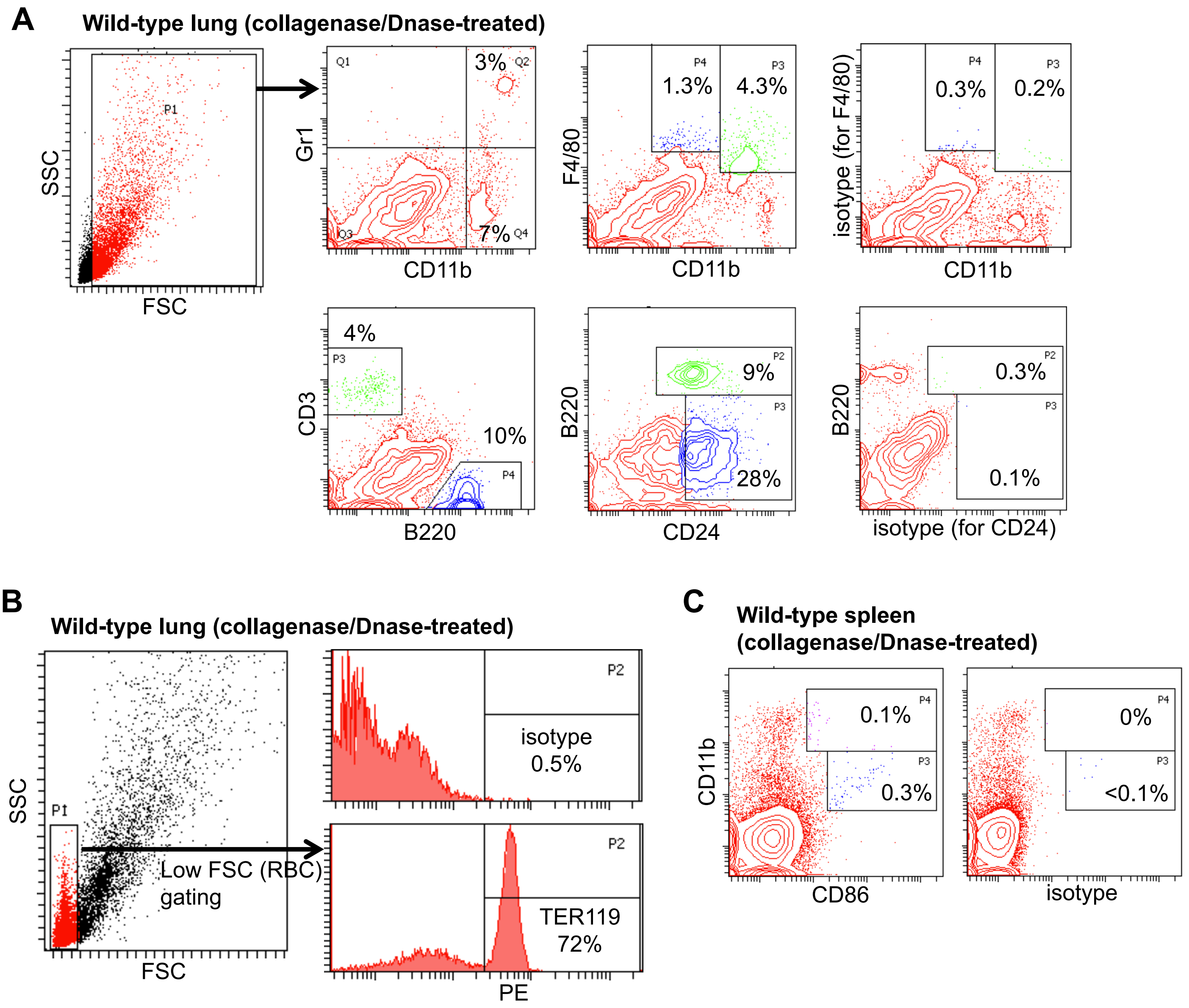

A
Wild-type lung (collagenase/Dnase-treated)
SSC
FSC
P1
Gr1
CD11b
Q1
Q2
Q3
Q4
3%
7%
F4/80
P4
P3
1.3%
4.3%
isotype (for F4/80)
0.3%
0.2%
CD3
B220
4%
10%
B220
CD24
P2
9%
28%
isotype (for CD24)
0.3%
0.1%
B
Wild-type lung (collagenase/Dnase-treated)
SSC
FSC
P1
Low FSC (RBC)
gating
P2
isotype
0.5%
TER119
72%
PE
C
Wild-type spleen
(collagenase/Dnase-treated)
CD11b
CD86
0.1%
0.3%
isotype
0%
<0.1%

Figure S3

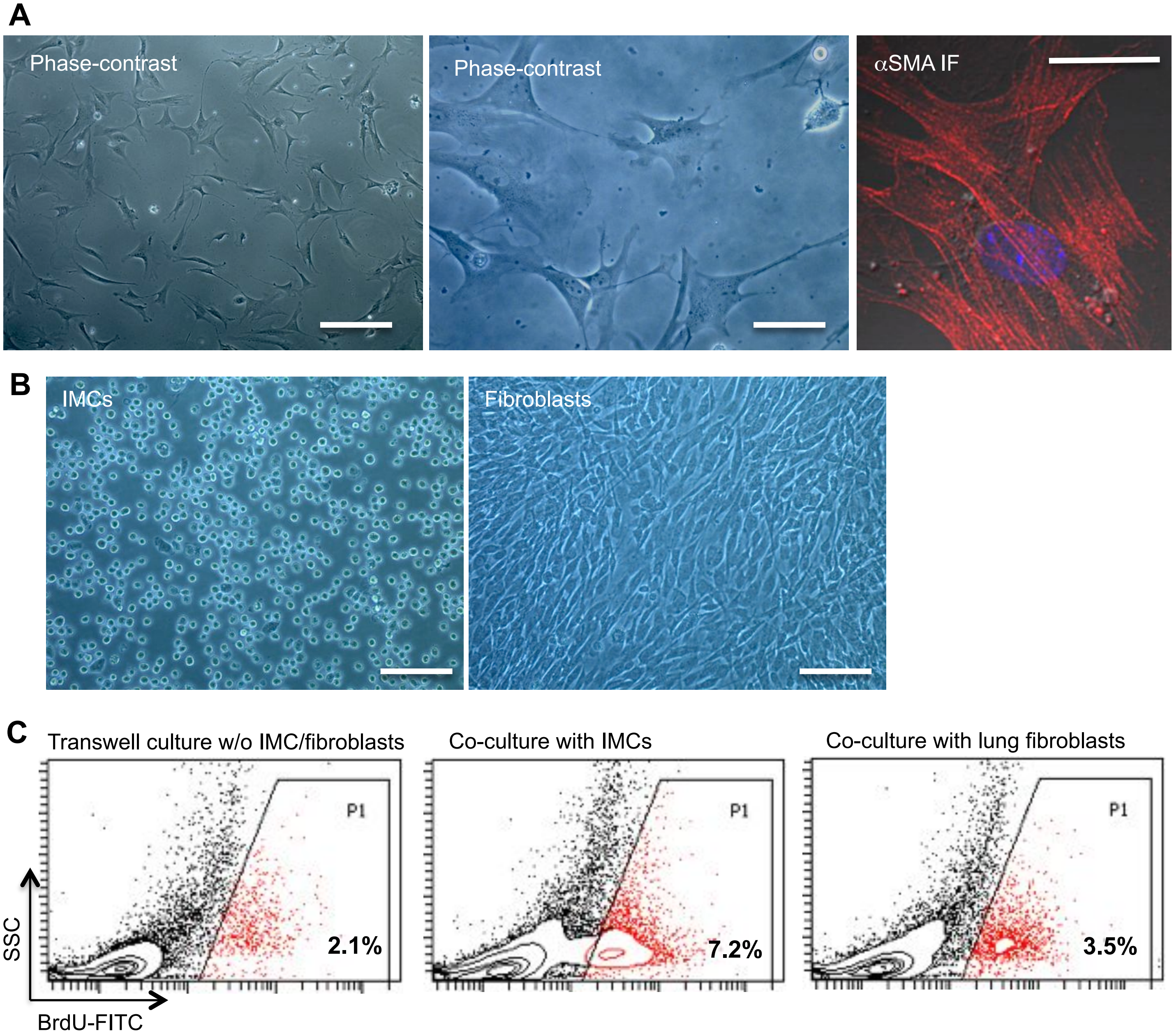
A
Phase-contrast
Phase-contrast
αSMA IF
B
IMCs
Fibroblasts
C
Transwell culture w/o IMC/fibroblasts
Co-culture with IMCs
Co-culture with lung fibroblasts
P1
P1
P1
2.1%
7.2%
3.5%
SSC
BrdU-FITC

Figure S4

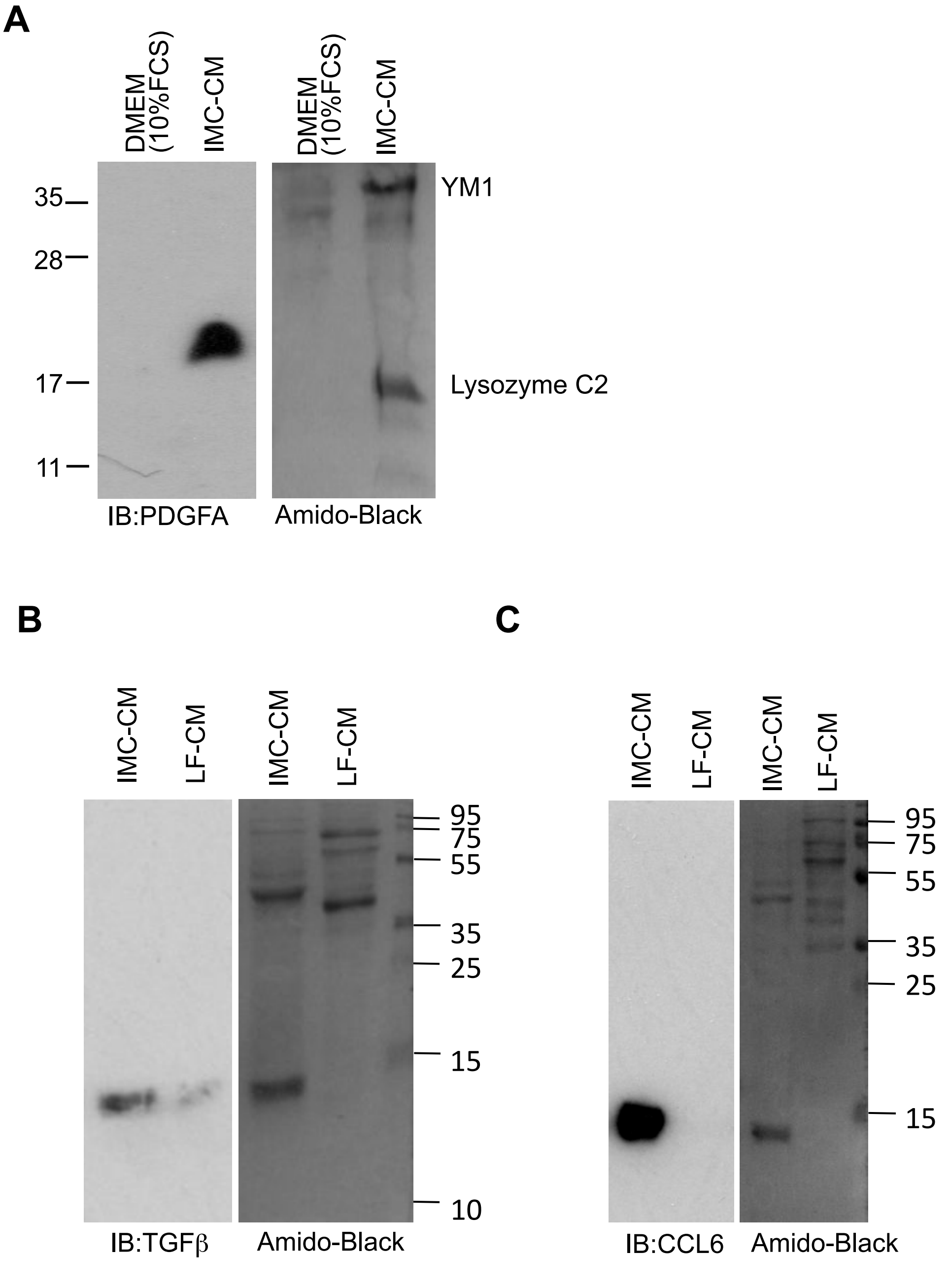
A
DMEM (10%FCS)
IMC-CM
DMEM (10%FCS)
IMC-CM
35
28
17
11
YM1
Lysozyme C2
IB:PDGFA
Amido-Black
B
IMC-CM
LF-CM
IMC-CM
LF-CM
95
75
55
35
25
15
10
IB:TGFβ
Amido-Black
C
IMC-CM
LF-CM
IMC-CM
LF-CM
95
75
55
35
25
15
IB:CCL6
Amido-Black

Figure S5

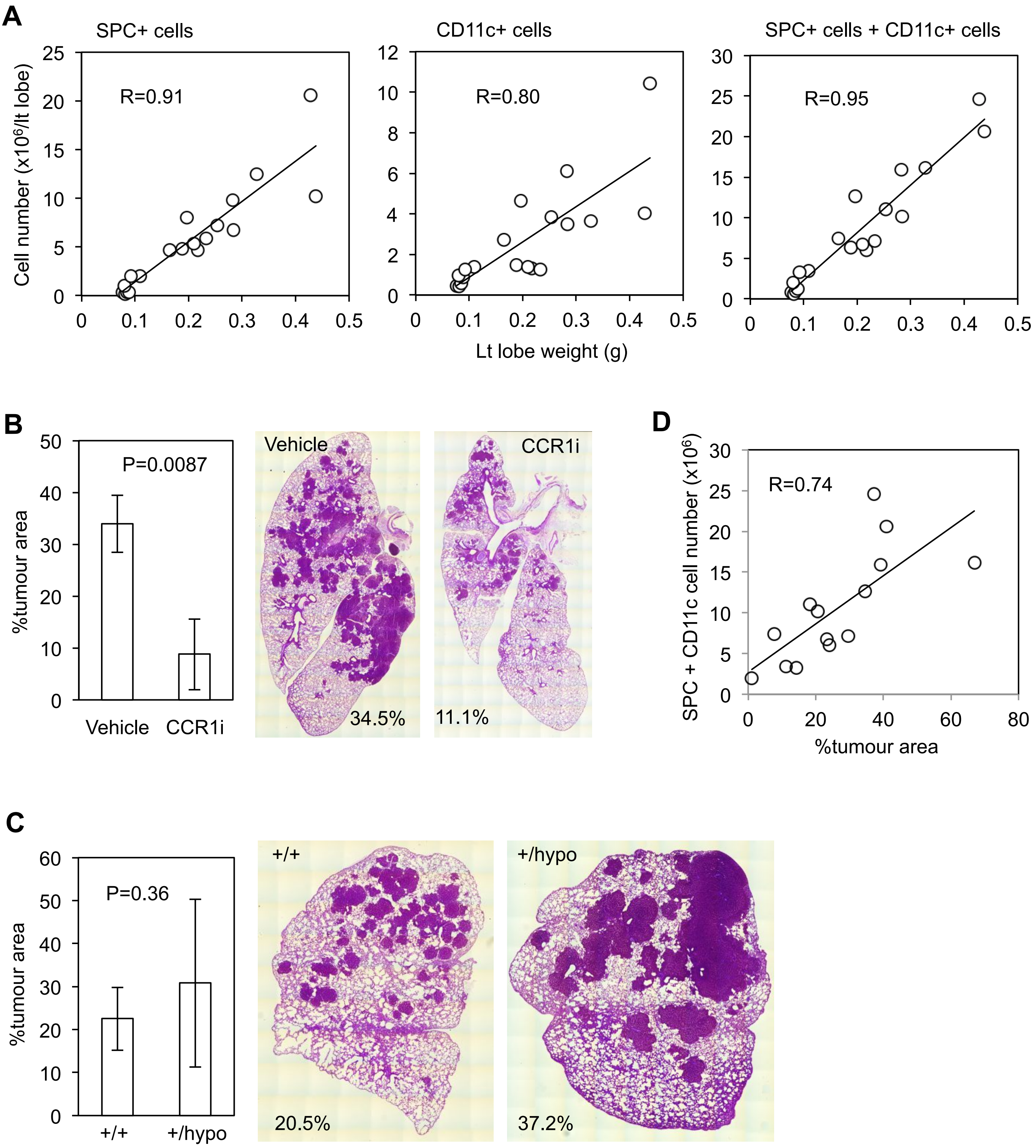
A
SPC+ cells
CD11c+ cells
SPC+ cells + CD11c+ cells
R=0.91
R=0.80
R=0.95
Cell number (x10^6/lt lobe)
Lt lobe weight (g)
B
P=0.0087
%tumour area
Vehicle
CCR1i
34.5%
11.1%
D
R=0.74
SPC + CD11c cell number (x10^6)
%tumour area
C
P=0.36
%tumour area
+/+
+/hypo
20.5%
37.2%

Figure S6

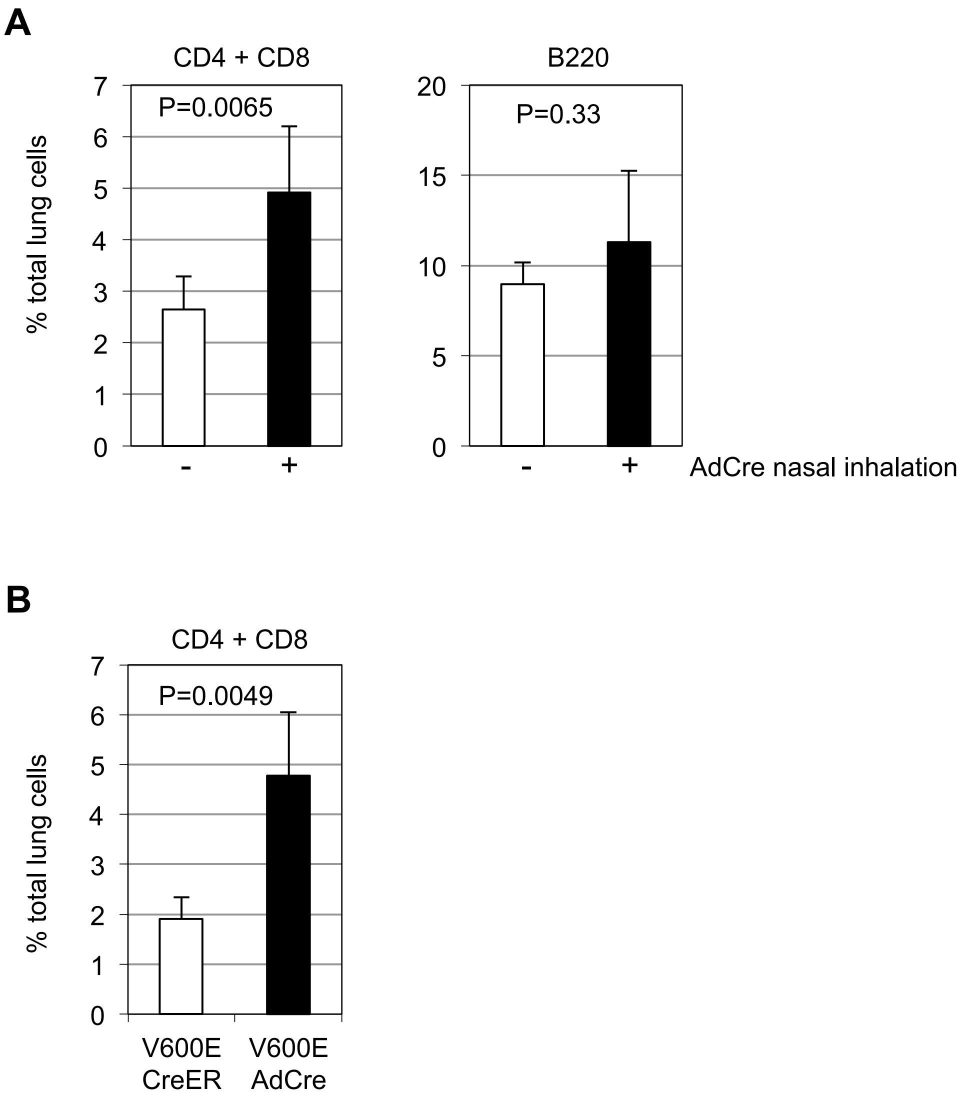
A
CD4 + CD8
P=0.0065
% total lung cells
B220
P=0.33
AdCre nasal inhalation
B
CD4 + CD8
P=0.0049
% total lung cells
V600E
CreER
V600E
AdCre

Figure S7

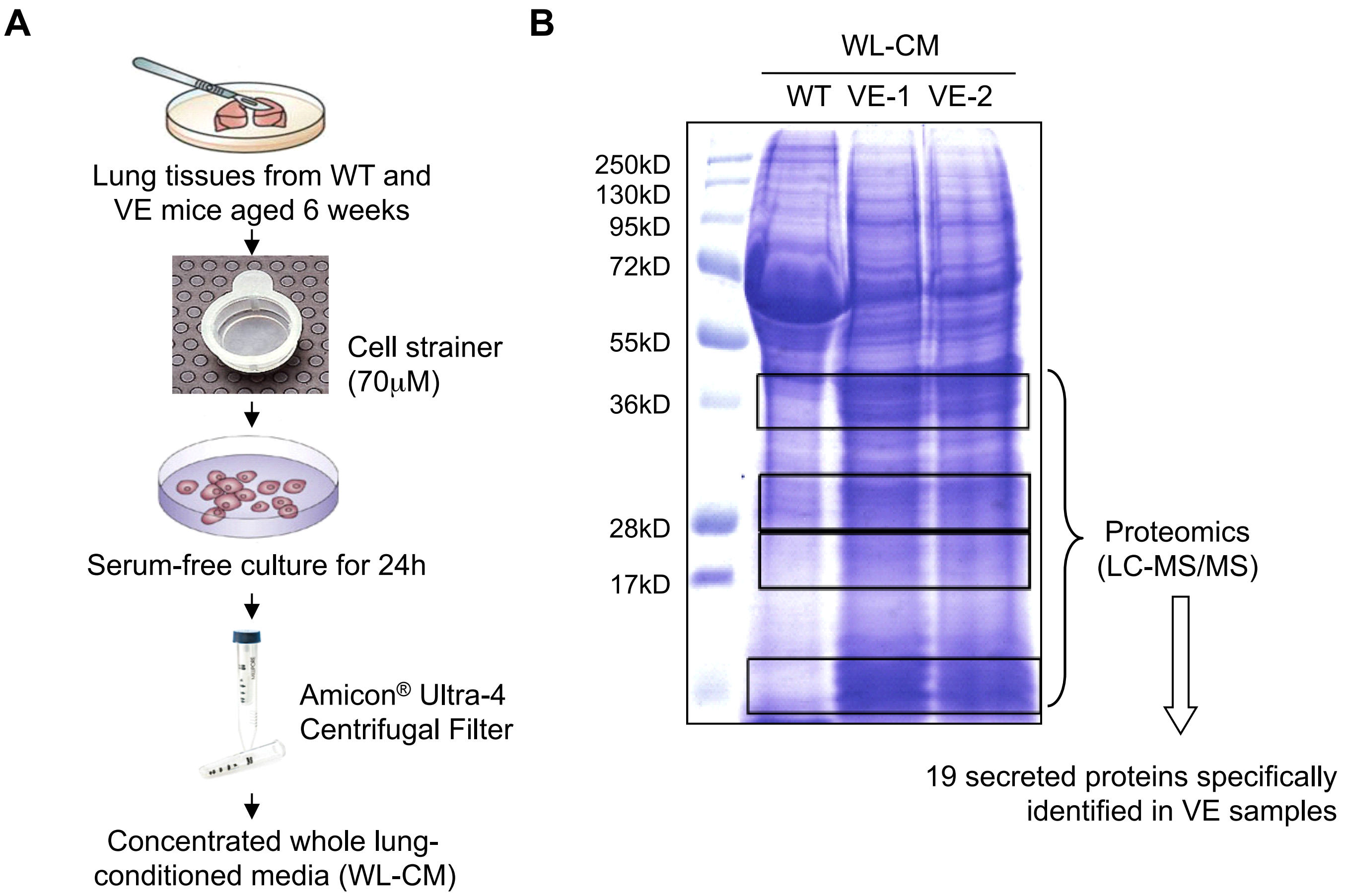
A
Lung tissues from WT and VE mice aged 6 weeks
Cell strainer (70μM)
Serum-free culture for 24h
Amicon® Ultra-4 Centrifugal Filter
Concentrated whole lung-conditioned media (WL-CM)
B
WL-CM
WT VE-1 VE-2
250kD
130kD
95kD
72kD
55kD
36kD
28kD
17kD
Proteomics (LC-MS/MS)
19 secreted proteins specifically identified in VE samples

Figure S8

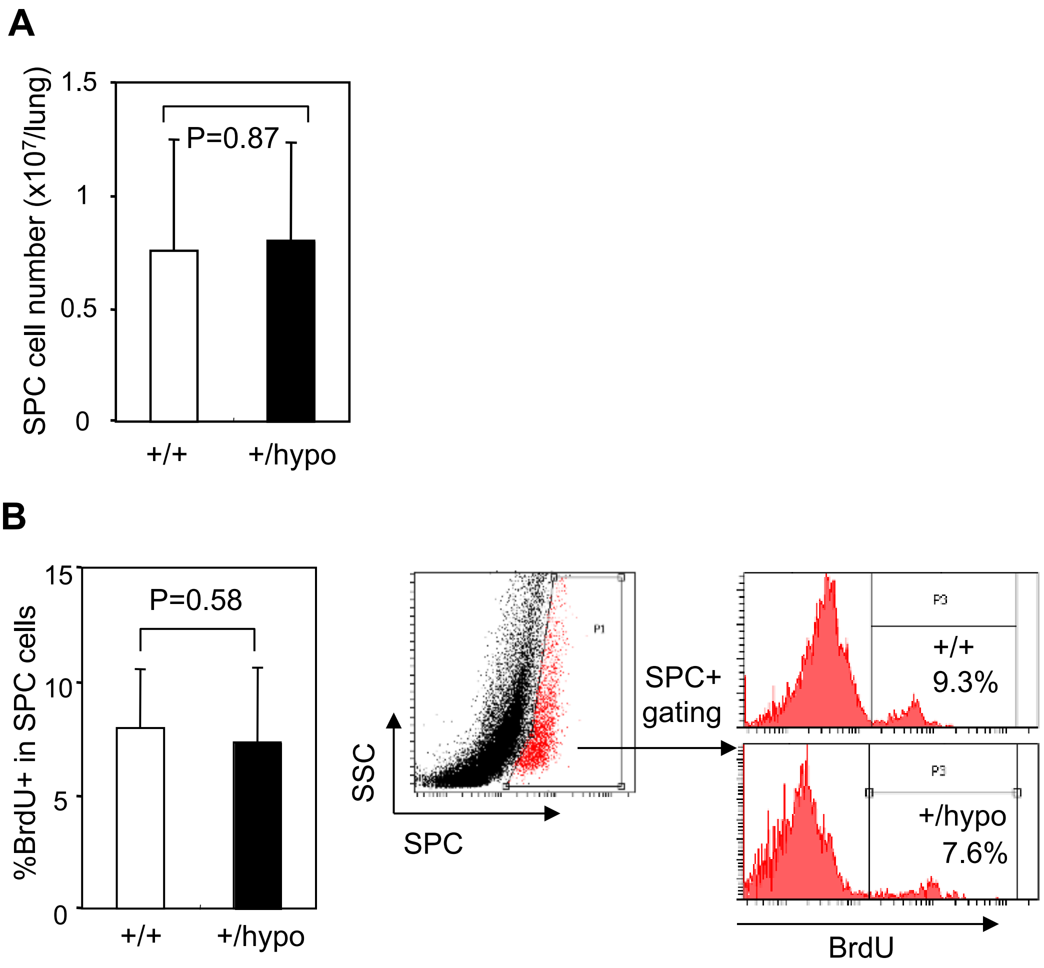
A
SPC cell number (x10^7/lung)
1.5
1
0.5
0
P=0.87
+/+
+/hypo
B
%BrdU+ in SPC cells
15
10
5
0
P=0.58
+/+
+/hypo
SSC
SPC
P1
SPC+
gating
P3
+/+
9.3%
+/hypo
7.6%
BrdU

Figure S9

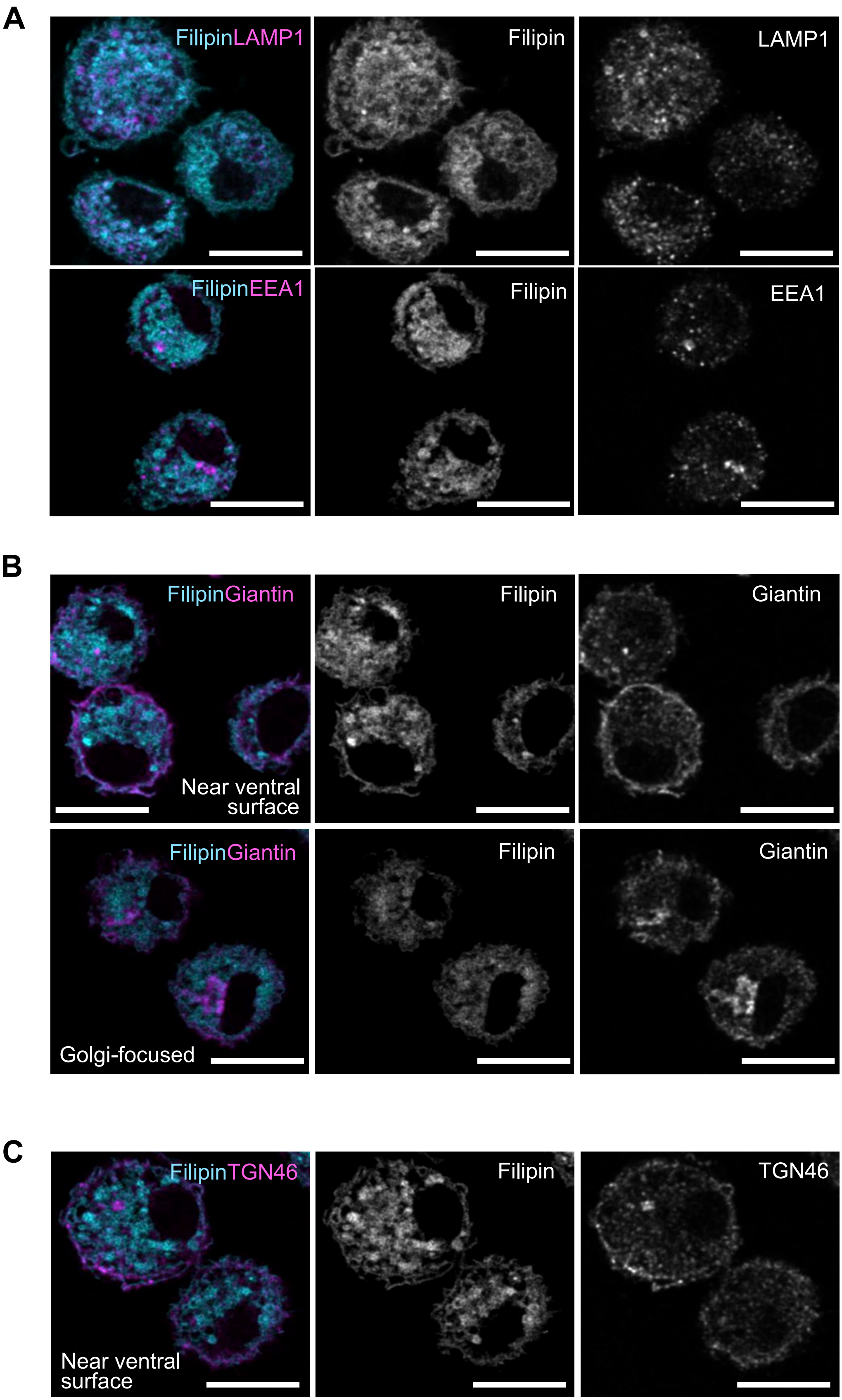
A
FilipinLAMP1
Filipin
LAMP1
FilipinEEA1
Filipin
EEA1
B
FilipinGiantin
Filipin
Giantin
Near ventral surface
FilipinGiantin
Filipin
Giantin
Golgi-focused
C
FilipinTGN46
Filipin
TGN46
Near ventral surface

Figure S10

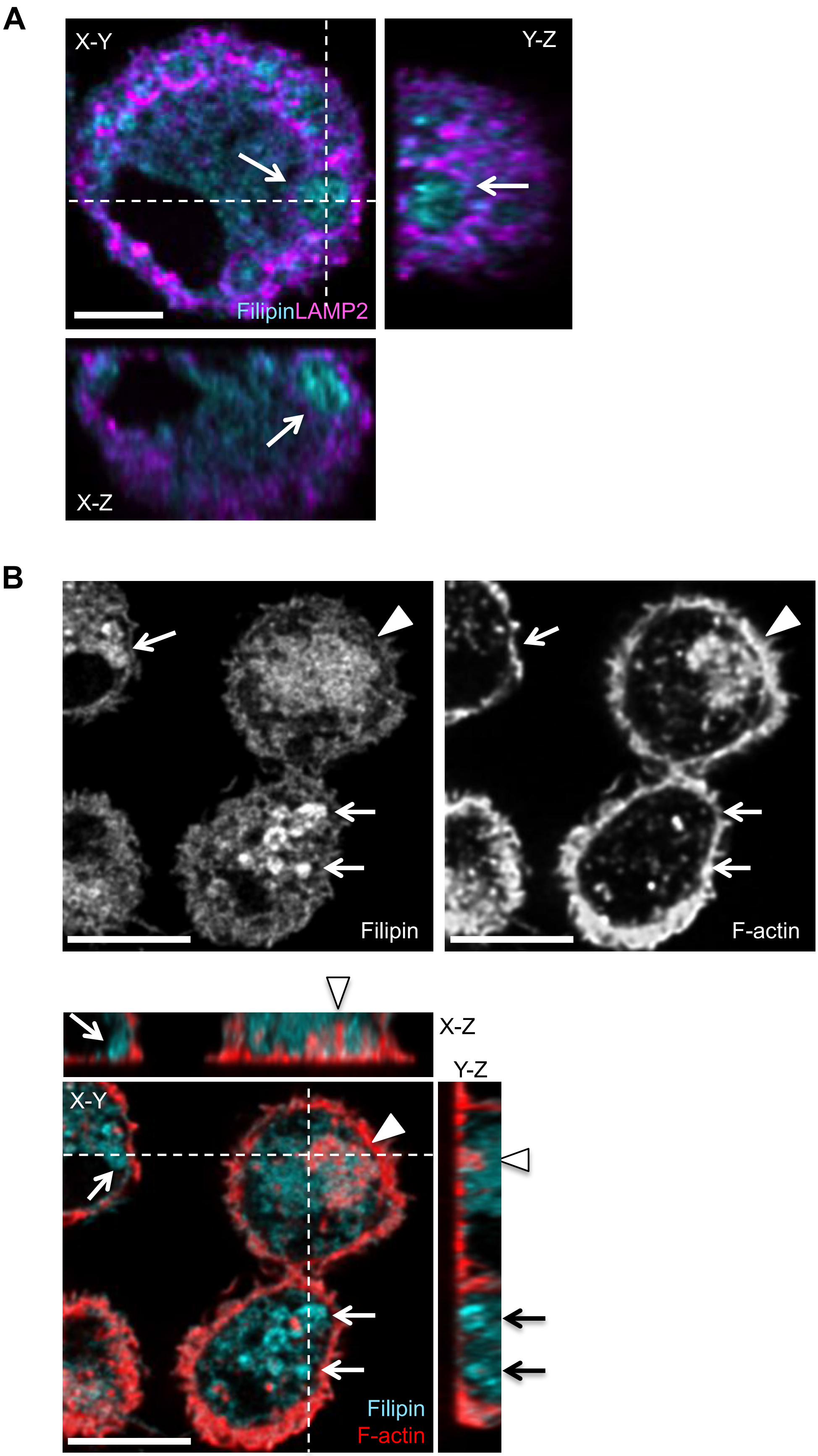
A
X-Y
Y-Z
FilipinLAMP2
X-Z
B
Filipin
F-actin
X-Z
Y-Z
X-Y
Filipin
F-actin

Figure S11

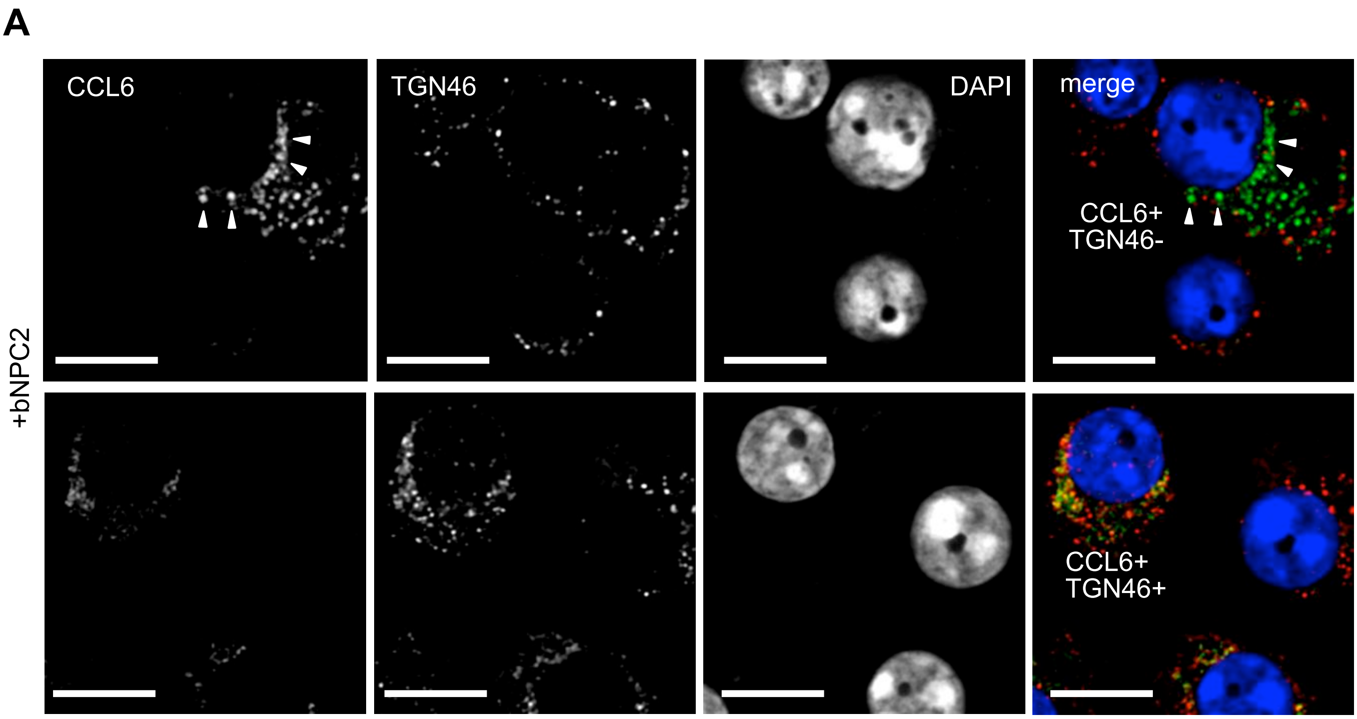
A
CCL6
TGN46
DAPI
merge
CCL6+
TGN46-
+bNPC2
CCL6+
TGN46+

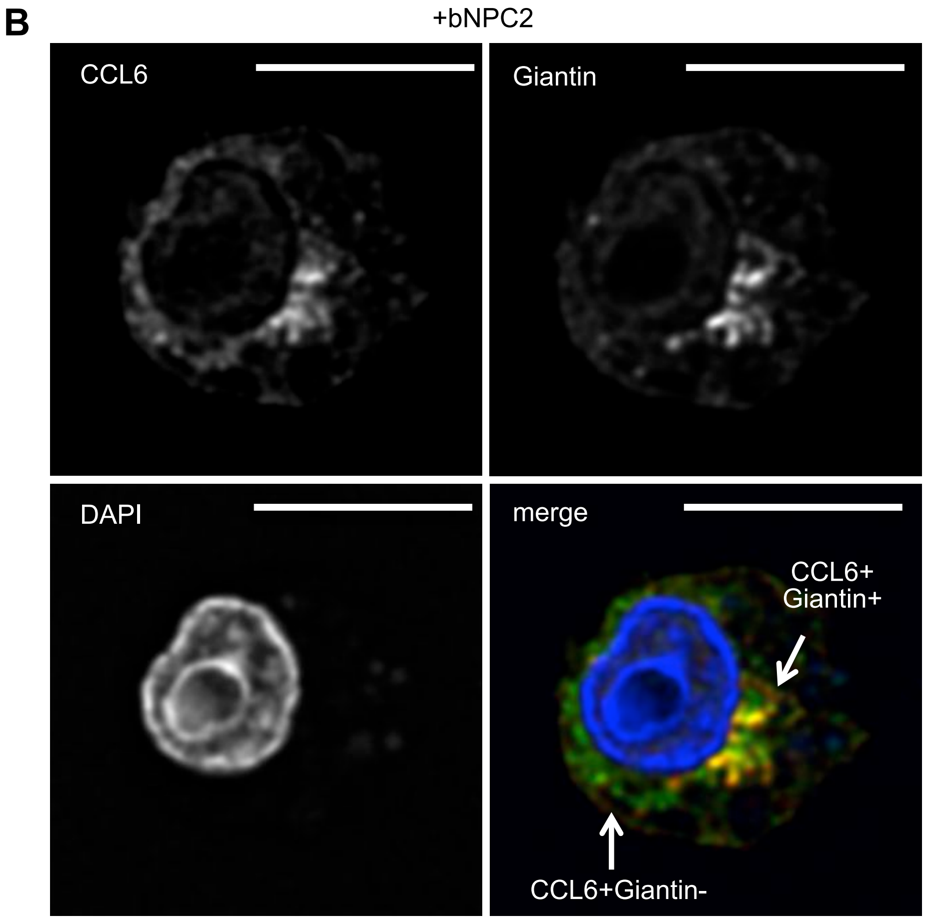
B
+bNPC2
CCL6
Giantin
DAPI
merge
CCL6+
Giantin+
CCL6+Giantin-

Figure S12

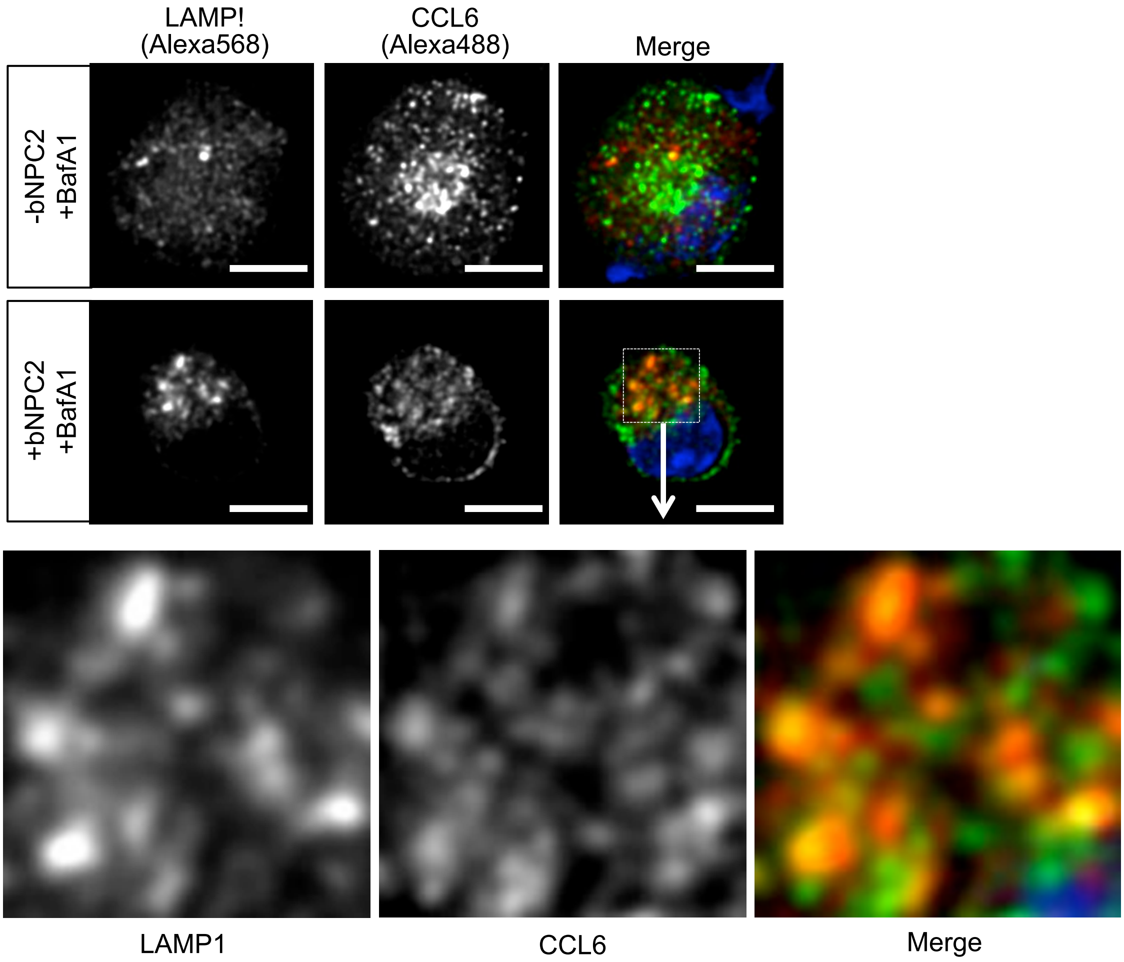
LAMP!
(Alexa568)
CCL6
(Alexa488)
Merge
-bNPC2
+BafA1
+bNPC2
+BafA1
LAMP1
CCL6
Merge

Figure S13
