# Supplementary material for: The cholesterol-binding protein NPC2 restrains recruitment of stromal macrophage-lineage cells to early-stage lung tumours
Source: EMBO Mol Med. 2015 Jul 16;7(9):1119–37. doi: 10.15252/emmm.201404838 (PMC4568947; doi:10.15252/emmm.201404838)
Supplement: Supplementary file 5 [file emmm0007-1119-sd5.pdf]

EMBO Molecular Medicine

# The cholesterol-binding protein NPC2 restrains recruitment of stromal macrophage-lineage cells to early-stage lung tumours

Tamihiro Kamata, Hong Jin, Susan Giblett, Bipin Patel, Falguni Patel, Charles Foster and Catrin Pritchard

*Corresponding author: Catrin Pritchard, University of Leicester*



**Review timeline:**

| | |
|---|---|
| Submission date: | 11 November 2014 |
| Editorial Decision: | 21 January 2015 |
| Revision received: | 01 May 2015 |
| Editorial Decision: | 03 June 2015 |
| Revision received: | 16 June 2015 |
| Accepted: | 17 June 2015 |



**Transaction Report:**

(Note: With the exception of the correction of typographical or spelling errors that could be a source of ambiguity, letters and reports are not edited. The original formatting of letters and referee reports may not be reflected in this compilation.)

*Editor: Roberto Buccione*

1st Editorial Decision 21 January 2015

Thank you for the submission of your manuscript to EMBO Molecular Medicine. We have now received comments from the Reviewers whom we asked to evaluate your manuscript.

We are very sorry that it has taken much more time than we would have liked to return a decision, but unfortunately we had difficulties securing appropriate (and willing) Reviewers and furthermore, in part due to the holiday season overlap, they delivered their evaluations with delay. I trust that the inevitable frustration due to this will be somewhat tempered by the fact that the Reviewers are all supportive of your work, albeit with different nuances and, in my opinion, offer valuable suggestions to improve your manuscript.

Reviewer 1 is concerned that your work as presented, lacks focus and a clear, conclusive message with respect to role of the macrophage lineage cells of the stroma and points to contradictory data. S/he also lists a few other important items for your action, including suggestions to improve data presentation, which, as you will see, appears to be a leitmotif in the Reviewers' criticisms.

Reviewer 2 is quite positive but is very critical of the overall data presentation, specifically regarding the lack of important experimental details (in this respect, please see my request for a checklist further below), the figures and figure captions. I agree that these aspects could be very much improved and without too much effort.

Reviewer 3 also mentions aspects of presentation and data quality, including lack of focus, for you to deal with. S/he also raises, however, specific concerns on the lack of sufficient experimental support for some important conclusions. Namely, s/he feels that the mechanism involving NPC-mediated CCL6 release requires stronger experimental evidence and that as such the conclusion are over-stated. The Reviewer offers various suggestions for you to act upon.

In conclusion, while publication of the paper cannot be considered at this stage, we would be pleased to consider a suitably revised submission, provided that the Reviewers' concerns are addressed as outlined above, including with additional experimental data where appropriate.

Please note that it is EMBO Molecular Medicine policy to allow a single round of revision only and that, therefore, acceptance or rejection of the manuscript will depend on the completeness of your responses included in the next, final version of the manuscript.

As you know, EMBO Molecular Medicine has a "scooping protection" policy, whereby similar findings that are published by others during review or revision are not a criterion for rejection. Although I clearly do not foresee such a delay in this case, I do ask you to get in touch with us after three months if you have not completed your revision, to update us on the status. Please also contact us as soon as possible if similar work is published elsewhere.

Finally, please note that EMBO Molecular Medicine now requires a complete author checklist (http://embomolmed.embopress.org/authorguide#editorial3) to be submitted with all revised manuscripts. Provision of the author checklist is mandatory at revision stage; The checklist is designed to enhance and standardize reporting of key information in research papers and to support reanalysis and repetition of experiments by the community. The list covers key information for figure panels and captions and focuses on statistics, the reporting of reagents, animal models and human subject-derived data, as well as guidance to optimise data accessibility. Please make sure that the relevant information is also included in the main manuscript text.

I look forward to receiving you revised manuscript as soon as possible.

***** Reviewer's comments *****

Referee #1 (Comments on Novelty/Model System):

The mouse model recapitulates a tumor initiating tumor mutation discovered in a subset of human lung cancers and the pathological features.

Referee #1 (Remarks):

The goal of this study is to understand role of tumor microenvironment in early cancer progression. Particularly, the authors focus on interactions between tumor and stroma during pre-cancerous development using the oncogenic BRAF (V600EBRAF)-driven model of lung adenocarcinoma, in which pre-cancerous progression of mutant cells can be identified. The major findings of the study are as follows:

1) The authors found a population of macrophage-lineage cells (named IMCs) in the stroma of premalignant tumor, which appears to be immature based on surface marker analysis but expresses several factors, including CC chemokines (e.g. CCL6). Blockade of CC chemokine receptor 1

(CCR1) drastically reduces accumulation of IMCs and tumor burden in the V600EBRAF mice.

2) Ex vivo experiments using primary cells showed that IMCs promote proliferation and cause transcriptional changes indicating EMT consistent with the phenotypes of tumor cells co-cultured with IMCs.

3) However, a different in vivo experiment, in which the compound V600EBRAF/NPC2hypomorphic mice were analyzed, the dramatic increase of IMCs in the stroma did not increase the tumor burden. Based on this observation, the authors concluded that the IMCs "are required for maintenance of early-stage lung tumors".

4) Additional ex vivo experiments demonstrated that NPC2 secreted from tumor cells acts on the IMCs in a paracrine manner to suppress release of CCL6, which would mediate recruitment of the IMCs to the developing premalignant tumor cells (see 1).

Overall, the study addresses very important questions and suggested a population of stroma cells that may play an important role in BRAF-driven tumor progression. Importantly, the study also found novel roles of CCR1 and NPC2 signaling in regulating the tumor stroma that may influence lung cancer progression. These findings would potentially have significant impacts on developing chemoprevention strategies.

However, a major concern about this study is that it appears to lack a conclusion at least regarding a potential role of IMCs. The conclusions from multiple experiments do not coherently lead to one larger conclusion but rather seem conflicting. It appears that the findings of 1) and 2) are somewhat contradictory to 3) and 4) as far as the role of IMCs is concerned: 1) and 2) suggest an oncogenic role of IMCs while 3) and 4) the opposite. Perhaps, the term "maintenance" used several times in the text to describe the role of IMCs is vague and needs to be better defined.

Minor points are also related to some of the authors' descriptions and conclusions.

a) Tumor burden is usually presented as the ratio of total tumor area to total lung area and the number of individual tumors in a given section (Fig. 5 and Fig. 7). Counting the number of AT2 cells may be less accurate because that count also include wild type AT2 cells, a majority of lung epithelial cells.

b) Fig. 1 does not have the legend for B.

c) Oncogene-induced senescence (OIS) is a key phenomenon during early tumor progression. The authors need to determine senescence phenotype using several standard markers, other than 'loss of Ki67, such as SA-beta-gal (Fig. 1).

d) The experiments in Fig. 2 need a control, other than nothing. In general, any stomal cells when con-cultured help tumor cells for survival and/or can change growth pattern. I believe there are other cell types in the stroma, perhaps other immune cells or lung fibroblast.

e) The authors suggest a role of IMCs in regulating the OIS phenotype but did not show any direct evidence. Perhaps this co-culture experiment can test that idea using standard markers of senescence.

f) In page 15, the title of the paragraphs does not represent the points being made below. I believe that the introduction of NPC2 and cholesterol trafficking helps understand the experiments to determine CCL6 but is not the focus.

Referee #2 (Remarks):

The presented work explores so far understudied area of cancer research and potentially opens a new and interesting way of understanding the problem for tumor development and interaction between tumor and microenvironment, which may also have an impact on the development new treatment strategies. The suggested model seems to be adequate, however the follow up study would be necessary to understand, whether this phenomenon also takes place for further cell and cancer types. Technically, this very interesting and potentially powerful study, however, suffers from inadequate figure structure and low quality figure captions as well as extremely scarce explanations for the experiments/antibodies used etc. The text and figure captions have to be seriously edited, and adjusted to form a readable story, which is now cannot be published in this form. Besides, some data might need better verification.

Please find below the selected questions/suggestions. The whole manuscript, however, has to be improved. Please note that the experiment for the figure 4 looks very strange and has to be at least repeated and the results displayed in the different way.

Figure 1

Fig 1: the figure 1c on the figure description obviously corresponds to 1b on the actual figure, and further on, 1c corresponds to 1d. Please correct.

Please mention briefly in the figure caption why the row of HE stainings was demonstrated along with te lung sample from AdCre+ mice similar to the manuscript text. This will promote the understanding of the figure.

Fig 1B. Please induce the wt control to demonstrate the basis level of Ki67 staining. Please induce all the age points in the graph along with the wt control in the graph on the right hand side.

There is no mention of the number of tested animals/samples for the each age point used for Ki67 detection and analysis, which is also required to evaluate the data robustness. Please provide the appropriate numbers.

Fig. 1C. The data display will be significantly enhanced when the Mac2 staining will be demonstrated on the section adjacent to the HE one, also showing both magnifications, which were used for the HE. In this way the morphological characteristics of the cells mentioned in the manuscript text can be compared to and supported by the Mac 2 staining.

Figure S1.
Fig. 1A. A sentence explaining the purpose of the cell isolation would significantly add to the readability.

Fig. S1B. It makes more sense to display similar magnification (-s) of the phase-contrast micrographs for the different cell isolates. When it is not quite representative, it might be helpful to include 2 magnifications for each cell type.

Fig. S1C. Why the Fr-2 cells were displayed? There is also no explanation in the text of the manuscript as I could found. Please provide brief description and reference for the Papanicolau stain, and what this stain is for.

Fig. 1D. Why was SP-C staining included? What does it show?

Figure 2.

Generally is quite advantageous to follow the story briefly by the figure captions, which also helps the readership to understand the authors' ideas. Please include very brief description of why you placed the giemsa cell stain also on the figure 2A caption. The same for Fig 2C, very brief mentioning for the purpose of the stain, as for Fig. 2D and 2E.

Fig. 2D. Would be really helpful to display the same cells for phase-contrast and giemsa stain, which is I hope not restrictively hard to achieve, but significantly enhance the convincing power of the images. Please also indicate, "phase-contrast" as you indicated giemsa on appropriate images to perform a uniform and easy readable figure.

Fig. 2F. Similar to the Fig. 1A provide the size marker for the PCR detection image

Figure 3.

Fig 3A. Please provide a bit more precise description of what the graph shows. Please display the micrographs with the same magnification so that the cell morphology can be directly compared to support the claims in the manuscript text. Please indicate the graphs and photos in the figure caption (as for Fig 3B, too).

Fig3B. Phase-contrast image: what served as a control? Figure caption needs re-phrasing for better clarity.

Figs 3C and D need serious re-phrasing and description of the images step by step. Immunofluorescence images lack scale bars. Images on the Fig. 3C immunofluorescence were possibly taken at different magnifications. Was Gapdh used as a control on the Fig. 3C? Was any control displayed on the Fig 3D? These should be indicated and marked on the figure captions. Besides, for these figures the reader does not need material and method details in the figure captions. Please adjust.

Figure 4 A and B. The figure caption seriously needs better explanation why these pictures are displayed, however brief. Besides, I cannot understand how this blot supports the claim in the manuscript text. To verify against the medium (or what does DMEM stays for other than Dulbecco's Modified Eagle's Medium?) is not the convincing way, since there should be a control with other cell type. Besides, Fig. 4A contains some protein load in the left lane, which is strange and was absolutely not explained anywhere. The experiment for the figure 4 has to be performed with appropriate controls, appropriately displayed and supported by representative loading controls, or completely excluded from this manuscript.

Figure 5
Fig. 5A. Why co-stained with different antibodies? Needs explanation, which is present neither in the text, nor in the caption.

Referee #3 (Remarks):

EMM-2014-04838 comments

The study of Kamata et al addresses interesting and relevant topics on the mechanism of activation of macrophage related cell in tumor microenvironment. The study contains several novel observations, including the response of macrophages-like to the cholesterol-binding protein NPC2.

However the study contains several experimental deficiencies, especially in the data dealing with intracellular trafficking mechanisms induced by NPC2

Major comments

1. Figure 8 B. The signal for LAMP2 staining for lysosomes is too strong , the image is overexposed. The lysosomes, if stained properly, represent very clear discrete structures. The Filipin staining is also overexposed. Therefore the overlap in two signals can be an artefact, and least in part, as well as the quantification of such images. Images with better resolution and improved image acquisition have to be provided. It is also recommended to use not only LAMP2, that is frequently conserved as maker for late endosomes, by also LAMP1. Moreover, markers for other intracellular structures have to be used in parallel to prove the specify of co-localization (EEA1, TGN, Golgi)

2. Figure 8C. The authors write on the page 15, that treatment of IMC by purified bNPC facilitates cholesterol trafficking from late endosomes/lysosomes to the cytosol. However, this is insufficiently documented. The image 8C demonstrates rather reduced total intracellular amount of cholesterol after bNPC treatment. The trafficking was not analyzed here and not demonstrated. The authors have to investigate the trafficking in details, or remove such over-interpretation.

3. The analysis of the mechanism of NPC2-mediated changes in CCL6 release also contains several deficiencies and overestimations. First, Real-time PCR analysis has to be performed to examine the effect of NPC2 on the level of CCL6 transcription. Secondly the image 9D rather demonstrates decreased total level of CCL6, but not its redistribution to lysosomes in response to NPC2. By looking at morphology of CCL6-positive structures it can be assumed that it is Golgi or trans-Golgi network, and CCL6 is localized in the biosynthetic pathway. Lamp2 positive vesicles are localized close to CCL6-positve structure, but this is not real co-localization in the same vesicles. Therefore, making the conclusion about the effect of NPC2 of CCL6 translocation to lysosomes is not justified.

Moreover, targeting of newly synthesized protein to lysosomes is a very specific process, based on the interaction with Mannose-6-phospahe receptors. This process is also very specific for the lisosomal enzymes, and was never shown for cytokines. Proteins , that are transported from TGN to lysosomes are resistant to lysosomal degradation, and can be activated in lysosomes or to be released via lysosomal secretion. If proteins are delivered for the the degradation in lysosomes, that goes by the endosomal pathway. Newly synthesized protein can be rather degraded by proteosomal pathway. Using of Bafilomycin A1 for abrogation of lysosomal function is also insufficiently specific, since Bafilomycin has an effect also on other membrane compartments and can also affect exocytosis in different cell types. The effect of Bafilomycin A1 on the accumulation of CCL6 in intracellular vesicles, including lysosomes, has to be verify also by IF/confocal microscopy Therefore, the conclusion about the of NPC2-mediated mechanism affecting release of CCL6 are insufficiently justified by the experiments.

4.In the abstract is written "Studies on isolated cells ex vivo confirm that NPC2 is secreted from tumour cells and is taken up by IMCs wherein it suppresses secretion of the CCR1 ligand CC chemokine 6 (CCL6)"

However, it is not shown in the submission that NPC2 is taken up by IMCs. I was unable to find any data showing the uptake of NPC2 by IMCs. This is very important issue, since it can be that NPC2 only stimulates IMCs by interaction with some surface receptors by induction of signaling and transcriptional events, and does not affect intracellular trafficking events directly. This might also explained the fact that CCL6 levels are decreased in response to NPC2 (has to be checked by RT-PCR, as suggested above).

1st Revision - authors' response 01 May 2015

We thank all of the reviewers for the insightful comments and are pleased to see that all three reviewers agree that this is an interesting and powerful study with a number of novel findings. Each of the reviewers raises a number of recommendations to improve the manuscript and we hope we have done this in the revised version. Our specific comments to the reviewers' recommendations are indicated below.

**Referee #1 (Remarks):**

The goal of this study is to understand the role of the tumor microenvironment in early cancer progression. Particularly, the authors focus on interactions between tumor and stroma during pre-cancerous development using the oncogenic BRAF (V600EBRAF)-driven model of lung adenocarcinoma, in which pre-cancerous progression of mutant cells can be identified. The major findings of the study are as follows:

1) The authors found a population of macrophage-lineage cells (named IMCs) in the stroma of premalignant tumor, which appears to be immature based on surface marker analysis but expresses several factors, including CC chemokines (e.g. CCL6). Blockade of CC chemokine receptor 1 (CCR1) drastically reduces accumulation of IMCs and tumor burden in the V600EBRAF mice.

2) Ex vivo experiments using primary cells showed that IMCs promote proliferation and cause transcriptional changes indicating EMT consistent with the phenotypes of tumor cells co-cultured with IMCs.

3) However, a different in vivo experiment, in which the compound V600EBRAF/NPC2hypomorphic mice were analyzed, the dramatic increase of IMCs in the stroma did not increase the tumor burden. Based on this observation, the authors concluded that the IMCs "are required for maintenance of early-stage lung tumors".

4) Additional ex vivo experiments demonstrated that NPC2 secreted from tumor cells acts on the IMCs in a paracrine manner to suppress release of CCL6, which would mediate recruitment of the IMCs to the developing premalignant tumor cells (see 1).

Overall, the study addresses very important questions and suggested a population of stroma cells that may play an important role in BRAF-driven tumor progression. Importantly, the study also found novel roles of CCR1 and NPC2 signaling in regulating the tumor stroma that may influence lung cancer progression. These findings would potentially have significant impacts on developing chemoprevention strategies.

*However, a major concern about this study is that it appears to lack a conclusion at least regarding a potential role of IMCs. The conclusions from multiple experiments do not coherently lead to one larger conclusion but rather seem conflicting. It appears that the findings of 1) and 2) are somewhat contradictory to 3) and 4) as far as the role of IMCs is concerned: 1) and 2) suggest an*

*oncogenic role of IMCs while 3) and 4) the opposite. Perhaps, the term "maintenance" used several times in the text to describe the role of IMCs is vague and needs to be better defined.*

**Ans. We take on board the reviewer's comment here. We agree that Point 3 seems to be somewhat inconsistent with Points 1 and 2. The main issue here, we believe, relates to the level of amplification of IMCs in the Npc2 mutant mice, which is ~2-6 fold (Fig. 6C). While in theory this level of amplification may be expected to give rise to enhanced EMT and growth of AT2 cells in the Npc2 mutant mice, in reality there was only a trend towards increased AT2 cells (Fig. 6G), and there was no evidence for enhanced EMT or metastasis. *However, we believe these data do not contradict the overall conclusion that IMCs are protumourigenic.* Rather they can be explained by the fact that this level of amplification of IMCs is not sufficient to give a dramatic *in vivo* response and/or the tumour response and IMC quantity do not show a linear correlation in vivo. Actually, IMC accumulation is observed at the senescent stage even in *Npc2*$^{+/+}$ BVE and Braf$^{V600E}$/AdCre lungs (Fig 1,4 & 6), and this level of IMC accumulation could be sufficient for near-maximal tumour responses. We believe that abrogation of IMCs by the CCR1 inhibitor provides clear evidence for pro-tumourigenic/tumour-supportive functions of IMCs, and our conclusion that the IMCs "are required for maintenance of early-stage lung tumors" is based on our data from in vivo CCR1 inhibition, rather than those from analysis of Npc2$^{+/hypo}$ mice. We have removed the last sentence in the paragraph entitled as "NPC2 restrains IMC accumulation" in our results section to avoid any confusion, and included a sentence in the revised discussion to address this point.**

**Our view is that Point 4 raised by the reviewer does not conflict with the conclusion that IMCs are protumourigenic. Rather, this is a different point entirely, relating to the fact that AT2 cells produce a factor (NPC2) that modulates IMC recruitment to the microenvironment.**

Minor points are also related to some of the authors' descriptions and conclusions.

*a) Tumor burden is usually presented as the ratio of total tumor area to total lung area and the number of individual tumors in a given section (Fig. 5 and Fig. 7). Counting the number of AT2 cells may be less accurate because that count also include wild type AT2 cells, a majority of lung epithelial cells.*

**Ans. We chose flow cytometry to quantify tumour burden for the following reasons:**

1. **As this reviewer points out, tumour area quantification using tissue sections is the most common method to measure tumour burden in mouse models. However, one lung section (5$\mu$m thickness) represents only a small portion of total lung volume, and it is unclear if tumour area quantification in 2D-sections accurately reflects 3D-tumour volumes.**
2. **In our initial attempts to quantify tumour burden using H&E sections, we actually found high variability among different sections from the same lung tissue, suggesting potential risks of selection bias in tumour burden quantification on 2D-sections. This prompted us to develop more reliable and consistent methods.**

**(3) In addition, some tumours are complicatedly intertwined with stroma components, which sometimes made it difficult to delineate clear borders between tumours and stroma for tumour area quantification.**

**(4) We also tested CT imaging to quantify 3D tumour volumes, but resolution in μCT images was not sufficient for distinguishing tumours from stroma or non-tumour soft tissues.**

**(5) To overcome these problems, we utilised flow cytometry to directly count SPC+ and CD11c+ cells in fully digested lungs (left lobe). We found that the SPC+ cell numbers per lobe correlate well with weights of the lobes (Pearson's R=0.91). Furthermore, the sum of SPC+ and CD11c+ cell numbers per lobe show very strong correlation with the weights of the lobes (R=0.95), demonstrating that flow cytometry-based quantification is a reliable method to quantify tumour/stroma cells in tumour-bearing lungs. We included this information in supplementary Fig E6A in our revised manuscript.**

**One weakness of this method, as this reviewer mentioned, is that SPC-positivity cannot distinguish normal (non-tumour) AT2 cells from those in tumours. To estimate the potential contribution of normal AT2 cells in SPC+ cell quantification, we quantified SPC+ cell number in wild-type lungs by flow cytometry, and found 0.33 +/- 0.09 x $10^6$ SPC+ cells/lobe (average +/- SD, n=4), less than 7% of those in most of tumour-bearing lungs. Thus, more than 90% of SPC+ cells in these mice are expected to derive from tumours in these conditions, supporting the validity of this quantification strategy.**

**In contrast, contribution of normal AT2 cells in CCR1 inhibitor-treated mice could be much higher (16 - 33%), since total SPC+ cell numbers in these mice were decreased to 1.0 ~ 2.0 x $10^6$/lobe. Importantly, even though SPC+ cell numbers in these mice could be overestimated by inclusion of normal AT2 cells, they were still much lower than those in vehicle-treated mice (Fig 4F). Reduction of SPC+ cells by CCR1 inhibition might be more robust if evaluated by tumour-specific quantification methods. At least, the potential error by flow cytometry-based quantification in CCR1 inhibitor-treated mice does not alter our conclusion.**

**Nevertheless, we quantified %tumour area by the method this reviewer suggested, because it is important to confirm tumour burden by two different methods. We calculated %tumour area in right lobe sections from the mice used in Fig 4F and 6G and the data are presented in the expanded view section.**

*b) Fig. 1 does not have the legend for B.*

**Ans. We apologize for this mistake. The legend is now corrected.**

*c) Oncogene-induced senescence (OIS) is a key phenomenon during early tumor progression. The authors need to determine senescence phenotype using several standard markers, other than 'loss of Ki67, such as SA-beta-gal (Fig. 1).*

**Ans. We performed SA–βgal staining and immunoblotting (IB)/immunohistochemistry (IHC) for markers commonly used to detect senescent cells including CDK inhibitors (p16INK4a, p21CIP), p19ARF, and γH2AX (a marker for DNA-damage response, DDR), as this reviewer requested. These data are included in Fig E1 in our revised manuscript.**

*d) The experiments in Fig. 2* ***(Fig. 3?)*** *need a control, other than nothing. In general, any stomal cells when con-cultured help tumor cells for survival and/or can change growth pattern. I believe there are other cell types in the stroma, perhaps other immune cells or lung fibroblast.*

**Ans. Our aim in Fig 3 is to characterize IMCs, not to compare them with other stroma cell types. We believe that the transwell culture of AT2 cells without additional stroma cells must serve as a negative control for AT2/IMC co-culture in order to characterize IMC functions. As this reviewer commented, other types of stroma cells could show growth-promoting potentials, which may be more active than IMCs. When such growth-promoting stroma cell types are used as a negative control, IMC functions could indeed be underestimated.**

**However, it is also possible that contamination of other stroma cell type(s) into IMC cultures could cause the growth/EMT phenotype if contaminating cells have a strong growth/EMT-promoting potential. Since our method to purify IMCs involves quick (30min) adhesive selection utilizing the highly adhesive nature of IMCs, there is some risk for contamination of other adherent stroma cells such as fibroblasts, though our flow cytometry analysis confirmed that the IMC purity is usually >95%. To validate our results in Fig 3, we established lung fibroblasts from BVE lung by serial passages of total lung cell culture (new Fig E4). Using these fibroblasts, we performed transwell co-culture to compare their effects with IMCs. As expected, co-culture with lung fibroblasts increased cycling AT2 cells, but this effect was weaker than IMCs (Fig E4C), indicating that contaminating lung fibroblasts could not contribute to the growth promoting effects mediated by IMC co-culture.**

*e) The authors suggest a role of IMCs in regulating the OIS phenotype but did not show any direct evidence. Perhaps this co-culture experiment can test that idea using standard markers of senescence.*

**Ans. As this reviewer claimed, our data are not sufficient for determining whether the increase of BrdU+ AT2 cells by co-culture with IMCs (Fig 3D) does reflect escape from OIS, or represents responses of cycling AT2 cells still existing at low frequencies in largely senescent tumours (Fig 1B). We prefer the latter scenario, because lung adenomas induced by oncogenic BRAF rarely escape from OIS *in vivo* to progress into adenocarcinoma even in the presence of stroma IMCs. This cycling AT2 population could be important for the maintenance of senescent tumours, while OIS escape could be essential for the tumours to acquire infinite growth potentials and progress into malignant tumours. Accordingly, in the manuscript we do not strongly suggest direct roles of IMCs in OIS escape. To clarify this point, we revised our statements related to this issue in our discussion section.**

*f) In page 15, the title of the paragraphs does not represent the points being made below. I believe that the introduction of NPC2 and cholesterol trafficking helps understand the experiments to determine CCL6 but is not the focus.*

**Ans. We agree with this comment, and removed "promotes intracellular cholesterol trafficking" from the sub-title of this section.**

**Referee #2 (Remarks):**

The presented work explores so far understudied area of cancer research and potentially opens a new and interesting way of understanding the problem for tumor development and interaction between tumor and microenvironment, which may also have an impact on the development new treatment strategies. The suggested model seems to be adequate, however the follow up study would be necessary to understand, whether this phenomenon also takes place for further cell and cancer types. Technically, this very interesting and potentially powerful study, however, suffers from inadequate figure structure and low quality figure captions as well as extremely scarce explanations for the experiments/antibodies used etc. The text and figure captions have to be seriously edited, and adjusted to form a readable story, which is now cannot be published in this form. Besides, some data might need better verification.

Please find below the selected questions/suggestions. The whole manuscript, however, has to be improved. Please note that the experiment for the figure 4 looks very strange and has to be at least repeated and the results displayed in the different way.

Figure 1

*Fig 1: the figure 1c on the figure description obviously corresponds to 1b on the actual figure, and further on, 1c corresponds to 1d. Please correct.*

**Ans. We apologize for this mistake. The legend is now corrected.**

*Please mention briefly in the figure caption why the row of HE stainings was demonstrated along with te lung sample from AdCre+ mice similar to the manuscript text. This will promote the understanding of the figure.*

**Ans. We now explain in the legend the reason for the inclusion of the lung section of AdCre-induced tumours, as requested.**

*Fig 1B. Please include the wt control to demonstrate the basis level of Ki67 staining. Please induce all the age points in the graph along with the wt control in the graph on the right hand side. There is no mention of the number of tested animals/samples for the each age point used for Ki67 detection and analysis, which is also required to evaluate the data robustness. Please provide the appropriate numbers.*

**Ans. For %Ki67 quantification in the tumours, we analysed lung sections from total 20 BVE mice at ages from 18 to 100 days, and a total of 3613 – 37505 tumour cells per each mouse (average 16423 tumour cells/mouse) were evaluated for Ki67 positivity. As this reviewer suggested, we categorized these animals into 4 groups (<4wk, 4-6wk, 6-8wk, >8wk, n=4 to 6 for each group)**

**and presented %Ki67 as an average +/- SD of each group in the graph. Based on this quantitative analysis, we prepared new IHC images representative for the averaged %Ki67 in each group.**

**Unfortunately, we found technical difficulties to quantify %Ki67 in the wt lung AT2 cells (normal counterpart of tumour cells) because of the rarity of AT2 cells in wt lungs and difficulty to identify AT2 cells on the wt sections counterstained with hematoxylin only. The aim of this analysis is to quantitatively confirm the dynamic reduction of Ki67 positivity in tumours during the time course, and we believe %Ki67 data in tumours are sufficient for this purpose.**

*Fig. 1C. The data display will be significantly enhanced when the Mac2 staining will be demonstrated on the section adjacent to the HE one, also showing both magnifications, which were used for the HE. In this way the morphological characteristics of the cells mentioned in the manuscript text can be compared to and supported by the Mac 2 staining.*

**Ans. We prepared H&E and Mac2 IHC using serial sections, and imaged at x100 and x400 magnifications (see new Fig 1D).**

Figure S1.

*Fig. 1A. A sentence explaining the purpose of the cell isolation would significantly add to the readability.*

**Ans. We revised the legend to describe the purpose to develop this method.**

*Fig. S1B. It makes more sense to display similar magnification (-s) of the phase-contrast micrographs for the different cell isolates. When it is not quite representative, it might be helpful to include 2 magnifications for each cell type.*

**Ans. We prepared images at the same magnifications (x200) (see new Fig. E2B).**

*Fig. S1C. Why the Fr-2 cells were displayed? There is also no explanation in the text of the manuscript as I could found. Please provide brief description and reference for the Papanicolau stain, and what this stain is for.*
*Fig. S1D. Why was SP-C staining included? What does it show?*

**Ans. We initially named 3 populations fractionated from BVE lung as Fr-1 (IMCs), Fr-2 (AT2 cells) and Fr-3 (tumour cells), but these descriptions were removed from Fig E1A in our submitted manuscript (revised Fig E2A) during our final packaging of this manuscript without appropriately editing the legends for Fig E1C/D. We apologize for any confusion due to inappropriate naming of the isolated fractions. We revised the legends for new Fig E2C/D without using the term "Fr-2."**

**AT2 cells are characterized by lamellar bodies containing pulmonary surfactants that can be visualized by the Papanicolau staining. SPC is one of the major surfactants AT2 cells express, and**

**has been used as a specific marker for AT2 cells. SPC and Papanicolau staining were performed to confirm enrichment for AT2 cells. We added more description about the Fr-2/AT2 cells in our revised text and figure legends.**

Figure 2.

*Generally is quite advantageous to follow the story briefly by the figure captions, which also helps the readership to understand the authors' ideas. Please include very brief description of why you placed the giemsa cell stain also on the figure 2A caption. The same for Fig 2C, very brief mentioning for the purpose of the stain, as for Fig. 2D and 2E.*

**Ans. We revised the figure legend as requested, with concisely describing the purpose of each panel.**

*Fig. 2D. Would be really helpful to display the same cells for phase-contrast and giemsa stain, which is I hope not restrictively hard to achieve, but significantly enhance the convincing power of the images. Please also indicate, "phase-contrast" as you indicated giemsa on appropriate images to perform a uniform and easy readable figure.*

**Ans. We prepared a phase-contrast image and a Giemsa image using IMC culture for 2 weeks, with indicating "phase-contrast" and ""Giemsa" as this reviewer requested (see new Fig. 2D).**

*Fig. 2F. Similar to the Fig. 1A provide the size marker for the PCR detection image*

**Ans. We prepared BRAF recombination PCR images with size markers. WT/Lox alleles and an LSL allele were amplified using different primer pairs.**

Figure 3.

Fig 3A.
*Please provide a bit more precise description of what the graph shows.*

**Ans. We added precise descriptions in the legend for Fig 3A.**

*Please display the micrographs with the same magnification so that the cell morphology can be directly compared to support the claims in the manuscript text.*

**Ans. These photos (Fig 3A, left and middle) are at the same magnification as indicated with scale bars. In standard cultures, CMT64 cells develop typical epithelial clusters, in which the cells are tightly packed. In contrast, in the presence of IMC-CM, CMT64 cells showed a more disperse distribution, some of which lost cell-to-cell contacts. These cells showed a more spread morphology, showing relatively larger cell sizes when examined by a phase-contrast microscope. This is also the case in confocal images in Fig 3D for primary AT2 cells treated with IMC-CM, showing flattened morphology with relatively larger sizes than untreated AT2 cells forming tightly packed clusters.**

*Please indicate the graphs and photos in the figure caption (as for Fig 3B, too).*
**Ans. We again apologize these mistakes. We revised the legends for Fig 3A/B to clearly indicate each graph/photo.**

*Fig3B.*
*Phase-contrast image: what served as a control? Figure caption needs re-phrasing for better clarity.*

**Ans. An image of CMT64 cells in standard culture (DMEM + 10%FCS) is displayed as a control. We described this in the figure legend.**

*Figs 3C and D need serious re-phrasing and description of the images step by step.*

*Immunofluorescence images lack scale bars.*
**Ans. We added scale bars in confocal images in Fig. 3D.**

Was Gapdh used as a control on the Fig. 3C?
**Ans. Yes, we described the purpose of Gapdh RT-PCR as a control in the figure legend.**

*Was any control displayed on the Fig 3D?*

**Ans. Yes, primary AT2 cells cultured in standard media (DMEM + 10%FCS) labelled as “AT2 w/o IMC-CM” serve as a control to show the effects of IMC-CM added in the AT2 culture (shown in the right, labelled as “AT2 with IMC-CM”). We described this in the figure legend.**

*Images on the Fig. 3C* ***(3D?)*** *immunofluorescence were possibly taken at different magnifications.*

**Ans. Two images in the left were both taken with 60x objective, and processed using Image J and Huygens deconvolution software in an entirely identical way. Possible reasons for the cell size difference between them are described above. The right image was 3x enlarged (3x zoom) using the zoom function equipped in FV1000 confocal acquisition software when this image was taken, but not cropped after image acquisition. This image highlights a dividing vimentin+ cell (in cytokinesis) with internalized E-cadhein, as explained in our text.**

*These should be indicated and marked on the figure captions. Besides, for these figures the reader does not need material and method details in the figure captions. Please adjust.*

**Ans. We revised the legend in Fig 3 as above.**

Figure 4 A and B.

*The figure caption seriously needs better explanation why these pictures are displayed, however brief. Besides, I cannot understand how this blot supports the claim in the manuscript text.*

**Ans. We moved this figure to supplementary Fig E5 in our revised manuscript (see below). Aims of these experiments were described in the expanded view section. We initially included this figure to confirm that IMCs secrete substantial amounts of PDGFA/TGFb/CCL6. Given the highly sensitive nature of mass-spec detection of peptides, we believe that confirming secreted proteins by other methods (e.g. immunoblotting) is still useful.**

*To verify against the medium (or what does DMEM stays for other than Dulbecco's Modified Eagle's Medium?) is not the convincing way, since there should be a control with other cell type. Besides, Fig. 4A contains some protein load in the left lane, which is strange and was absolutely not explained anywhere. The experiment for the figure 4 has to be performed with appropriate controls, appropriately displayed and supported by representative loading controls, or completely excluded from this manuscript.*

**Ans. This analysis did not aim to compare the amounts of the proteins secreted by IMCs with those by other cell types. However, the culture media used for previous Fig 4A contains 10%FCS, and the level of PDGFA & TGFβ within this media (10%FCS) is unknown. To exclude the possibility that PDGFA and TGFβ detected in IMC-CM derives from FCS, DMEM (Dulbecco's modified Eagle medium) containing 10%FCS (same % as IMC-CM) was loaded to confirm background levels. The proteins seen in the left lane of the right panel in previous Fig 4A (showing protein loading) derive from FCS.**

**In the revised version we established lung fibroblasts (new Fig E4) from tumour-bearing lungs to compare secreted protein levels between IMCs and lung fibroblasts as this reviewer requested. To avoid any confusion, we collected conditioned media in serum-free culture conditions for CCL6 and TGFβ detection. Our preliminary analyses confirm that CCL7 is secreted mainly by lung fibroblasts but its secretion levels by IMCs are relatively low. Therefore, CCL7 immunoblotting was excluded from the figure. Unfortunately, PDGFA was undetectable in serum-free conditioned media of IMCs, which could be due to the possible requirement of serum factors to induce PDGFA expression, and/or to stabilize secreted PDGFA. So the PDGFA blot remains in the form we initially submitted.**

**We partially agree with this reviewer's suggestion to completely exclude this figure, but also find some merits as described above. So we decided to move this figure to the expanded view section (new Fig E5)**

Figure 5.

*Fig. 5A. Why co-stained with different antibodies? Needs explanation, which is present neither in the text, nor in the caption.*

**Ans. The purpose of dual staining is to unambiguously confirm Mac2+ IMCs and Ecad+ AT2 cells. Also, since Mac2 and Ecad are cell surface (plasma membrane) proteins, dual staining confirms CCR1 localization at the plasma membrane. CCR1 must be at the plasma membrane to function**

**as a cell surface receptor, so it is useful to show its membrane localization. We can find substantial amounts of intracellular CCR1 in IMCs, consistent with a previous publication describing that CCR1 is not efficiently recycled when internalized (Elsner et al, Allergy 60: 1386-1393). We described these in our revised manuscript text and figure legends.**

Referee #3 (Remarks):

EMM-2014-04838 comments

The study of Kamata et al addresses interesting and relevant topics on the mechanism of activation of macrophage related cell in tumor microenvironment. The study contains several novel observations, including the response of macrophages-like to the cholesterol-binding protein NPC2. However the study contains several experimental deficiencies, especially in the data dealing with intracellular trafficking mechanisms induced by NPC2.

Major comments

*1. Figure 8 B. The signal for LAMP2 staining for lysosomes is too strong , the image is overexposed. The lysosomes, if stained properly, represent very clear discrete structures. The Filipin staining is also overexposed. Therefore the overlap in two signals can be an artefact, and least in part, as well as the quantification of such images. Images with better resolution and improved image acquisition have to be provided. It is also recommended to use not only LAMP2, that is frequently conserved as maker for late endosomes, by also LAMP1. Moreover, markers for other intracellular structures have to be used in parallel to prove the specify of co-localization (EEA1, TGN, Golgi)*

**Ans. As shown in revised Fig 7A/C (previous Fig 8A/C), extremely high filipin staining (categorized as 3+ in our quantitative analysis) was observed in 10-15% of Npc2$^{hypo/+}$ IMCs, but rarely in Npc2$^{+/+}$ IMCs. As this reviewer claims, filipin staining in these Npc2$^{hypo/+}$ IMCs containing very high levels of free cholesterol seems to be overexposed, but this level of exposure was also needed to distinguish weak vesicular filipin staining (categorized as 1+ in our quantitative assay) from negative vesicular staining (categorized as 0). We set the exposure condition using Npc2$^{+/+}$ IMCs, and the same condition was applied to Npc2$^{hypo/+}$ IMCs. When we set the exposure conditions to those used for Npc2$^{hypo/+}$ IMCs to avoid overexposure, image resolution was not sufficient for accurately categorizing low level staining, resulting in inaccurate quantification. Therefore, we believe that our imaging condition is optimal for quantitative analyses, even though some Npc2$^{hypo/+}$ IMCs show extremely high filipin staining with saturated fluorescence intensities.**

**However, we agree with this reviewer's criticism for LAMP2/filipin dual staining of Npc2$^{hypo/+}$ IMCs (in previous Fig 8B, revised Fig 7B), especially for LAMP2 immunofluorescence. The aim of this panel was to show if filipin staining (free cholesterol) could be associated with LAMP2+ vesicles, but not to perform quantitative comparisons. To accurately determine LAMP2+**

**structures, exposure levels must be controlled. We therefore revised LAMP2/filipin staining using confocal imaging with appropriately adjusting exposure conditions to avoid fluorescence signal saturation, and also filipin-stained IMCs were co-stained for LAMP1, EEA1, TGN46 and Giantin. Through these experiments, we obtained some new findings that are now incorporated in Fig 7B, E10 & E11. These findings are summarized as following:**

**(1) Coarse structures strongly stained with filipin are included in LAMP2+ large vesicles (revised Fig 7B) that are mostly devoid of LAMP1/EEA1/TGN46/Giantin staining (Fig E10), suggesting that free cholesterol does not accumulate at LAMP1+ late endosomes/lysosomes or other cellular organelles in Npc2$^{hypo/+}$ IMCs.**

**(2) These coarse structures are observed near ventral surfaces of the IMCs contacting with extracellular substrata, distinct from the main LAMP2 distribution at the dorsal side (Fig E11A).**

**(3) The filipin-positive structures are partially associated with vertical F-actin staining suggestive of podosomes (Fig E11B).**

**Based on these observations, we conclude that LAMP2 is not a specific marker for LE/Ly in IMCs, and LAMP1 is more suitable for imaging studies for CCL6 localization at vesicular lysosomes as this reviewer suggested (see below).**

*2. Figure 8C. The authors write on the page 15, that treatment of IMC by purified bNPC facilitates cholesterol trafficking from late endosomes/lysosomes to the cytosol. However, this is insufficiently documented. The image 8C demonstrates rather reduced total intracellular amount of cholesterol after bNPC treatment. The trafficking was not analyzed here and not demonstrated. The authors have to investigate the trafficking in details, or remove such over-interpretation.*

**Ans. We agree with this comment, so we removed this statement.**

3. The analysis of the mechanism of NPC2-mediated changes in CCL6 release also contains several deficiencies and overestimations.

*First, Real-time PCR analysis has to be performed to examine the effect of NPC2 on the level of CCL6 transcription.*

**Ans. We performed CCL6 qRT-PCR to quantitatively confirm the level of Ccl6 transcription, and confirmed that CCL6 transcription in bNPC2-treated IMCs is almost equivalent to that in untreated IMCs (revised Fig 8C).**

*Secondly the image 9D rather demonstrates decreased total level of CCL6, but not its redistribution to lysosomes in response to NPC2. By looking at morphology of CCL6-positive structures it can be assumed that it is Golgi or trans-Golgi network, and CCL6 is localized in the biosynthetic pathway. Lamp2 positive vesicles are localized close to CCL6-positve structure, but this is not real co-localization in the same vesicles. Therefore, making the conclusion about the effect of NPC2 of CCL6 translocation to lysosomes is not justified.*

**Ans. As this reviewer points out, CCL6-positive peri-nuclear structures in untreated (-bNPC2) IMCs show a morphology suggestive of the Golgi apparatus. We confirmed that peri-nuclear CCL6+ structures in freshly isolated IMCs are indeed positive for a Golgi marker Giantin. Interestingly, TGN46 (trans-Golgi network marker) staining was detected predominantly in cytoplasmic vesicles in IMCs in stark contrast to peri-nuclear TGN46 staining in MEFs, which may represent secretory vesicles actively shuttling to the plasma membrane in IMCs. CCL6 was not detected at the TGN46+ cytoplasmic vesicles, suggesting that CCL6 may not utilize conventional secretory vesicles for its secretion. Although these data are not directly relevant to our manuscript, we believe that they are useful for this reviewer to interpret our manuscript and understand our working hypothesis. Therefore, we present these data as "Reviewer-only Figure 1" attached to the end of this letter.**

**In contrast, peri-nuclear CCL6 staining in bNPC2-treated IMCs (+bNPC2) is much weaker and shows a morphology distinct from those in untreated IMCs. Since this peri-nuclear, vesicular CCL6 staining in bNPC2-treated IMCs partially co-localizes with LAMP2 (in our initial submission), we assumed that CCL6 could translocate to (peri-nuclear) lysosomes in IMCs when treated with exogenous NPC2. To confirm the nature of these vesicles in bNPC2-treated IMCs, we performed co-staining for CCL6 and LAMP1/Golgi markers (revised Fig 8D & Fig E12). Similar to LAMP2, LAMP1 partially co-localizes with CCL6 at peri-nuclear (and some peripheral) vesicles in bNPC2-treated IMCs. Since LAMP2 staining could be associated with not only LE/Ly but also free cholesterol-rich structures described above, we replaced our original LAMP2/CCL6 immunofluorescence images with new LAMP1/CCL6 images (new Fig 8D).**

**As this reviewer indicates, some CCL6 staining in bNPC2-treated IMCs was not co-localized with LAMP1, which likely represents CCL6 in the biosynthetic pathway. Indeed, CCL6/Golgi marker dual staining confirmed co-localization of CCL6 with TGN46 and Giantin at the peri-nuclear regions in the bNPC2-treated IMCs (new Fig E12). However, CCL6+TGN46- and CCL6+Giantin-vesicles were also detected (new Fig E12), suggesting that CCL6 could distribute in two different compartments, the biosynthetic (Golgi) and degradation (lysosome) pathways, in bNPC2-treated IMCs.**

*Moreover, targeting of newly synthesized protein to lysosomes is a very specific process, based on the interaction with Mannose-6-phospahe receptors. This process is also very specific for the lisosomal enzymes, and was never shown for cytokines. Proteins, that are transported from TGN to lysosomes are resistant to lysosomal degradation, and can be activated in lysosomes or to be released via lysosomal secretion. If proteins are delivered for the the degradation in lysosomes, that goes by the endosomal pathway.*

**Ans. We would like to thank this reviewer for providing these thoughtful suggestions. With regard to these questions, we sought to clarify the secretory route(s) for CCL6. Because the majority of CCL6 in freshly isolated IMCs locates at the Golgi (Reviewer-only Figure 1), we tracked CCL6 trafficking from the Golgi by confocal imaging after short (3h) culture of IMCs with or without bNPC2. Since we found that CCL6 secretion is dependent on extracellular calcium, we utilized calcium-free media during the 3h culture to minimize endosomal CCL6 distribution**

**caused by re-uptake (endocytosis) of once secreted CCL6. Although these new data are suggestive of CCL6 trafficking through the endosomal pathway rather than direct transfer from the Golgi to the lysosomes, we failed to clearly identify the route through which CCL6 translocates to the lysosomes. We do not think these data are conclusive enough for publication, but they may help this reviewer understand the potential mechanism by which CCL6 translocates to the lysosomes. Therefore, we decided to present these data as "Reviewer-only Figure 2." Our new data regarding intracellular CCL6 trafficking are summarized as following:**

**(1) In control culture without exogenous NPC2 addition, we found that CCL6 is initially delivered to recycling endosomes (RE), suggesting that the endosomal pathway could potentially serve as a route for CCL6 secretion as reported for other cytokines in macrophages (Murray & Stow, Front Immunol 5: 538).**

**(2) In contrast, CCL6 localization in RE was acutely (within 3h treatment) abrogated by exogenous NPC2 treatment. CCL6 staining in this condition showed vesicular distribution, but these CCL6+ vesicles were negative for an early endosome marker EEA1 or a lysosome marker LAMP1.**

**Based on these observations, we hypothesize that in the presence of exogenous NPC2, CCL6 initially transferred to RE could first translocate to yet-to-be characterized vesicular structures before being targeted to the lysosomes. Although we speculate that CCL6+ vesicles in NPC2-treated IMCs may belong to EEA1/LAMP1-negative endosomal compartments, further studies with expertise will be needed to prove this. We hope this reviewer can understand that a detailed characterization of the CCL6+ vesicles is beyond the scope of our revise work along with the limited timescale.**

*Newly synthesized protein can be rather degraded by proteosomal pathway. Using of Bafilomycin A1 for abrogation of lysosomal function is also insufficiently specific, since Bafilomycin has an effect also on other membrane compartments and can also affect exocytosis in different cell types. The effect of Bafilomycin A1 on the accumulation of CCL6 in intracellular vesicles, including lysosomes, has to be verify also by IF/confocal microscopy*

**Ans. CCL6 degradation in lysosomes must be the major technical barrier against clear and robust detection of CCL6/LAMP1 co-localization. Since chemical inhibition of lysosomes could overcome this limitation, we performed CCL6/LAMP1 dual staining using IMCs loaded with bNPC2 followed by 24hr Bafilomycin-A1 treatment, and found clear CCL6/LAMP1 co-localization in this condition (new Fig E13), These data, together with immunoblot data in Fig 8E, support our claim for lysosomal translocation and degradation of CCL6. As this reviewer mentioned, Bafilomycin A1 is known to induce lysosome exocytosis in macrophages (Tapper, J Leuk Biol 59: 613-622) that may contribute to the increase of secreted CCL6 by Bafilomycin A1 treatment in bNPC2-unloaded IMCs (Fig 8E), but accumulation of intracellular CCL6 cannot be explained by this mechanism. Furthermore, CCL6 secretion by exogenous NPC2-treated IMCs is not increased by Bafilomycin A1 treatment (Fig 8E), suggesting that Bafilomycin A1-induced exocytosis of peripheral lysosomes does not contribute to CCL6 secretion by NPC2-treated IMCs.**

*Therefore, the conclusion about the of NPC2-mediated mechanism affecting release of CCL6 are insufficiently justified by the experiments.*

**Overall, our new data are consistent with our claim that exogenous NPC2 triggers CCL6 translocation to lysosomes for degradation, though it remains to be clarified if this is due to CCL6 trafficking through the endosomal pathway.**

*4. In the abstract is written "Studies on isolated cells ex vivo confirm that NPC2 is secreted from tumour cells and is taken up by IMCs wherein it suppresses secretion of the CCR1 ligand CC chemokine 6 (CCL6)" However, it is not shown in the submission that NPC2 is taken up by IMCs. I was unable to find any data showing the uptake of NPC2 by IMCs. This is very important issue, since it can be that NPC2 only stimulates IMCs by interaction with some surface receptors by induction of signaling and transcriptional events, and does not affect intracellular trafficking events directly. This might also explained the fact that CCL6 levels are decreased in response to NPC2 (has to be checked by RT-PCR, as suggested above).*

**Ans. We measured Npc2 mRNA in IMCs cultured with bNPC2 by qRT-PCR. Npc2 transcription was only marginally increased by exogenous NPC2 treatment (revised Fig 5H), and did not reach statistically significant levels. Thus, transcriptional up-regulation is unlikely to be the major cause for the intracellular NPC2 accumulation in IMCs treated with exogenous NPC2.**

**To unambiguously confirm NPC2 uptake by IMCs, we treated freshly isolated IMCs with 82.5nM recombinant NPC2 protein conjugated with Alexa488 (Huang et al, PLoS One 9: e88893) for 2h, followed by 2h chase in NPC2-free media. Robust uptake of NPC2-Alexa488 was confirmed by live confocal imaging (revised Fig 5G). Interestingly, NPC2-Alexa 488 incorporated by IMCs showed not only lysosome-like fine vesicular distribution but also reticular structures surrounding coarse vesicular puncta observed in the DIC image (zoomed images in Fig 5G). The latter distribution is reminiscent of LAMP2 staining shown in Fig 7B, suggesting that NPC2 uptaken by IMCs could be transferred from LE/Ly to the coarse vesicular structures associated with LAMP2.**

**Other points (not mentioned by reviewers)**

**(1) We found a mis-calculation of log-rank p-values for the survival data presented in Fig 6A (p-values not indicated in the figure, but described in the text). We corrected these in our revised manuscript (in the text).**

**(2) We found inappropriate descriptions for our statistical analyses in the original submission (in Materials and Methods). We revised these statements in our revised manuscript ("Statistics" section in Material and Methods). Of note, we revised all t-tests using Welch's t-test, so those calculated by student's t-test in our previous figures were all corrected.**

Reviewer-only Figure 1

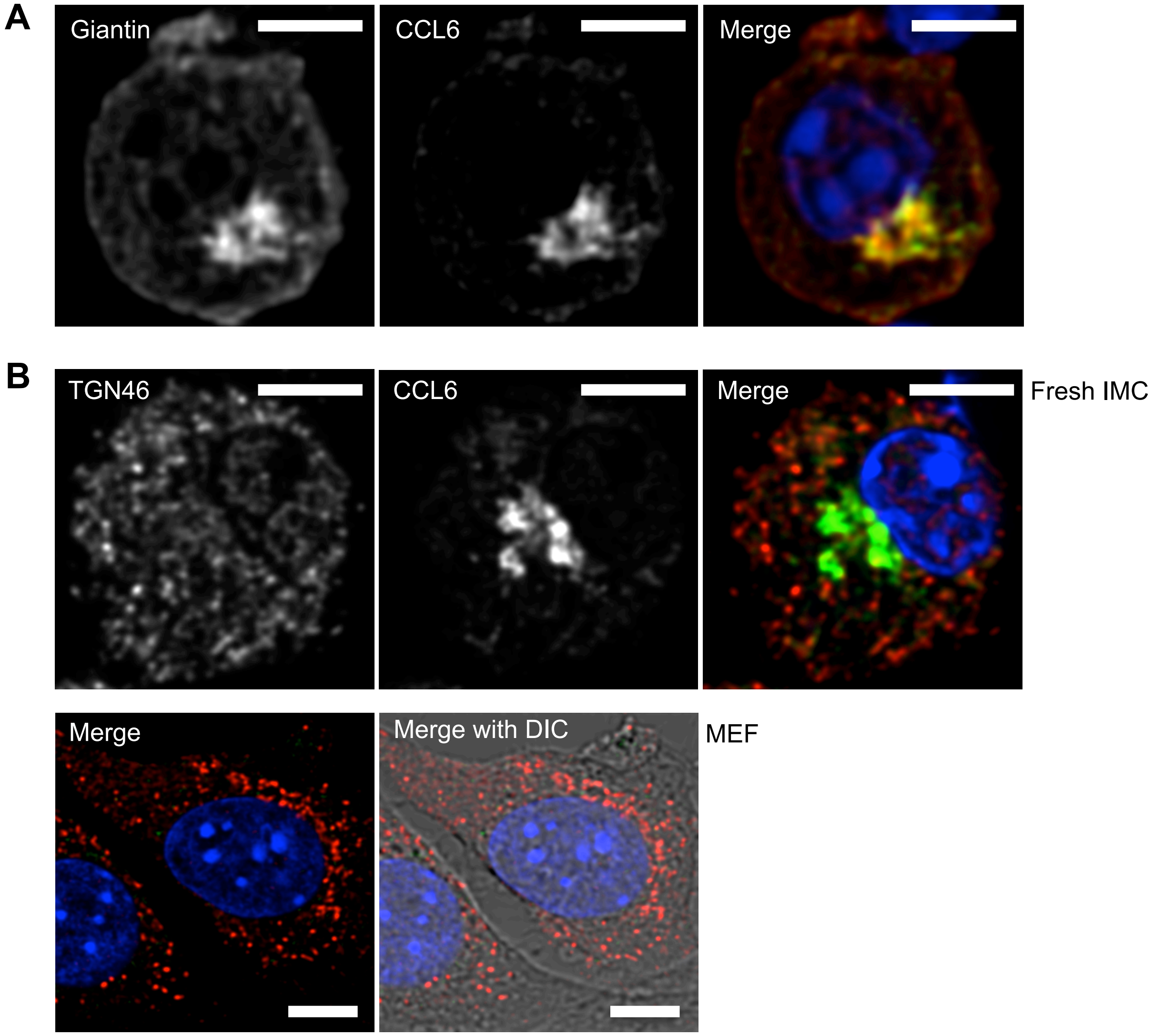


**Reviewer-only Figure 1. CCL6 and organelle marker staining of freshly isolated *Npc2*$^{+/+}$ IMCs**

A. IMCs freshly isolated from *Npc2*$^{+/+}$ BVE mice were immunostained for Giantin and CCL6, and imaged by CLSM. Scale bars, 5μm. Clear co-localization of CCL6 and Giantin was observed at perinuclear structures suggestive of the Golgi apparatus.

B. Npc2$^{+/+}$ IMCs (top panels) and immortalized MEFs (bottom panels) were stained for TGN46 and CCL6, and imaged by CLSM. Single-color greyscale images for CCL6 and TGN alongside with a merged color image are indicated for IMCs (top panels), while merged images with or without DIC are shown for MEFs (bottom panels). Scale bars, 5μm. TGN46 staining shows cytoplasmic vesicular signals but minimal co-localization with perinuclear CCL6 staining. TGN46 staining in MEFs shows typical perinuclear staining for TGN46 and negative staining for CCL6, demonstrating the staining specificity.

Reviewer-only Figure 2

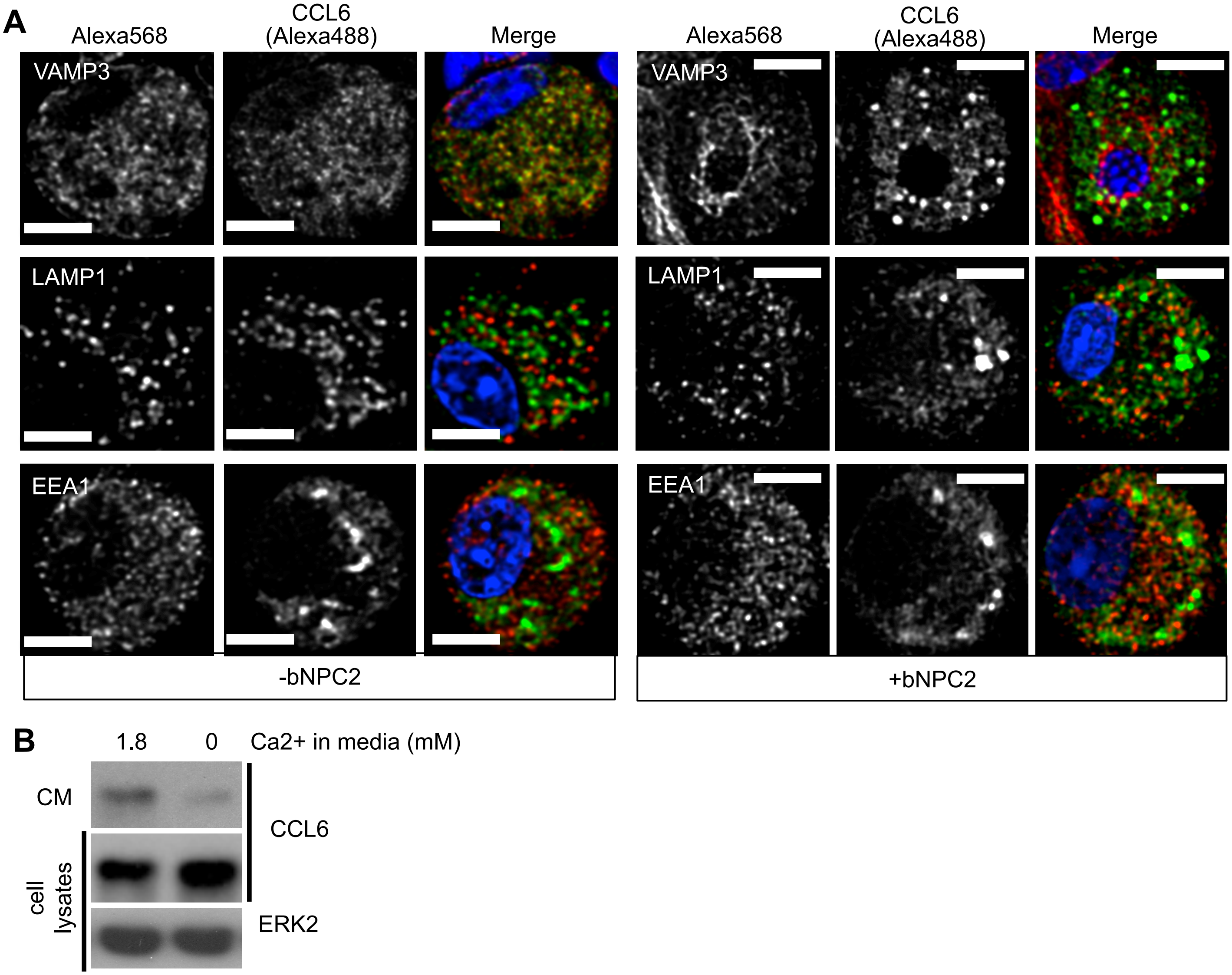


**Reviewer-only Figure 2. CCL6 localisation in IMCs is altered by short-term NPC2 treatment**

A. To follow intracellular CCL6 trafficking from the Golgi in IMCs, freshly isolated *Npc2*$^{+/+}$ IMCs, in which CCL6 mostly resides in the Golgi (see Reviewer-only Figure 1), were cultured for 3hrs in serum-free, calcium-free DMEM with or without 50μg/ml bNPC2, immunostained for CCL6 and endo-lysosome markers (VAMP3/LAMP1/EEA1), and imaged by CLSM. Scale bars, 5μm. Calcium-free media was used to minimize CCL6 secretion (see below B), since secreted CCL6 could be endocytosed and delivered to endo-lysosomes, which potentially causes mis-interpretation of the data. In the absence of exogenous NPC2 (-bNPC2, left panels), fine vesicular or reticular CCL6 staining partially co-localized with VAMP3 (recycling endosome marker), indicating that CCL6 is delivered from the Golgi to the periphery through recycling endosomes in the absence of bNPC2. In contrast, in the presence of bNPC2 (+bNPC2, right panels), CCL6 staining was detected as relatively larger vesicles not co-stained with VAMP3, LAMP1 or EEA1.

B. Immunoblot analysis of CCL6 secretion by IMCs cultured in calcium-free media for 24h. In calcium-free media, CCL6 secretion into CM was clearly decreased, whereas intracellular CCL6 protein levels were increased.

2nd Editorial Decision 03 June 2015

Thank you for the submission of your revised manuscript to EMBO Molecular Medicine. We have now received the enclosed reports from the referees that were asked to re-assess it. As you will see the reviewers are now globally supportive, albeit with a few remaining minor issues and I am pleased to inform you that we will be able to accept your manuscript pending the following final amendments:

1) I invite you to adhere to Reviewer 1's requests to tone-down your conclusions and to clarify the sentence mentioned. Please also deal with Reviewer 2's requests to improve presentation of Figure 2. These and the following amendments will be dealt with editorially.

2) On a related note, I would also ask you to make sure the frames indicating magnified insets are precisely positioned and sized to reflect the actual magnifications (see Fig. 6F).

3) Please provide all figures as separate files and the manuscript as a Word file.

4) Could you please state the gender of the mice used in the experiments? Please also mention their age in the Materials and Methods section (the latter in addition to the figure legends, which you have already done).

5) We are now encouraging the publication of source data, particularly for electrophoretic gels and blots, with the aim of making primary data more accessible and transparent to the reader. Would you be willing to provide a PDF file per figure that contains the original, uncropped and unprocessed scans of all or at least the key gels used in the manuscript? The PDF files should be labeled with the appropriate figure/panel number, and should have molecular weight markers; further annotation may be useful but is not essential. The PDF files will be published online with the article as supplementary "Source Data" files. If you have any questions regarding this just contact me.

6) Every published paper now includes a 'Synopsis' to further enhance discoverability. Synopses are displayed on the journal webpage and are freely accessible to all readers. They include a short standfirst as well as 2-5 one sentence bullet points that summarise the paper. Please provide the synopsis including the short list of bullet points that summarise the key NEW findings. The bullet points should be designed to be complementary to the abstract - i.e. not repeat the same text. We encourage inclusion of key acronyms and quantitative information. Please use the passive voice. Please attach this information in a separate file or send them by email, we will incorporate it accordingly. You are also welcome to suggest a striking image or visual abstract to illustrate your article. If you do please provide a jpeg file 550 px-wide x 400-px high.

Please submit your revised manuscript within two weeks.

I look forward to reading the final evised version of your manuscript as soon as possible.

***** Reviewer's comments *****

Referee #1 (Comments on Novelty/Model System):

The mouse model recapitulates a tumor initiating tumor mutation discovered in a subset of human lung cancers and the pathological features. Thus it is relevant.

Referee #1 (Remarks):

In this revised manuscript, the authors try to respond thoroughly to the comments from me and the others. (The other reviewers were very thoughtful and constructive.)

The manuscript has been significantly improved with additional data and better description.

However, the authors could tone down their specific conclusions and rather speculate them broadly (considering alternative explanations). Although I am not completely persuaded by the authors argument, their findings may be published to be discussed/debated by a broader audience.

Upon editorial revision, I would like to see that the authors better define 'maintenance of early stage-tumors'. Do they mean survival or prevent progression?

Referee #2 (Comments on Novelty/Model System):

The presented work deals with interesting and high impact area of cancer research and potentially opens a new way of understanding tumor development and tumor - microenvironment interaction, which may affect development of new treatment strategies. The suggested model seems to be adequate. In the revised article, the authors made a good job answering to the comments and suggestions, which, hopefully would significantly enhance the data robustness and persuasiveness of the manuscript.

Referee #2 (Remarks):

There are two minor comments concerning Figure 2 of the manuscript
Figure 2D: The phase-contrast and Giemsa-stained images were indeed marked as requested. However, they do have different magnification (which is to be understood from different scale bars), and therefore itis difficult to see, whether they belong to the same sample or not. This discrepancy was not mentioned or explained.

Figure 2F: The idea to display the size marker was to identify, whether the PCR results are of the same size. The marker fragment sizes were not indicated, however. There is also no indication in the legend. This should be corrected similar to the Figure E5 B and C

Referee #3 (Remarks):

The authors properely addressed all my comments. The quolity of IF illustrations and biological signficance of the new provided data is clearly impooved

2nd Revision - authors' response 16 June 2015

Many thanks for the email regarding acceptance of our manuscript in your pending final amendments.

We have addressed the six points you raised in your email as follows:

Point 1.

a) *I invite you to adhere to Reviewer 1's requests to tone-down your conclusions and to clarify the sentence mentioned.*

In the revised manuscript we have altered the beginning of paragraph 3 in the Discussion to address the concerns of Reviewer 1 with regard to the role of IMCs.

Reviewer 1 would also like a better definition of “maintenance of early stage tumours”, asking whether this means survival or progression. This refers to the situation in which we have treated BVE mice with a CCR1 inhibitor (Figure 4) and find that there is a substantial reduction in IMC and AT2 cell numbers following inhibitor treatment for 4 weeks. Since we know that CCR1 is predominantly expressed on IMCs and not AT2 cells, our suggestion is that the inhibitor targets IMCs and this has the secondary effect of reducing AT2 cell number. Thus, we conclude that IMCs are required for tumour maintenance. We prefer to use the term “maintenance” rather than “survival” or “progression” for the following reasons: The term “progression” is not relevant in this context since we are not assessing the further development of tumours. The term “survival” is also not accurate because we cannot distinguish between alterations in survival of the tumour cells (due to apoptosis/necrosis) and immune surveillance. In all, we think the term maintenance is most appropriate. We hope you agree.

*b) Please also deal with Reviewer 2's requests to improve presentation of Figure 2.*

Regarding Fig 2D, we think there was some misunderstanding, causing some. For this panel, IMCs were cultured on tissue culture plates for 2 weeks, and phase-contrast images were taken with 40x objective. Then the cells were trypsinised and smeared on glass slides for Giemsa staining. The image for Giemsa-stained cells was taken with 100x oil-immersion objective. Therefore, we cannot take phase-contrast and Giemsa images at the entirely same condition. Since the Giemsa-stained cells were prepared by trypsinisation, their morphology was not identical to that in the phase-contrast image of the cells in culture. Microscopic observation using 100x oil-immersion objective is a standard method to investigate detailed morphological characteristics of Giemsa-stained hematopoietic cells, and we do not think it is reasonable to use lower magnification (same as phase-contrast imaging) for imaging of the Giemsa-stained cells. We added some descriptions in Fig 2D legend to explain our methodology for these images.

Fig 2F was fixed as Reviewer-2 requested.

Point 2.

*I would also ask you to make sure the frames indicating magnified insets are precisely positioned and sized to reflect the actual magnifications (see Fig. 6F).*

Thank you for realising this - this has now been adjusted.

Point 3

*Please provide all figures as separate files and the manuscript as a Word file.*

This is done

Point 4

*Could you please state the gender of the mice used in the experiments? Please also mention their age in the Materials and Methods section.*

This is done

Point 5

*We are now encouraging the publication of source data, particularly for electrophoretic gels and blots, with the aim of making primary data more accessible and transparent to the reader. Would you be willing to provide a PDF file per figure that contains the original, uncropped and unprocessed scans of all or at least the key gels used in the manuscript?*

We now enclose an Excel file with raw data for all of the graphs. We also include a PDF file with the original, uncropped and unprocessed scans of the figures. However, please note that unfortunately we could not fund the original photograph for the Col1a1 RT-PCR data presented in Figure 3c since the computer upon which this was stored broke down and we could not retrieve the file. We also could not find the loading control for Figure 5F unfortunately.

Point 6

*Every published paper now includes a 'Synopsis' to further enhance discoverability. Synopses are displayed on the journal webpage and are freely accessible to all readers. They include a short standfirst as well as 2-5 one sentence bullet points that summarise the paper. Please provide the synopsis including the short list of bullet points that summarise the key NEW findings.*

A synopsis is now included.

We hope you are satisfied with the above and look forward to hearing a final decision.
