# Supplementary figures and images for: The cholesterol-binding protein NPC2 restrains recruitment of stromal macrophage-lineage cells to early-stage lung tumours
Source: EMBO Mol Med. 2015 Jul 16;7(9):1119–37. doi: 10.15252/emmm.201404838 (PMC4568947; doi:10.15252/emmm.201404838)

Figure 1A

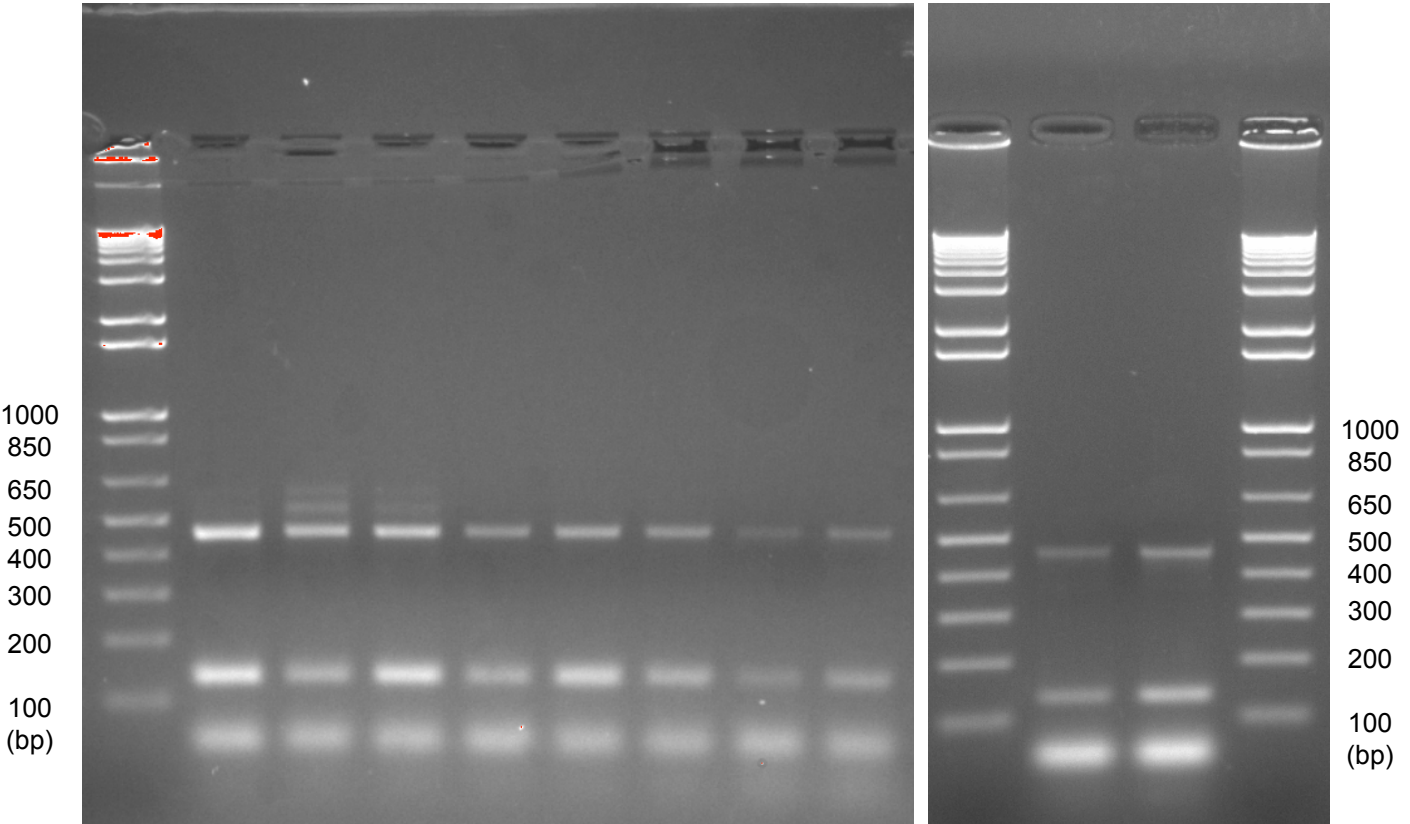

Supplement: Supplementary file 6 [file emmm0007-1119-sd6.zip › Fig 1 source data .pdf]

Figure 2F

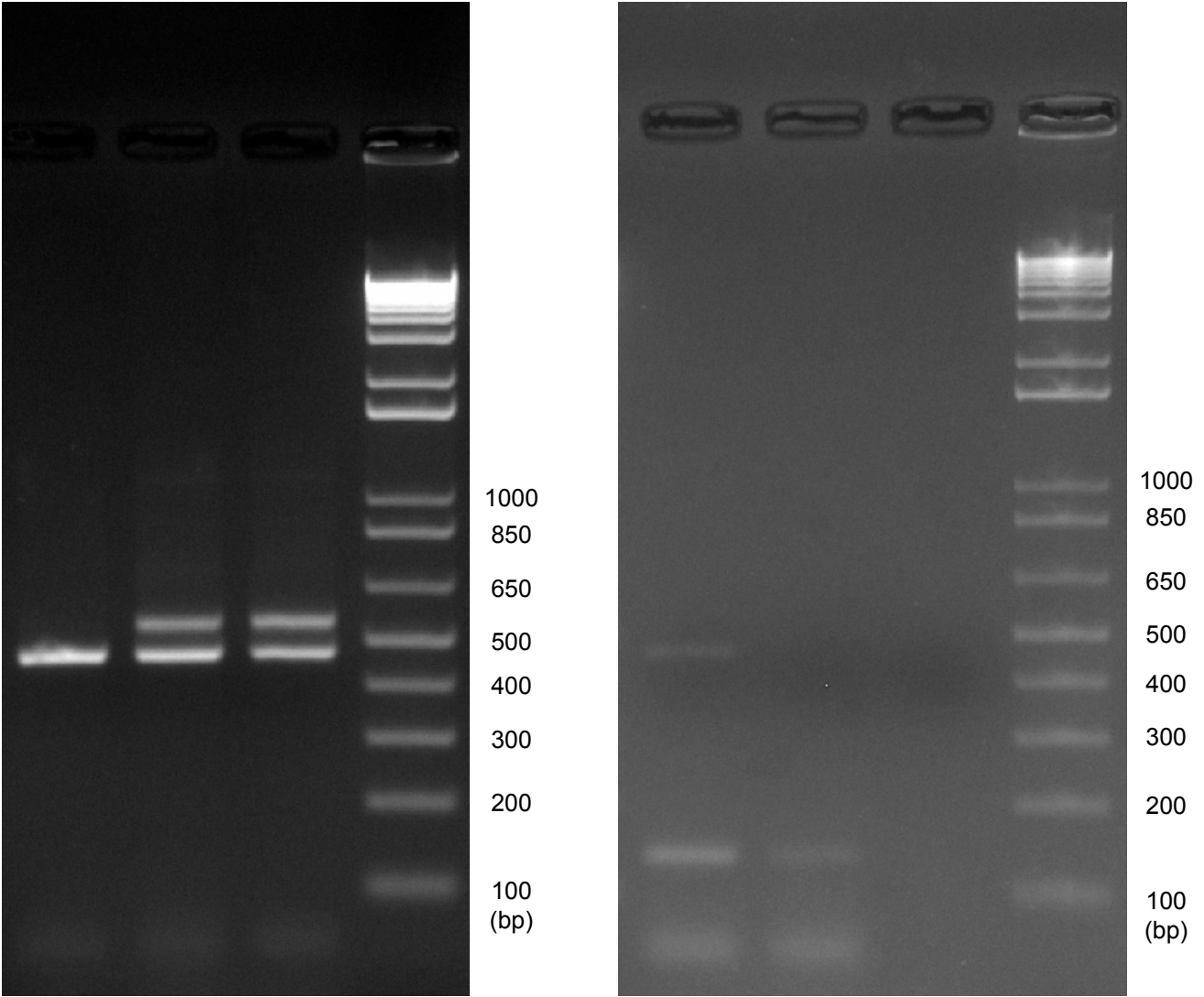

Supplement: Supplementary file 7 [file emmm0007-1119-sd7.pdf]

Figure 3C

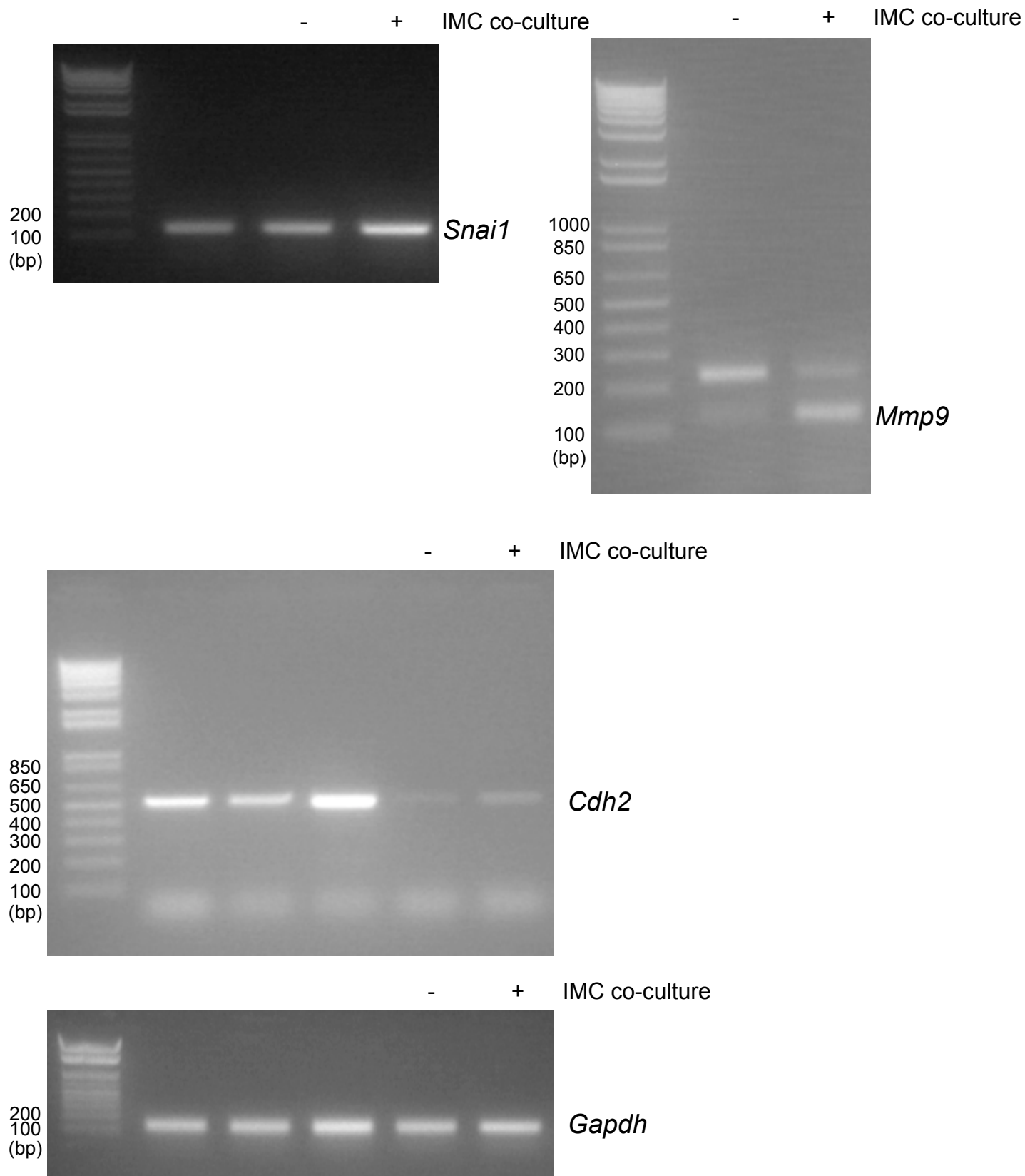

Figure 3D

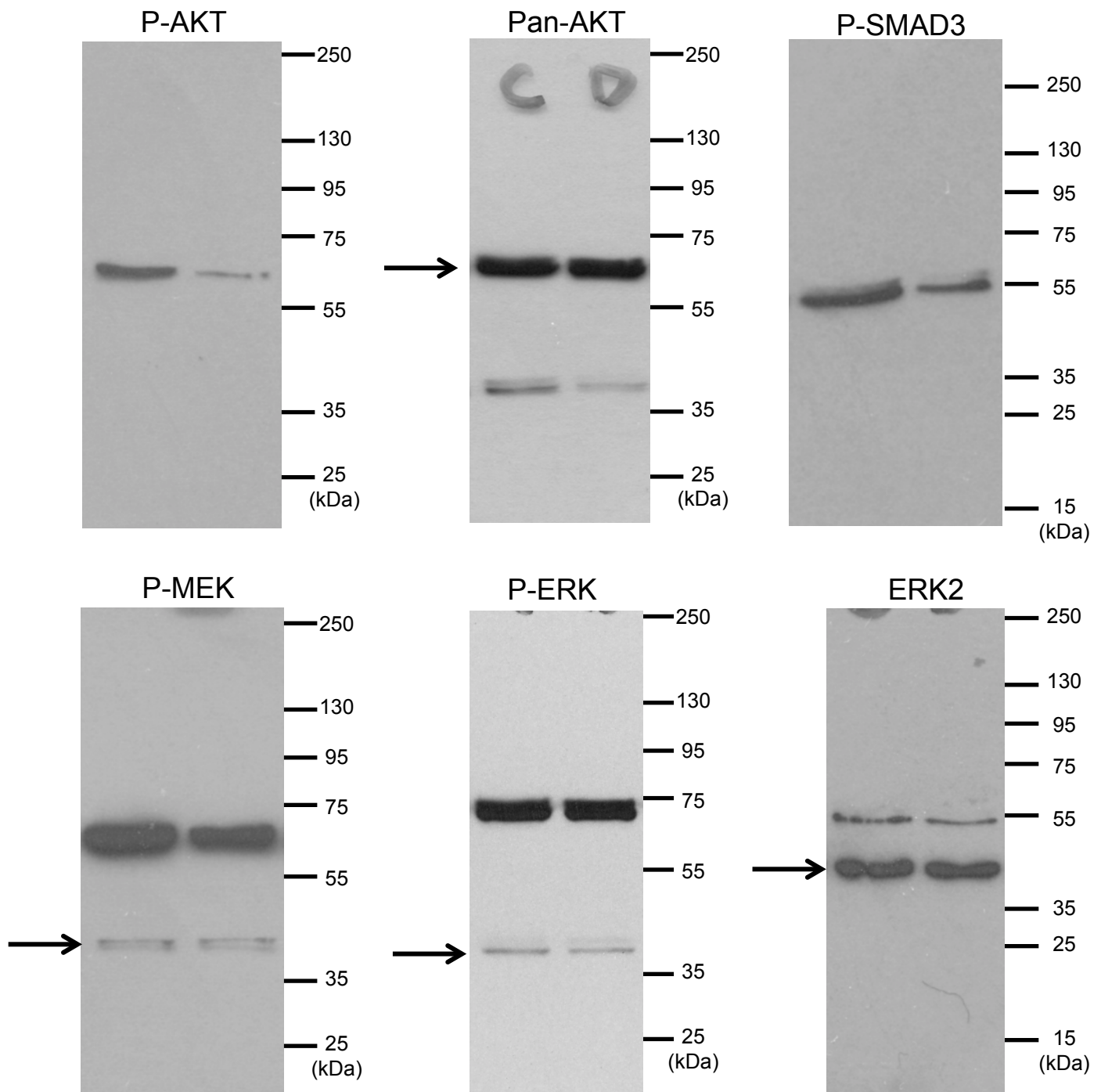

Supplement: Supplementary file 8 [file emmm0007-1119-sd8.zip › Figure 3 source data.pdf]

Figure 5A

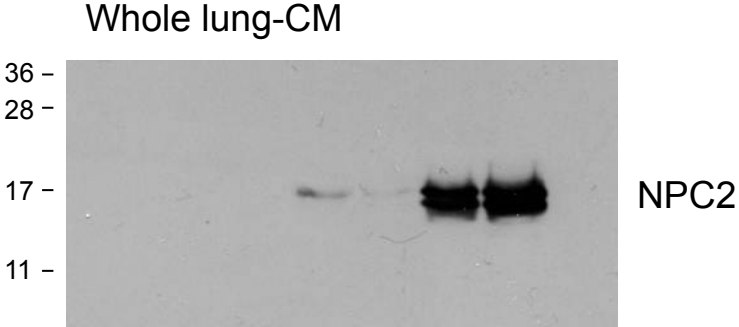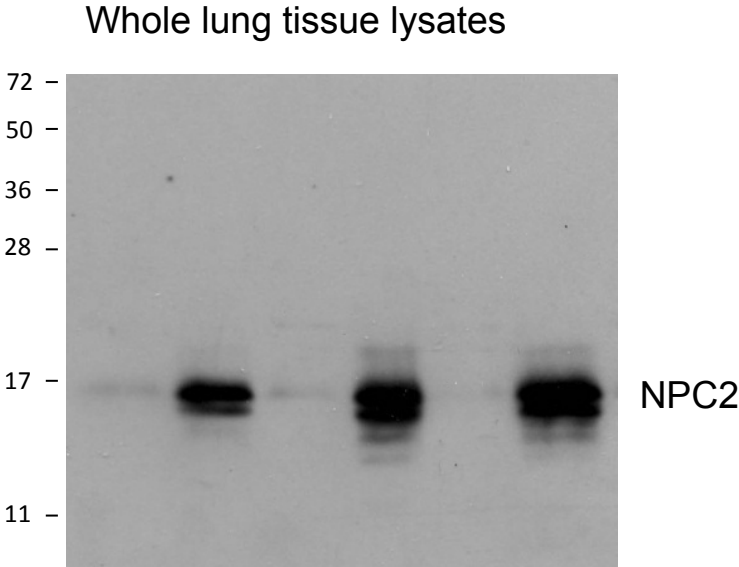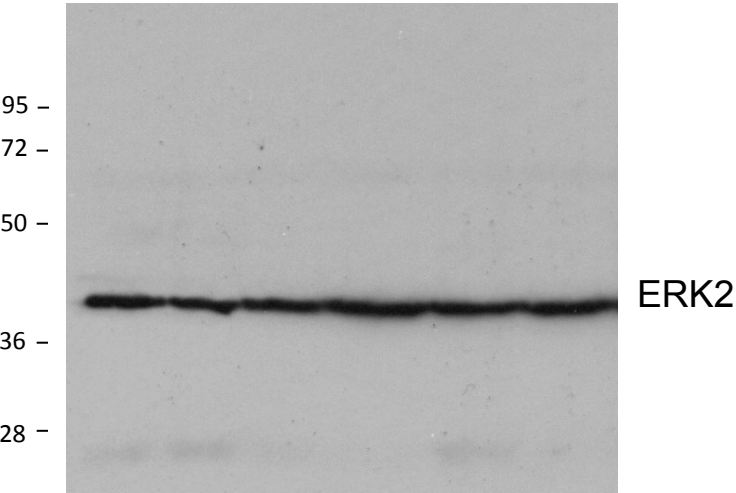

Figure 5B

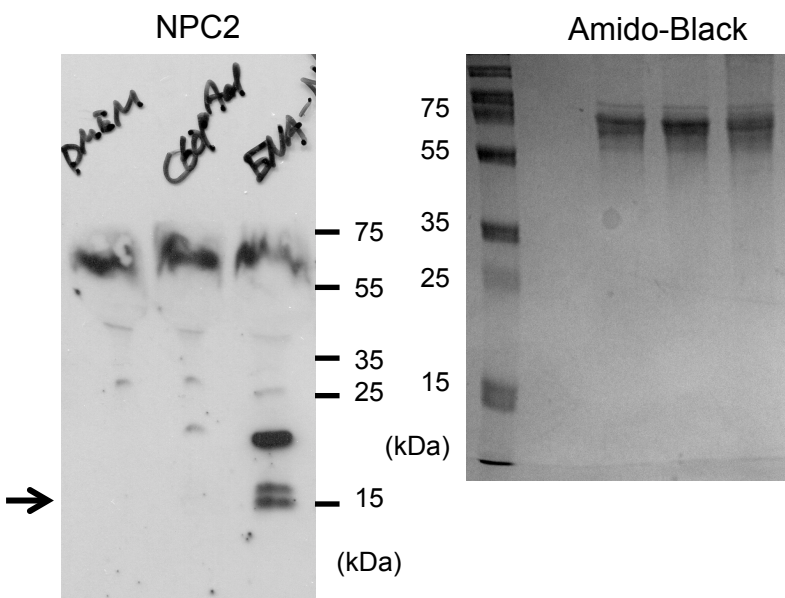

Figure 5D

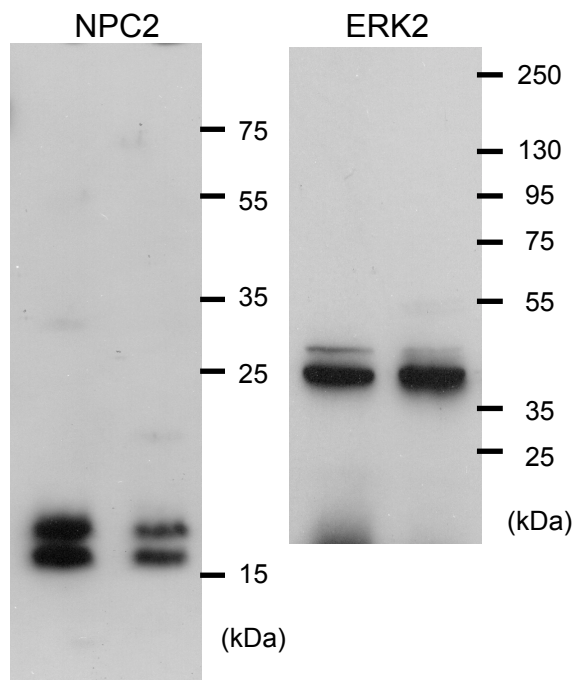

Figure 5E

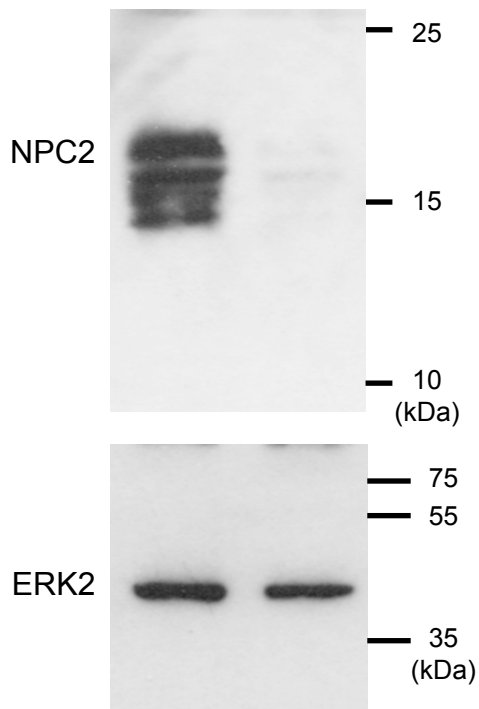

Figure 5F

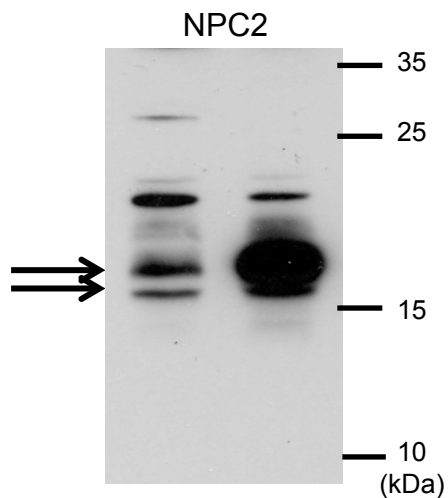

Supplement: Supplementary file 10 [file emmm0007-1119-sd10.zip › Figure 5 source data.pdf]

Figure 8A

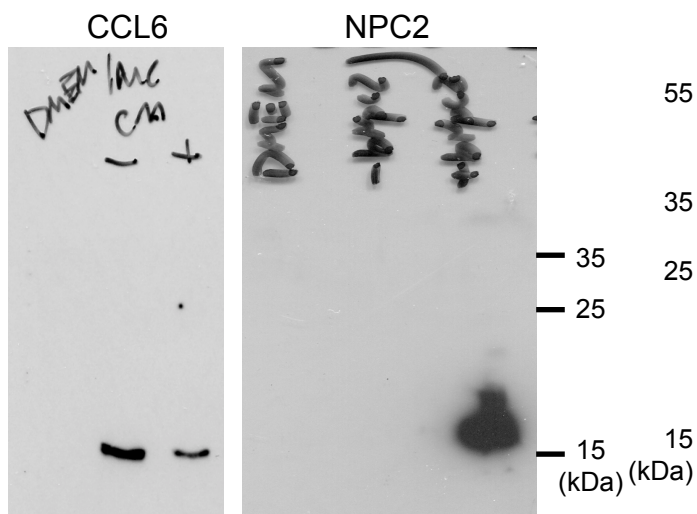

Figure 8C

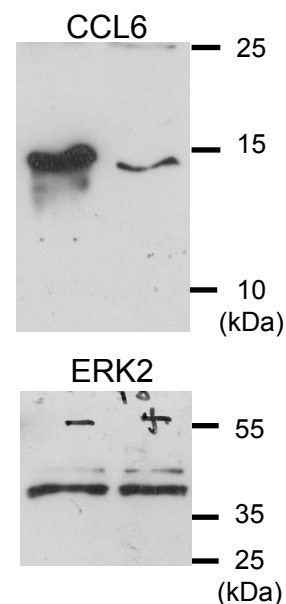

Figure 8E

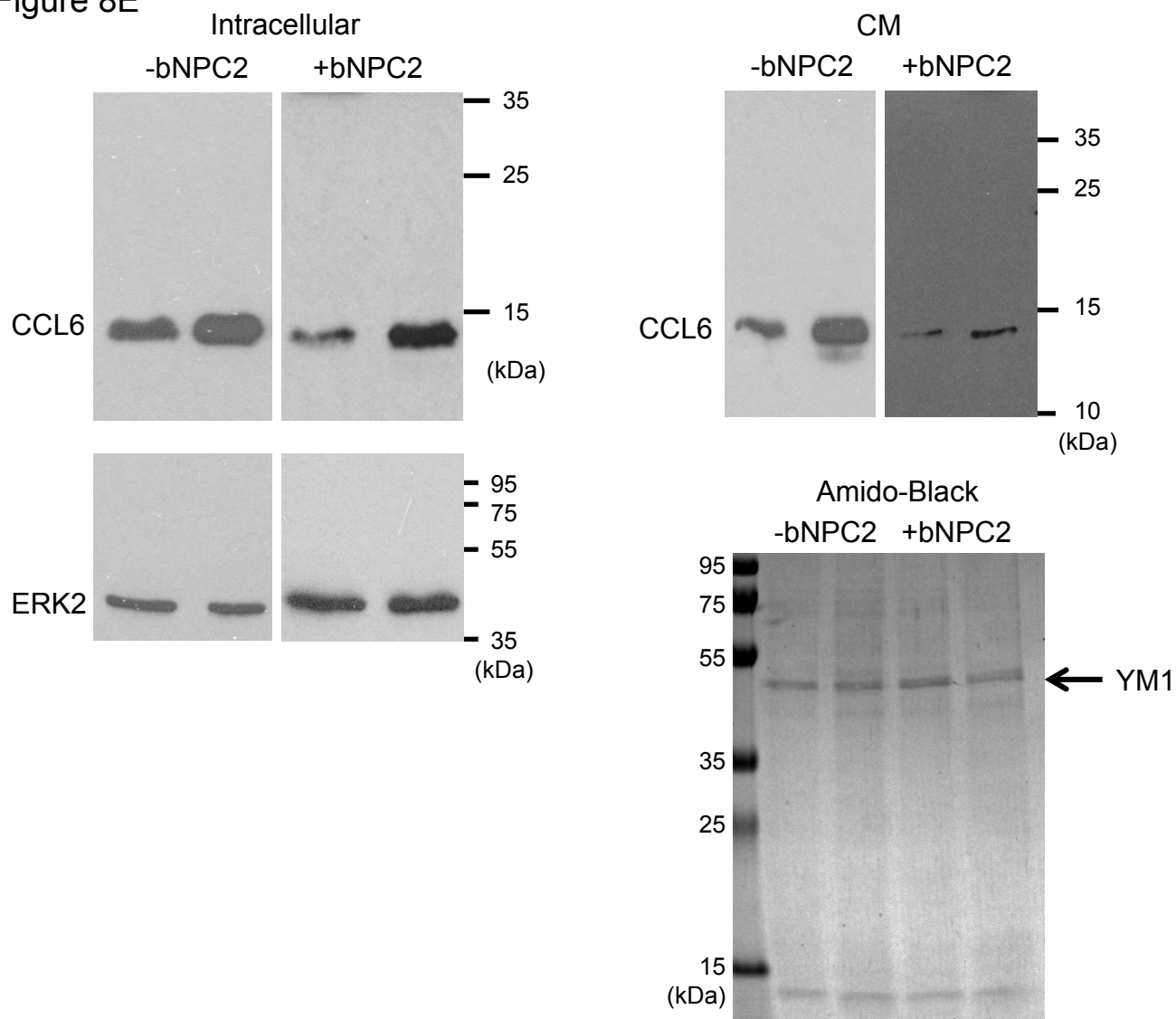

Supplement: Supplementary file 13 [file emmm0007-1119-sd13.zip › Figure 8 source data.pdf]
